# Effects of *Amomum tsaoko* on Chicken and Beef Quality During Stewing: Insights from Microstructure, Proteomics, and Molecular Docking

**DOI:** 10.3390/foods15203659

**Published:** 2026-10-15

**Authors:** Siyu Zhou, Shikuan Zhao, Rui Zhou, Cheng Gong, Yuanfeng Chen, Huiqing Luo, Zezhu Du, Yan Shen, Lingrui Lu, Shengjie Duan, Jinya Dong, Ruijuan Yang, Chongye Fang, Yunfei Ge

**Affiliations:** 1 College of Food Science and Technology, Yunnan Agricultural University, Kunming 650201, China; zzz121300y@163.com (S.Z.); 15153216972@163.com (S.Z.); 18088435058@163.com (R.Z.); 19184056918@163.com (Y.C.); 18788107018@163.com (H.L.); pj18213743420@163.com (Z.D.); 18184494648@163.com (Y.S.); w2414510451@163.com (S.D.); dongjinyaya@163.com (J.D.); helen_4680@163.com (R.Y.); 2 College of Agronomy and Biotechnology, Yunnan Agricultural University, Kunming 650201, China; triumph147@163.com (C.G.); 15087026316@163.com (L.L.)

**Keywords:** *Amomum tsaoko*, meat tenderness, shear force, proteomics, molecular docking

## Abstract

Meat tenderness is a key determinant of consumer acceptance, yet the meat type-dependent effects of *Amomum tsaoko* (*A. tsaoko*) during stewing remain unclear. This study investigated the effects of *A. tsaoko* powder on stewed chicken and beef using physicochemical analysis, LF-NMR, TEM, DIA-based proteomics, and molecular docking. *A. tsaoko* showed a significant meat type × treatment interaction for shear force (*p* < 0.001), decreasing shear force in chicken while increasing it in beef. No significant treatment effects were observed on total moisture content or the relative proportions of major T_2_ water populations. Qualitative TEM observations showed treatment-associated differences in myofibrillar organization. Proteomic screening identified 57 and 169 candidate DAPs in chicken and beef, respectively. Chicken DAPs were mainly associated with cytoskeletal organization, whereas beef DAPs showed broader associations with myofibrillar organization, muscle contraction, mitochondrial function, and energy metabolism. These proteomic patterns were considered exploratory because nominal *p*-values without multiple-testing correction were used. Catechin showed predicted binding energies of −7.1 kcal/mol with gelsolin in chicken and −9.4 kcal/mol with HSP60 in beef. Overall, the findings suggest meat-specific structural and cellular responses to *A. tsaoko*, while docking provided exploratory support for potential phenolic–protein interactions.

## 1. Introduction

*Amomum tsaoko* (*A. tsaoko*), a perennial herbaceous plant belonging to the Zingiberaceae family, is mainly cultivated in southern China, particularly in Yunnan, Guangxi, and Guizhou provinces [1,2]. Its fruit contains various bioactive constituents, including volatile terpenoids, organic acids, flavonoids, and phenolic acids, which have been associated with antioxidant, antimicrobial, and other biological activities [3,4,5]. Owing to its characteristic aroma and flavor, *A. tsaoko* is widely used as a spice and flavoring ingredient in traditional cuisines [6,7].

In meat processing, *A. tsaoko* is commonly used in stewing and marinating to reduce undesirable odors and improve flavor. However, its systematic effects on meat quality attributes, particularly tenderness, remain poorly understood. A recent review suggested that the effects of *A. tsaoko* essential oil on meat texture may depend on factors such as dosage, meat type, and processing conditions, highlighting the need to optimize its application for different meat products [8]. More importantly, the response to *A. tsaoko* may vary among different meat matrices because chicken and beef differ markedly in muscle structure, protein composition, and physicochemical properties. However, direct comparative evidence under comparable cooking conditions remains limited, and the structural and protein-level changes associated with such meat type-dependent responses have not been systematically explored.

Differences in muscle structural and compositional characteristics may contribute to meat type-specific responses during processing. Muscle fiber characteristics, including fiber type composition, size, and metabolic and contractile properties, are closely associated with meat quality [9,10]. Moreover, phenolic compounds can interact with muscle proteins and modify their structural and functional properties, thereby potentially affecting the texture and flavor of muscle foods. Oxidative and antioxidative processes can also alter muscle protein functionality and consequently influence meat quality [9,10]. These observations suggest that the effects of *A. tsaoko* on meat texture may depend not only on its bioactive constituents but also on the intrinsic characteristics and protein responses of different meat matrices. However, an integrated evaluation linking textural responses, water distribution, muscle ultrastructure, proteomic changes, and potential phenolic–protein interactions is still lacking.

Therefore, we hypothesized that *A. tsaoko* would exert meat type-dependent effects on tenderness during stewing. In this study, chicken breast and beef longissimus lumborum were cooked with or without dried *A. tsaoko* powder under controlled conditions. Physicochemical properties, water distribution, and muscle ultrastructure were evaluated, while DIA-based proteomic analysis was used to identify candidate treatment-responsive proteins and associated biological processes. Four representative phenolic compounds from *A. tsaoko* were further docked with selected candidate proteins to explore potential ligand–protein interactions. By integrating physicochemical analysis, LF-NMR, ultrastructural observation, proteomics, and molecular docking, this study aimed to characterize the differential responses of chicken and beef to *A. tsaoko* during stewing and to explore the structural and protein-level changes potentially associated with these differences.

## 2. Materials and Methods

### 2.1. Materials

Chicken breast and beef longissimus lumborum muscles were purchased from Ciba Farmers Market (Panlong District, Kunming, China). Because the meat samples were obtained from a commercial farmers’ market, detailed information regarding animal breed, age, sex, exact post-mortem interval, post-mortem handling, and initial storage history was unavailable. Commercially prepared *Amomum tsaoko* powder was provided by Nujiang Fuguo Company (Fugong, Yunnan, China). Information regarding the drying temperature and duration was not available from the supplier. The moisture content of the powder was 8.67 ± 0.39% (*n* = 3), as determined using an HS153 moisture analyzer (Mettler Toledo, Shanghai, China). Before use, the powder was passed through a 60-mesh sieve and stored in sealed nylon bags at room temperature. Potable water complying with the Chinese National Standard for Drinking Water Quality (GB 5749-2022) [11] was used throughout the experiments.

### 2.2. Sample Handling and Preparation

The samples were divided into four groups: control chicken (CK), *A. tsaoko*-treated chicken (ATC), control beef (CB), and *A. tsaoko*-treated beef (ATB). For each meat type, three independent biological replicates were included in both the control and treatment groups (*n* = 3 per group). Chicken breast and beef longissimus lumborum muscles were cut into uniform blocks (8 cm × 6 cm × 1 cm), rinsed for 5 min to remove residual blood and surface impurities, drained, and blanched for 5 min.

After blanching, the samples were cooked in separate pots of the same specifications. For the treatment groups, 10 g of *A. tsaoko* powder was added to 1.2 L of potable water before heating, whereas the control groups were prepared with 1.2 L of potable water without *A. tsaoko*. The cooking liquid was first brought to boiling, after which the meat samples were added and simmered at a heating power of 1000 W for 45 min. The meat samples were portioned to approximately the same volume, and the water volume, heating power, and cooking time were kept consistent among groups to minimize variation during cooking. After cooking, the samples were cooled at room temperature for 10 min before most physicochemical analyses. For cooking loss measurement, the samples were cooled for 20 min as described in the corresponding subsection. The remaining samples were stored at −80 °C for subsequent analyses.

### 2.3. Physicochemical Property Analysis

#### 2.3.1. pH

The pH of each stewed meat sample was measured in triplicate using a carcass pH meter (MATTHAUS, Eckelsheim, Germany), according to a previously described method [12].

#### 2.3.2. Color Measurement

Meat color was measured using a CR-410 colorimeter (Konica Minolta, Inc., Osaka, Japan) according to the method of Nuñez et al. [13], with minor modifications. Specifically, a CR-410 colorimeter was used instead of the CR-400 instrument described in the referenced method, and measurements were taken at three randomly selected locations on the surface of each replicate sample rather than at the center of the sample. The CIE L*, a*, and b* values were recorded to represent lightness, redness, and yellowness, respectively. The mean of the three measurements was used for subsequent analysis.

#### 2.3.3. Shear Force

Shear force was determined following Zhang et al. [14], with slight modifications, using a TA.XT Plus Texture Analyzer (Stable Micro Systems, Godalming, UK) equipped with an HDP/BSW Warner–Bratzler blade, according to NY/T 1180-2006 [15]. Stewed meat samples were cut into 2 cm × 1 cm × 1 cm pieces. In the instrument software, the test mode was set to compression, which defines the downward movement of the probe. However, because the HDP/BSW attachment is a Warner–Bratzler blade, the downward movement of the blade through the sample produced a cutting/shearing action rather than conventional flat-platen compression. The pre-test, test, and post-test speeds were all set at 2.0 mm/s, with a test distance of 40 mm and a test duration of 10 s. The peak force recorded during blade passage through the sample was reported as the Warner–Bratzler shear force.

#### 2.3.4. Moisture Content

The moisture content of the meat samples was measured using an HS153 halogen moisture analyzer (Mettler Toledo, Shanghai, China), with reference to the method described, in which 0.5 g of each sample was analyzed at 105 °C.

#### 2.3.5. *Cooking Loss*

Samples used for general physicochemical analyses were cooled at room temperature for 10 min after cooking. For *cooking loss* determination, however, samples were cooled at room temperature for 20 min before weighing, according to the method of Jo et al. [16]. *Cooking loss* was calculated from the sample weight before cooking (*m*_0_) and the weight after cooking and 20 min of cooling (*m*_1_) as follows:

(1) $$Cooking\;loss(\%)=\frac{m_{0}-m_{1}}{m_{0}}\times 100$$

#### 2.3.6. *Centrifugal Loss*

*Centrifugal loss* was measured with reference to the method described by Yu et al. [17], with slight modifications. Each sample, 4.00 g, was wrapped in filter paper and centrifuged at 4000× *g* for 10 min at 4 °C. The weights before and after centrifugation were recorded as *m*_0_ and *m*_1_, respectively, and *centrifugal loss* was calculated using Equation (2):

(2) $$Centrifugal\;loss(\%)=\frac{m_{0}-m_{1}}{m_{0}}\times 100$$

### 2.4. Low-Field NMR Measurement and Analysis

Low-field nuclear magnetic resonance (LF-NMR) measurements were performed using a MicroMR12-025V NMR analyzer (Suzhou Niumag Analytical Instrument Corporation, Suzhou, China) equipped with a 25-mm-diameter detection coil. NMR analysis software (Version 4.0, Suzhou Niumag Analytical Instrument Corporation) was used for data acquisition and analysis. Before measurement, surface moisture was gently removed from the meat samples using weighing paper, and approximately 1.1–2.3 g of sample was placed in the detection coil. A standard oil sample was first used to determine the center frequency. The transverse relaxation time (T_2_) distribution was measured using a Carr–Purcell–Meiboom–Gill (CPMG) pulse sequence at a proton resonance frequency of 12 MHz. The acquisition parameters were as follows: 90° pulse width (P1), 6.6 μs; 180° pulse width (P2), 10.64 μs; waiting time (TW), 4500 ms; echo time (TE), 0.15 ms; number of echoes (NECH), 7000; number of scans (NS), 4; spectral width (SW), 200 kHz; and receiver gain, 20 dB. Measurements were conducted under ambient laboratory temperature without active temperature control. The acquired decay data were subjected to 10,000 inversion iterations to obtain the T_2_ distribution curves, which were used to characterize the relative proportions and mobility of different water populations in the meat samples.

### 2.5. Transmission Electron Microscopy (TEM)

The ultrastructure of the stewed meat samples was examined by transmission electron microscopy (TEM). Briefly, samples were cut into approximately 1 mm^3^ blocks and immediately fixed in electron microscopy fixative (G1102, Biosharp, Hefei, Anhui, China) at 4 °C. The fixed samples were rinsed three times with 0.1 M phosphate buffer (PB, pH 7.4) for 15 min each, post-fixed with 1% osmium tetroxide in 0.1 M PB for 2 h in the dark, and rinsed again with PB [18].

The samples were dehydrated through a graded ethanol series (30%, 50%, 70%, 80%, 95%, and 100%), followed by acetone treatment. They were subsequently infiltrated with acetone/812 resin mixtures, embedded in 812 resin, and polymerized at 60 °C for 48 h. Ultrathin sections (60–80 nm) were prepared using an ultramicrotome (Leica UC7, Leica Microsystems GmbH, Wetzlar, Germany), collected on 150-mesh Formvar-coated copper grids, and stained with uranyl acetate and lead citrate. The sections were examined using a transmission electron microscope (FEI G2, FEI Company, Hillsboro, OR, USA) [19].

For each sample, seven images were acquired at each of three magnifications (3200×, 6500×, and 11,000×), resulting in a total of 21 TEM images per sample. Representative images acquired at 6500× magnification are presented in the manuscript for qualitative comparison of muscle ultrastructure.

### 2.6. Proteomic Analysis

Proteomic analysis was performed using a data-independent acquisition (DIA)-based LC-MS/MS workflow. Proteins were extracted from the samples using a lysis buffer containing 50 mM Tris (pH 8.1), 1% SDS, and a mixture of protease and phosphatase inhibitors, including sodium pyrophosphate, β-glycerophosphate, sodium orthovanadate, sodium fluoride, EDTA, and leupeptin. Protein concentrations were determined using the bicinchoninic acid (BCA) assay. For each sample, 20 μg of total protein was subjected to denaturation, reduction, and alkylation, followed by digestion with trypsin at 37 °C for 2 h. After termination of digestion, the resulting peptides were desalted using C18 columns, concentrated at 45 °C, and reconstituted for LC-MS/MS analysis [20].

Peptides were separated using a Vanquish Neo UHPLC system (Thermo Fisher Scientific, Germering, Germany) equipped with a PepMap Neo trap column and a μPAC Neo High Throughput analytical column. Mobile phase A consisted of 0.1% formic acid in water, whereas mobile phase B consisted of 0.1% formic acid in 80% acetonitrile. The column was equilibrated with 96% mobile phase A and 4% mobile phase B and maintained at 50 °C. Peptides were separated at a constant flow rate of 2.5 μL/min over a total run time of 8.0 min using the following gradient: 4–25% B from 0 to 4.8 min, 25–35% B from 4.8 to 6.9 min, 35–99% B from 6.9 to 7.3 min, and 99% B from 7.3 to 8.0 min. A total of 500 ng of peptides was loaded for each LC-MS/MS analysis. Because peptide concentrations varied among samples, the injection volume was adjusted accordingly to ensure a consistent peptide loading amount of 500 ng for all samples.

Mass spectrometric analysis was performed using an Orbitrap Astral mass spectrometer operated in positive-ion DIA mode. The electrospray voltage was set to 1.9 kV, and the precursor-ion scan range was m/z 380–980. Full MS scans were acquired at a resolution of 240,000, with a normalized AGC target of 500% and a maximum injection time of 3 ms. DIA MS/MS spectra were acquired at a resolution of 80,000, with a normalized AGC target of 500% and a maximum injection time of 3 ms. The RF-lens amplitude was set to 40%. Higher-energy collisional dissociation (HCD) was used for precursor fragmentation with a normalized collision energy of 25%. The DIA isolation window was set to 2 Th, and the cycle time was 0.6 s.

Raw data were processed using DIA-NN v1.9 against species-specific UniProt reference proteomes for Bos taurus (UP000009136) and Gallus gallus (UP000000539). Trypsin was specified as the digestion enzyme with up to two missed cleavages, and precursor- and protein-level false discovery rates were controlled at 1%.

Differentially abundant proteins (DAPs) were defined as proteins with fold change >1.5 or <0.667 and *p* < 0.05 based on Student’s *t*-test. Nominal (raw) *p*-values were used for differential-abundance testing, and no multiple-testing correction was applied. DAPs were subjected to hierarchical clustering, subcellular localization prediction using WoLF PSORT, GO annotation using Blast2GO, and KEGG annotation using eggNOG-mapper with DIAMOND. Functional enrichment was assessed using the hypergeometric test. Nominal (raw) *p*-values were used for enrichment analysis, with *p* < 0.05 used to identify enriched terms or pathways; no multiple-testing correction was applied.

### 2.7. Molecular Docking

Four representative treatment-responsive DAPs were selected for exploratory molecular docking, including A0A8V1ACU4 and A0A8V1AL60 from chicken and A0AAA9TBS7 and P61603 from beef. All four selected proteins exhibited fold changes (FCs) greater than 1.5 with *p* < 0.05 (*t*-test). In addition to these differential-abundance characteristics, functional relevance to the major biological processes highlighted by the proteomic analysis for the corresponding meat type was considered when selecting representative candidates. Specifically, proteins related to cytoskeletal/structural processes were considered in chicken, whereas proteins related to mitochondrial function and protein homeostasis were considered in beef. No additional prespecified quantitative ranking algorithm was applied to rank all eligible DAPs specifically for docking.

The three-dimensional structures of the receptor proteins were generated using SWISS-MODEL based on amino acid sequences retrieved from UniProt [21]. For each model, the template information, sequence identity, model coverage, Global Model Quality Estimate (GMQE), and QMEANDisCo global score were recorded to evaluate model quality. Model coverage was determined from the target–template alignment information generated by SWISS-MODEL. The stereochemical quality of each model was further evaluated by Ramachandran plot analysis using PROCHECK implemented in SAVES v6.0. The percentages of residues located in the most favoured, additional allowed, generously allowed, and disallowed regions were recorded. Only models with more than 90% of residues located in the combined most favoured, additional allowed, and generously allowed regions were retained for subsequent docking analysis. Detailed model-quality parameters are provided in Table S3.

Caffeic acid, gallic acid, catechin, and protocatechuic acid, which have been reported as phenolic constituents of *A. tsaoko* [2], were selected as representative ligands. The three-dimensional structures of the compounds were obtained from PubChem and imported into PyRx (version 0.8), where they were energy-minimized using the integrated Open Babel software (version 2.2.3) and converted to PDBQT format. Receptor structures were prepared using AutoDockTools 1.5.7 by removing water molecules, adding hydrogen atoms, assigning Gasteiger charges and AutoDock4 atom types, and converting the structures to PDBQT format.

Molecular docking was performed using AutoDock Vina implemented in PyRx. Because no predefined binding site was specified, a blind-docking strategy was adopted, with the search space expanded to encompass the entire receptor structure. The receptor-specific grid centers and dimensions are provided in Table S4. The exhaustiveness was set to 8 for all docking calculations. Up to nine binding poses were retained for each docking calculation, and the pose with the lowest predicted binding energy was selected for subsequent interaction analysis. The resulting receptor–ligand complexes were visualized using PyMOL (version 3.1.3.1), and two-dimensional interaction diagrams were generated using BIOVIA Discovery Studio 4.5 to examine predicted non-covalent interactions, including hydrogen bonds and hydrophobic interactions.

### 2.8. Statistical Analysis

Data are expressed as mean ± standard deviation (SD) from three independent biological replicates (*n* = 3), unless otherwise stated. Main-effect values in Table 1 are presented as estimated marginal means ± standard error (SE). The effects of meat type, treatment, and their interaction were analyzed using two-way analysis of variance (ANOVA). When a significant interaction was detected, simple-effects comparisons were performed using Šídák’s adjustment for multiple comparisons. Statistical analyses were conducted using GraphPad Prism version 10.6.0 (GraphPad Software, Boston, MA, USA), with *p* < 0.05 considered statistically significant.

**Table 1:** Main effects of meat type and *A. tsaoko* treatment on the physicochemical properties of chicken and beef.

Source	Level	Moisture (%)	Cooking Loss (%)	Centrifugal Loss (%)	Shear Force (gf)	pH	L*	a*	b*
Meat type	Chicken	62.75 ± 0.74 ^a^	40.09 ± 1.72 ^b^	19.96 ± 1.05 ^a^	2504.20 ± 181.00 ^b^	6.19 ± 0.06 ^a^	42.75 ± 0.66 ^a^	9.03 ± 0.28 ^b^	14.83 ± 0.35 ^a^
	Beef	57.44 ± 0.74 ^b^	51.29 ± 1.72 ^a^	7.17 ± 1.05 ^b^	5328.02 ± 181.00 ^a^	6.21 ± 0.06 ^a^	35.96 ± 0.66 ^b^	10.42 ± 0.28 ^a^	10.90 ± 0.35 ^b^
Treatment	Control	60.67 ± 0.74 ^a^	48.20 ± 1.72 ^a^	14.68 ± 1.05 ^a^	3638.27 ± 181.00 ^a^	6.27 ± 0.06 ^a^	40.20 ± 0.66 ^a^	9.35 ± 0.28 ^a^	13.06 ± 0.35 ^a^
	*A. tsaoko*	59.53 ± 0.74 ^a^	43.18 ± 1.72 ^a^	12.46 ± 1.05 ^a^	4193.95 ± 181.00 ^a^	6.13 ± 0.06 ^a^	38.50 ± 0.66 ^a^	10.10 ± 0.28 ^a^	12.67 ± 0.35 ^a^
ANOVA	Meat type	***	**	***	***	ns	***	**	***
	Treatment	ns	ns	ns	ns	ns	ns	ns	ns
	Meat type × Treatment	ns	ns	ns	***	ns	ns	ns	ns

Note: Main-effect values are presented as estimated marginal means ± standard error (SE). Different lowercase superscript letters within each factor and column indicate significant differences between means (*p* < 0.05), whereas identical letters indicate no significant difference. The effects of meat type, treatment, and their interaction were evaluated using two-way analysis of variance (ANOVA). ns, not significant (*p* ≥ 0.05); **, *p* < 0.01; ***, *p* ≤ 0.001. Because a significant meat type × treatment interaction was detected for shear force, Šídák-adjusted simple-effects comparisons were performed and are presented separately in Table 2.

**Table 2:** Multiple comparisons of shear force following the significant meat type × treatment interaction.

Meat Type	Control	*A. tsaoko*
Chicken	3680.37 ± 564.41 ^aA^	1328.03 ± 297.14 ^bB^
Beef	3596.17 ± 212.55 ^bA^	7059.87 ± 578.16 ^aA^

Note: Values are expressed as mean ± SD (*n* = 3). Different lowercase superscript letters indicate significant differences between treatments within the same meat type, whereas different uppercase superscript letters indicate significant differences between meat types within the same treatment (*p* < 0.05), based on Šídák-adjusted simple-effects comparisons following two-way ANOVA.

## 3. Results and Discussion

### 3.1. Physicochemical Properties

The physicochemical properties of the stewed meat samples are presented in Table 1. Two-way ANOVA showed that meat type significantly affected moisture content, cooking loss, centrifugal loss, shear force, and the L*, a*, and b* values (*p* < 0.05), whereas no significant effect was observed for pH. In contrast, the main effect of *A. tsaoko* treatment was not significant for any of the measured parameters. A significant meat type × treatment interaction was detected only for shear force (*p* < 0.001), indicating that the effect of *A. tsaoko* treatment on shear force differed between chicken and beef. Šídák-adjusted simple-effects comparisons further showed that *A. tsaoko* treatment significantly decreased shear force in chicken, from 3680.37 ± 564.41 gf in the control group to 1328.03 ± 297.14 gf in the treated group (*p* = 0.0004), whereas it significantly increased shear force in beef, from 3596.17 ± 212.55 gf to 7059.87 ± 578.16 gf (*p* < 0.0001) (Table 2). Under the control condition, shear force did not differ significantly between chicken and beef (*p* = 0.9683), whereas following *A. tsaoko* treatment, beef exhibited a significantly higher shear force than chicken (*p* < 0.0001). No significant treatment or meat type × treatment interaction effects were observed for moisture content, cooking loss, centrifugal loss, pH, or color parameters, indicating that the divergent shear-force responses were not accompanied by corresponding treatment-related changes in these bulk physicochemical properties.

The contrasting shear-force responses may be associated with inherent differences in the structural and biochemical characteristics of chicken and beef muscle. Meat tenderness is influenced by multiple factors, including myofibrillar integrity, muscle-fiber characteristics, proteolytic processes, and intramuscular connective tissue [22,23]. Therefore, differences in muscle organization and protein properties between chicken and beef may contribute to their different responses to the same *A. tsaoko* treatment. Previous studies have also shown that plant-derived ingredients can modify meat tenderness, although the magnitude and direction of the effects depend on the meat system and treatment conditions. For example, Zhang reported that papaya juice improved the tenderness of stewed Wenchang chicken, with the effect varying with juice concentration. Consistent with the multifactorial nature of meat tenderness, these findings indicate that the effects of plant-derived ingredients are influenced not only by the ingredient itself but also by the characteristics of the meat matrix. The opposite shear-force responses observed in chicken and beef in the present study further suggest that such effects should not be generalized across different meat types. This meat type-dependent response prompted further examination of water distribution, muscle ultrastructure, and proteomic alterations to gain further insight into the potential basis of the divergent textural changes.

### 3.2. Low-Field NMR

Low-field nuclear magnetic resonance (LF-NMR) is a rapid and non-destructive method for evaluating water status and distribution in food systems [24,25,26,27]. Because *A. tsaoko* treatment did not significantly affect total moisture content in the physicochemical analysis, LF-NMR was further used to examine whether the treatment altered water mobility or the relative distribution of different water populations. The transverse relaxation time, T_2_, reflects the mobility of hydrogen protons and can be used to distinguish different water populations in meat [28]. T_21_ (0.1–10 ms), T_22_ (10–100 ms), and T_23_ (100–1000 ms) were assigned to bound water, immobilized water, and free water, respectively [29,30], and their relative peak areas were used to estimate the proportions of the corresponding water populations.

**Figure 1:**
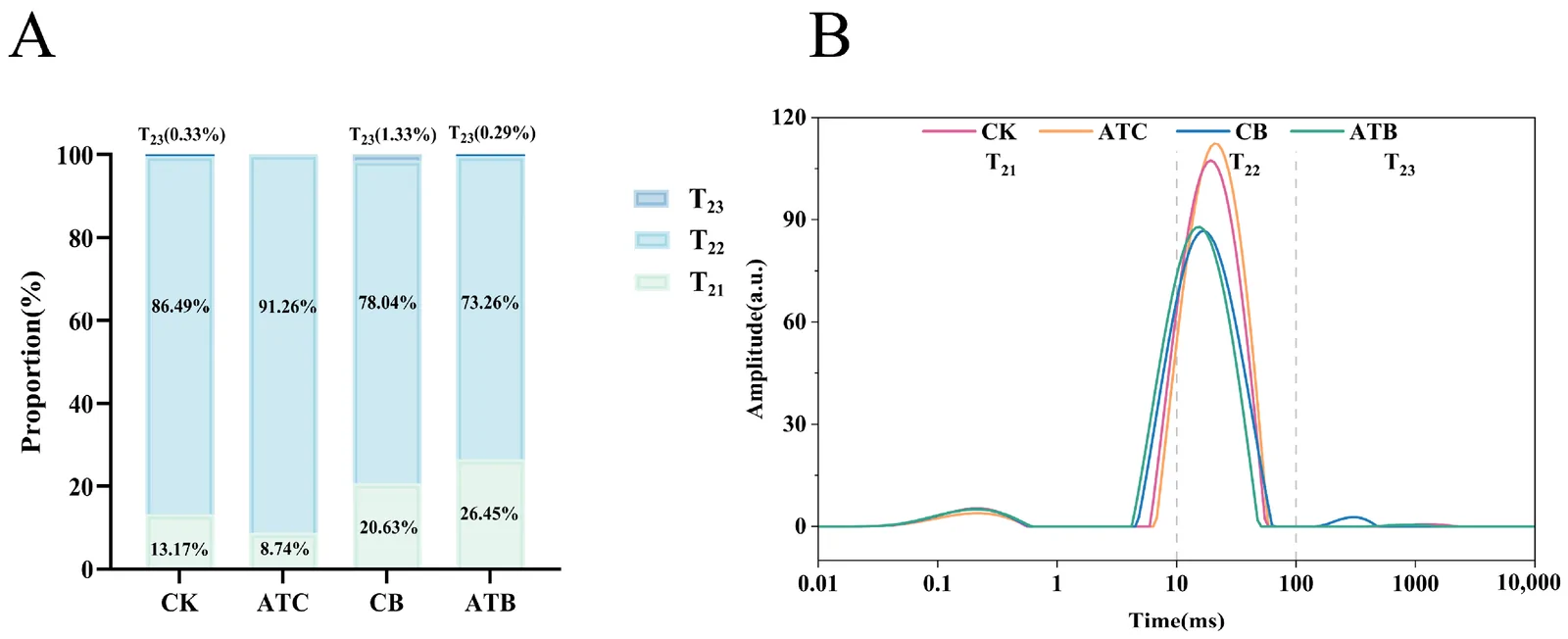
Water distribution of stewed chicken and beef determined by low-field nuclear magnetic resonance (LF-NMR). CK, chicken stewed without *A. tsaoko* powder; ATC, chicken stewed with *A. tsaoko* powder; CB, beef stewed without *A. tsaoko* powder; ATB, beef stewed with *A. tsaoko* powder. (**A**) Relative proportions of T_21_ (bound water), T_22_ (immobilized water), and T_23_ (free water) in the four groups. Statistical analysis was performed separately for each water population using two-way analysis of variance (ANOVA), with meat type and treatment as fixed factors. Meat type significantly affected T_21_ and T_22_ (both *p* < 0.0001), whereas the treatment effect and meat type × treatment interaction were not significant. No significant main or interaction effects were observed for T_23_ (*p* > 0.05). (**B**) Representative transverse relaxation time (T_2_) distributions of the four groups.

LF-NMR analysis showed that immobilized water (T_22_) was the predominant water population in all groups, accounting for 87.76%, 91.05%, 75.59%, and 70.47% in CK, ATC, CB, and ATB, respectively (Figure 1A). The corresponding T_21_ proportions were 12.14%, 8.91%, 23.93%, and 29.29%, whereas T_23_ accounted for only 0.11%, 0.03%, 0.49%, and 0.24%, respectively. Two-way ANOVA showed a significant main effect of meat type on the proportions of T_21_ and T_22_ (both *p* < 0.0001), whereas neither the main effect of treatment (T_21_, *p* = 0.6624; T_22_, *p* = 0.5958) nor the meat type × treatment interaction (T_21_, *p* = 0.0707; T_22_, *p* = 0.0802) was significant. For T_23_, no significant effects of meat type (*p* = 0.2206), treatment (*p* = 0.4864), or their interaction (*p* = 0.7055) were detected. These results indicate that the relative distribution of bound and immobilized water was primarily determined by meat type rather than by *A. tsaoko* treatment. The predominance of immobilized water is consistent with previous studies showing that most water in muscle is located within the myofibrillar matrix and exhibits relatively restricted mobility [31].

The T_2_ relaxation spectra further illustrated differences in water mobility among the four groups (Figure 1B). The predominant T_22_ peak of ATC was located at a slightly longer relaxation time than that of CK, whereas the T_22_ peak of ATB was shifted toward a shorter relaxation time relative to CB. In addition, CB exhibited a detectable T_23_ component at longer relaxation times. These spectral differences may reflect variations in the local physicochemical environment and interactions between water and muscle structural components. However, because the positions of the relaxation peaks were not subjected to statistical comparison, these shifts should be interpreted descriptively rather than as confirmed treatment effects. Importantly, the absence of significant treatment or meat type × treatment effects on the relative proportions of T_21_, T_22_, and T_23_ suggests that the opposite shear-force responses observed in chicken and beef were unlikely to be primarily explained by changes in water-population distribution. This finding therefore points to other meat-specific factors, particularly differences in muscle structural organization and protein-level responses, which were further examined by ultrastructural and proteomic analyses.

### 3.3. TEM

**Figure 2:**
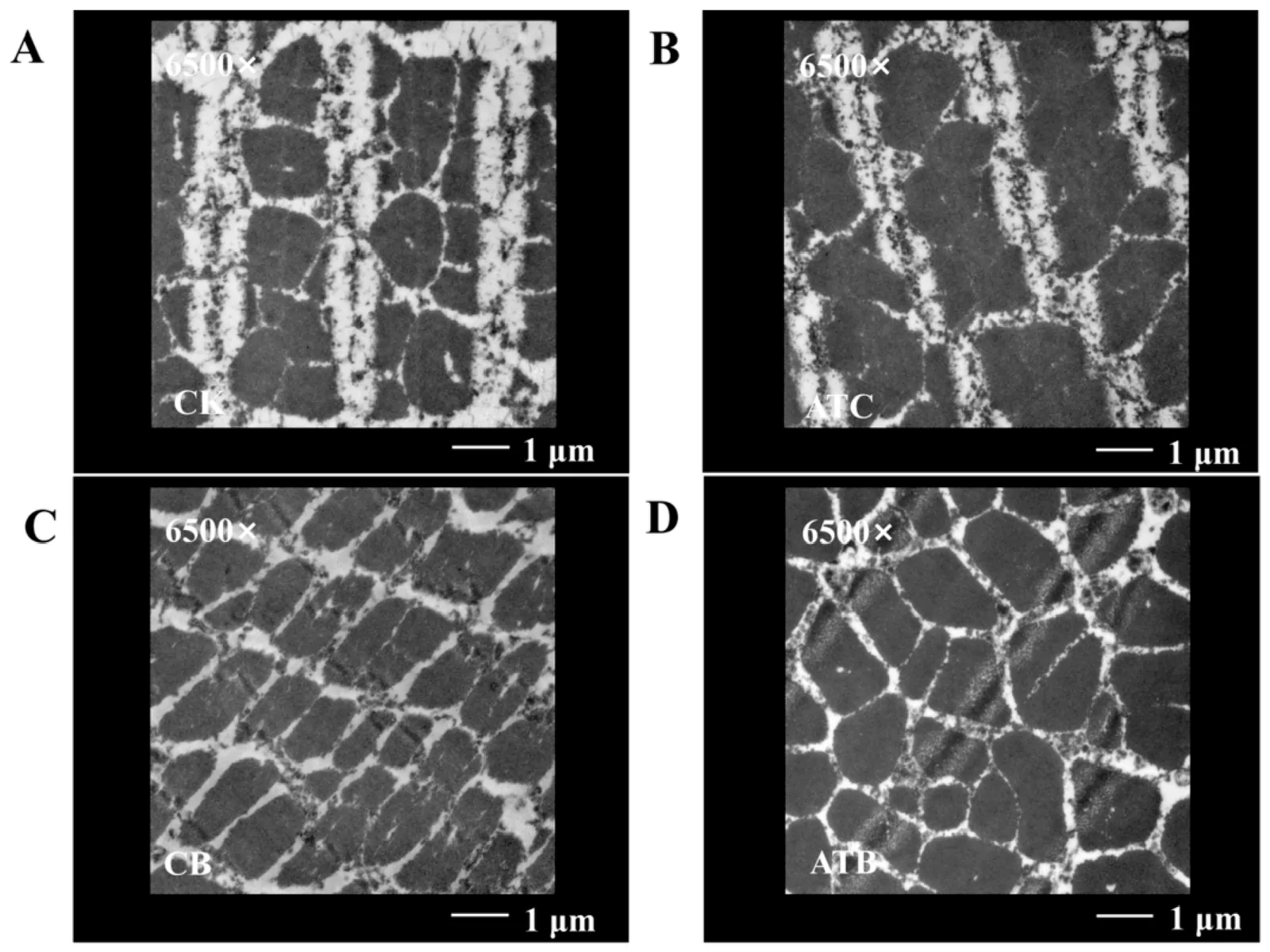
Transmission electron microscopy images of stewed chicken and beef. (**A**) CK, chicken stewed without *A. tsaoko* powder; (**B**) ATC, chicken stewed with *A. tsaoko* powder; (**C**) CB, beef stewed without *A. tsaoko* powder; and (**D**) ATB, beef stewed with *A. tsaoko* powder. Representative images are shown at 6500× magnification. Scale bar = 1 μm.

Because LF-NMR analysis did not reveal significant treatment-related changes in the relative proportions of the major water populations, TEM was further used to examine whether the contrasting shear-force responses were accompanied by alterations in muscle ultrastructure. As shown in Figure 2, differences in myofibrillar morphology were observed between the control and *A. tsaoko*-treated samples in both meat types. Electron-dense materials were observed within some intermyofibrillar spaces, consistent with ultrastructural features previously reported in cooked meat [32].

In chicken, ATC exhibited apparently wider intermyofibrillar spaces and a relatively looser myofibrillar arrangement compared with CK. These ultrastructural differences were accompanied by the lower shear force observed in ATC, which is consistent with a possible association between reduced myofibrillar compactness and lower resistance to mechanical shearing. Previous studies have shown that meat tenderness is influenced by the integrity and organization of myofibrillar and cytoskeletal structures [23], supporting the relevance of these structural observations to the textural response. Thus, the looser myofibrillar organization observed in ATC may provide a structural context for its lower shear force, although a direct causal relationship cannot be established from the present qualitative observations. However, because no quantitative morphometric analysis of the TEM images was performed, these structural differences should be interpreted as qualitative observations rather than statistically confirmed treatment effects.

In beef, differences in myofibrillar morphology and organization were also observed between CB and ATB, with ATB exhibiting a more irregular myofibrillar arrangement. However, the extent of the apparent ultrastructural alteration in beef was less pronounced than that observed between CK and ATC in chicken. Unlike in chicken, however, this structural alteration was accompanied by an increase rather than a decrease in shear force. This finding indicates that visually apparent myofibrillar disorganization does not necessarily correspond directly to lower shear resistance and that the contribution of ultrastructure to tenderness may depend on the specific structural and biochemical characteristics of the meat system. In beef, factors such as myofibrillar organization, connective tissue properties, and protein stability may act together in determining the final mechanical response [23,33]. This may partly explain why the relationship between ultrastructural appearance and shear force differed between chicken and beef.

Overall, although *A. tsaoko* treatment was associated with changes in muscle ultrastructure in both meat types, the contrasting shear-force responses suggest that ultrastructural changes alone may not fully explain the meat type-dependent effects of *A. tsaoko* on texture. Instead, the different relationships between ultrastructural appearance and shear force in chicken and beef suggest that additional protein-level and cellular processes may be involved. Proteomic analysis was therefore further used to explore the molecular changes associated with these meat type-dependent responses.

### 3.4. Proteomics Analysis

#### 3.4.1. Overview of Proteomic Profiling and Identification of Differentially Abundant Proteins

Quantitative proteomic analysis was performed to investigate the protein-level changes associated with the differential effects of *A. tsaoko* treatment on chicken and beef quality. The majority of identified peptides in both chicken and beef samples ranged from 7 to 27 amino acids, indicating satisfactory enzymatic digestion and supporting the overall quality of the proteomic dataset (Figure S1).

**Figure 3:**
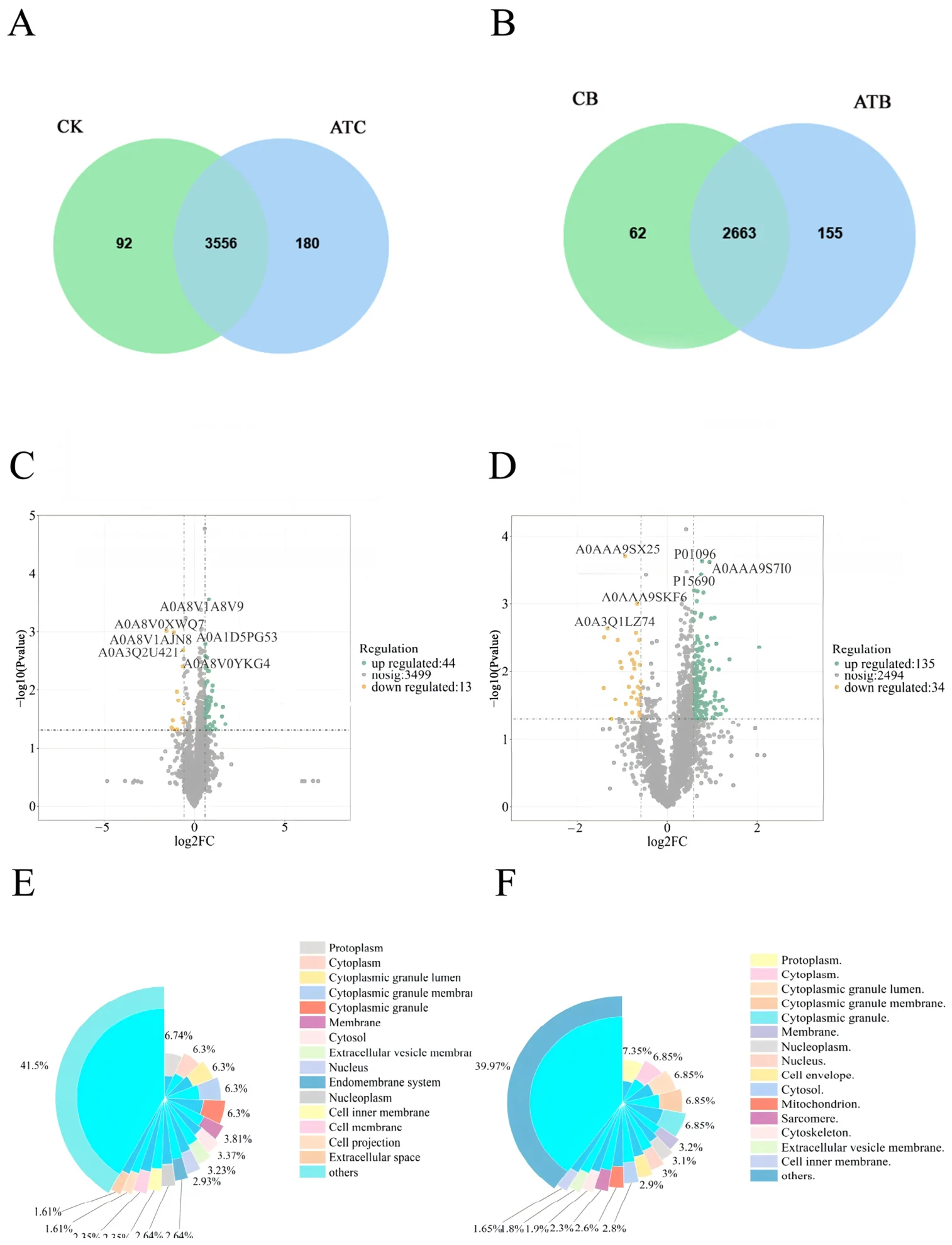
Protein identification, differential abundance, and subcellular localization analyses after *A. tsaoko* treatment. (**A**,**B**) Venn diagrams of identified proteins in chicken and beef, respectively. Green circles represent the control groups, whereas blue circles represent the A. tsaoko-treated groups; overlapping regions indicate shared proteins, and non-overlapping regions indicate group-specific proteins. (**C**,**D**) Volcano plots of differentially abundant proteins (DAPs) in chicken and beef, respectively. (**E**,**F**) Subcellular localization of DAPs in chicken and beef, respectively. CK, chicken stewed without *A. tsaoko* powder; ATC, chicken stewed with *A. tsaoko* powder; CB, beef stewed without *A. tsaoko* powder; and ATB, beef stewed with *A. tsaoko* powder.

Venn diagram analysis was performed to evaluate the overlap of identified proteins between the control and *A. tsaoko*-treated groups. In the chicken groups, 3648 and 3736 proteins were identified in CK and ATC, respectively, among which 3556 proteins were shared by the two groups, while 92 and 180 proteins were uniquely identified in CK and ATC, respectively (Figure 3A). In the beef groups, 2725 and 2818 proteins were identified in CB and ATB, respectively, with 2663 shared proteins and 62 and 155 proteins uniquely identified in CB and ATB, respectively (Figure 3B). The relatively high proportion of shared proteins indicated substantial overlap in the proteins identified across the experimental groups, providing a common basis for subsequent comparative proteomic analyses.

To evaluate protein abundance differences between the control and *A. tsaoko*-treated groups, volcano plots were generated based on fold changes and *p*-values from Student’s *t*-test. Differentially abundant proteins (DAPs) were defined as proteins with a fold change (FC) >1.5 or <0.667 and a nominal *p* < 0.05. No multiple-testing correction was applied to the differential-abundance *p*-values. Proteins with FC > 1.5 and FC < 0.667 were classified as up-regulated and down-regulated, respectively. In the volcano plots, up-regulated, down-regulated, and non-significant proteins are shown in yellow, blue, and gray, respectively. Compared with CK, 57 DAPs were identified in ATC, including 44 up-regulated and 13 down-regulated proteins (Figure 3C). Compared with CB, 169 DAPs were identified in ATB, including 135 up-regulated and 34 down-regulated proteins (Figure 3D). Overall, *A. tsaoko* treatment was associated with a larger set of proteins meeting the predefined FC and nominal *p*-value thresholds in beef than in chicken. However, the greater number of DAPs in beef should not be interpreted as evidence of a stronger biological response, because the two meat types differed in overall proteome coverage and may also differ in biological variability and statistical sensitivity. Nevertheless, the different DAP profiles indicate that *A. tsaoko* treatment was associated with distinct protein-level responses in chicken and beef, consistent with the meat type-dependent differences observed in shear force and ultrastructural appearance.

It should be noted that the quantitative proteomic analysis was based on a relatively limited number of biological replicates (*n* = 3 per group). Although DAPs were screened using predefined thresholds (FC > 1.5 or <0.667 and nominal *p* < 0.05), the absence of multiple-testing correction, together with the limited sample size, increases the possibility of false-positive findings and limits the strength of inference at the individual-protein level. Therefore, the identified DAPs should be interpreted primarily as exploratory treatment-responsive candidates rather than statistically validated treatment-responsive proteins. Subsequent functional enrichment analyses were therefore used in an exploratory manner to examine whether these candidate proteins were associated with broader biological processes that might provide context for the contrasting structural and textural responses observed in chicken and beef.

#### 3.4.2. Subcellular Localization Analysis of Differentially Abundant Proteins

Because protein function is closely associated with subcellular localization, the localization patterns of differentially abundant proteins (DAPs) were analyzed using WoLF PSORT (version 0.2) to provide further insight into their potential cellular roles. Detailed localization information for all DAPs is provided in Supplementary Table S1, and their relative distributions across different subcellular compartments are visualized in Figure 3E,F. Because these localization assignments were computationally predicted, they were interpreted as exploratory information regarding the possible cellular context of the treatment-responsive candidate proteins rather than as experimentally validated protein localizations.

In chicken, the 57 DAPs identified between CK and ATC were distributed across multiple subcellular categories (Figure 3E). The largest proportion was classified as “others” (41.5%). Among the defined localization categories, relatively high proportions of DAPs were assigned to the protoplasm, cytoplasm, cytoplasmic granule lumen, cytoplasmic granule membrane, and cytoplasmic granules, whereas smaller proportions were assigned to membrane-, nuclear-, endomembrane-, and extracellular-associated compartments. These results indicate that treatment-responsive proteins in chicken were broadly distributed across multiple subcellular compartments, with cytoplasmic and cytoplasmic granule-related locations being relatively prominent among the defined categories. This broad distribution suggests that the candidate DAPs were not restricted to a single cellular compartment and may represent changes involving multiple cellular contexts; however, subcellular localization alone does not establish the biological processes affected by treatment.

In beef, the 169 DAPs identified between CB and ATB were also distributed across multiple subcellular categories (Figure 3F), with “others” representing the largest category (39.97%). Among the defined localization categories, relatively high proportions of DAPs were assigned to the protoplasm, cytoplasm, cytoplasmic granule lumen, cytoplasmic granule membrane, and cytoplasmic granules, whereas smaller proportions were assigned to membrane-, nuclear-, mitochondrial-, cytoskeletal-, and extracellular vesicle-associated compartments. Notably, a subset of DAPs was predicted to localize to the sarcomere (2.30%), showing that some of the treatment-responsive candidate proteins were associated with muscle structural compartments. However, the presence of sarcomere-localized DAPs should not by itself be interpreted as evidence of sarcomere remodeling or disruption. Rather, these proteins provided candidates for subsequent functional enrichment analyses and for comparison with the ultrastructural differences observed by TEM.

Overall, the subcellular localization profiles showed that DAPs in both chicken and beef were broadly distributed across diverse cellular compartments. Among the defined categories, cytoplasmic and cytoplasmic granule-related localizations were relatively prominent in both species. In beef, the predicted presence of cytoskeletal- and sarcomere-associated DAPs provided additional exploratory context for the structural differences observed at the ultrastructural level. Nevertheless, because both DAP identification and subcellular localization prediction have methodological limitations, these localization patterns were used primarily to guide subsequent GO and KEGG functional analyses rather than to support independent mechanistic conclusions.

#### 3.4.3. Functional Annotation and Enrichment Analysis of Differentially Abundant Proteins

Gene Ontology (GO) enrichment analysis was performed to characterize the biological functions of DAPs in terms of biological process (BP), cellular component (CC), and molecular function (MF). Because the enrichment analysis was based on nominal (raw) *p*-values without multiple-testing correction, the results were interpreted as exploratory functional associations rather than statistically validated pathway-level changes.

**Figure 4:**
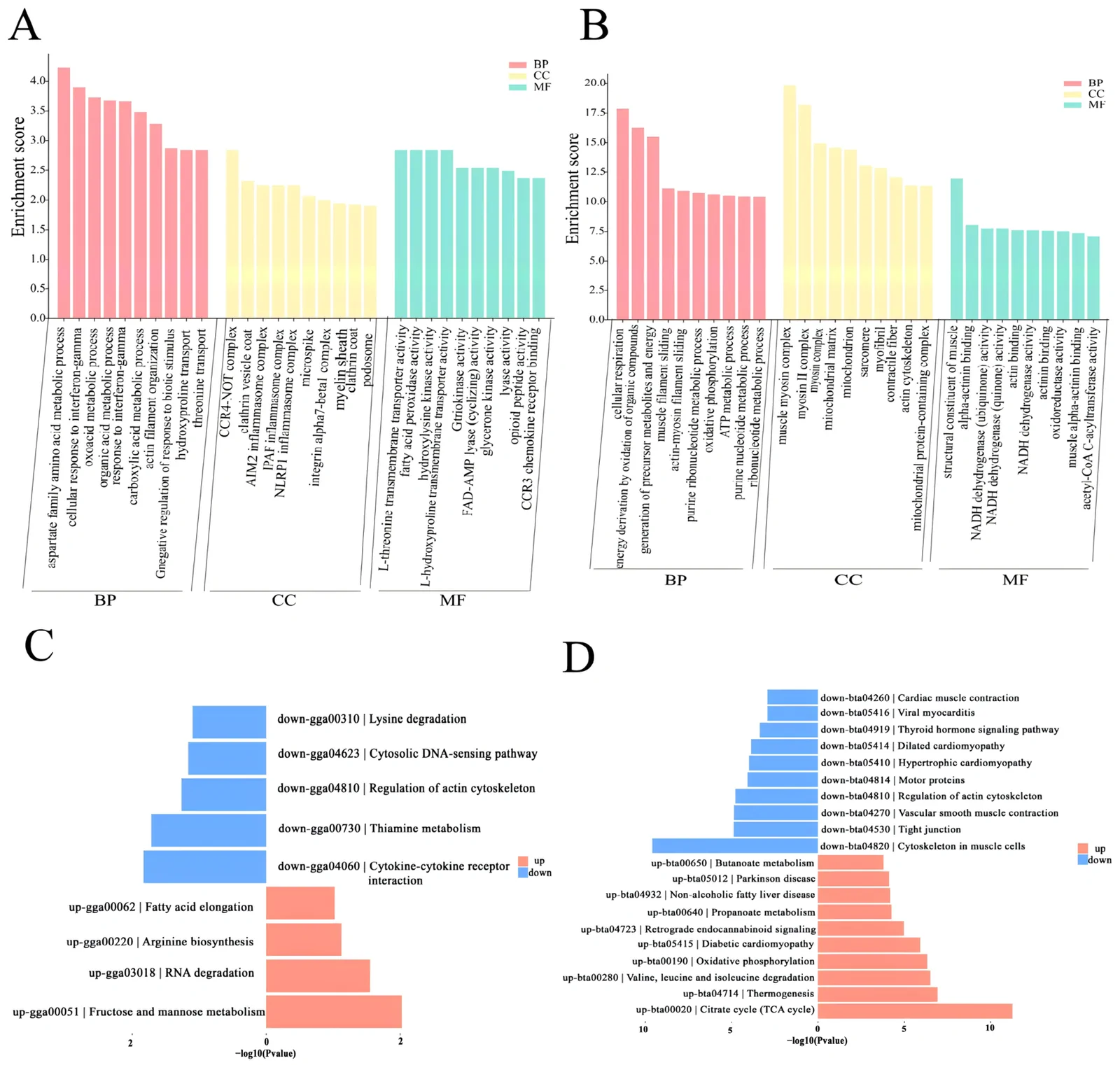
GO and KEGG enrichment analyses of differentially abundant proteins (DAPs). (**A**,**B**) GO enrichment results for chicken and beef, respectively. (**C**,**D**) KEGG enrichment results for chicken and beef, respectively. BP, biological process; CC, cellular component; MF, molecular function. Red and blue bars indicate up-regulated and down-regulated pathways or functional categories, respectively. CK, chicken stewed without *A. tsaoko* powder; ATC, chicken stewed with *A. tsaoko* powder; CB, beef stewed without *A. tsaoko* powder; and ATB, beef stewed with *A. tsaoko* powder.

In chicken, the 57 DAPs identified in ATC compared with CK were nominally enriched (*p* < 0.05) in terms primarily associated with amino acid and organic acid metabolism, cytoskeletal organization, interferon-γ-related responses, inflammasome-related components, vesicle-related components, transmembrane transport, and oxidation-related enzymatic activities (Figure 4A). Notably, actin filament organization was enriched in the BP category. Because actin and associated cytoskeletal proteins contribute to myofibrillar organization, the occurrence of these terms is consistent with the looser myofibrillar arrangement observed by TEM and may provide a possible protein-level context for the lower shear force in ATC [23]. The convergence of the TEM observations and actin-related GO terms therefore suggests that cytoskeletal organization may be one of the structural processes associated with the chicken response, although this association does not establish direct actin remodeling. Within the CC category, enriched terms included clathrin vesicle coat, clathrin coat, podosome, microspike, integrin alpha7-beta1 complex, and several inflammasome complexes, including AIM2, IPAF, and NLRP1 inflammasome complexes. These annotations suggest possible involvement of proteins related to membrane trafficking, cytoskeletal organization, and cell adhesion, although they should not be interpreted as evidence of pathway activation [34,35]. MF terms were mainly associated with amino acid transport and oxidation-related enzymatic activities.

In beef, GO enrichment analysis of the 169 DAPs identified between CB and ATB revealed nominal enrichment of terms associated with energy metabolism, mitochondrial function, muscle contraction, and myofibrillar structural components (Figure 4B). BP terms were mainly associated with cellular respiration, oxidative phosphorylation, ATP metabolic processes, muscle filament sliding, and actin–myosin filament sliding [36]. Within the CC category, enriched terms included mitochondrial matrix, mitochondrion, sarcomere, myofibril, contractile fiber, actin cytoskeleton, and myosin-related complexes. In particular, enrichment of sarcomere, myofibril, contractile fiber, and myosin-related terms indicates that myofibrillar structural proteins constituted an important component of the treatment-responsive proteome in beef. Previous studies have reported that sarcomere integrity and myofibrillar protein structure are closely associated with meat tenderness [23]. Within the MF category, enriched terms included structural constituent of muscle, alpha-actinin binding, actin binding, NADH dehydrogenase activity, oxidoreductase activity, and acetyl-CoA C-acyltransferase activity. These results further indicate that the beef DAPs were enriched in functions related to muscle structural interactions and oxidation-reduction-associated enzymatic activities. Protein oxidation has been shown to affect muscle protein structure and meat quality attributes [37]. Similarly, Song et al. [20] reported that cooking-related meat quality changes were associated with differential proteins involved in protein structure, metabolic enzymes, and oxidative stress. These observations suggest that the beef response in the present study may involve a broader combination of structural and metabolic processes.

KEGG pathway enrichment analysis further revealed different exploratory functional patterns between chicken and beef. In chicken, four pathways met the nominal *p* < 0.05 threshold: fructose and mannose metabolism and RNA degradation among up-regulated DAPs, and cytokine–cytokine receptor interaction and thiamine metabolism among down-regulated DAPs (Figure 4C). Regulation of actin cytoskeleton also showed a tendency toward enrichment (0.05 < *p* < 0.1), although it did not reach statistical significance.

In beef, the KEGG pathways shown in the enrichment plot met the nominal *p* < 0.05 threshold. Up-regulated proteins were prominently enriched in energy metabolism-related pathways, including the citrate cycle (TCA cycle) and oxidative phosphorylation (Figure 4D). In contrast, down-regulated proteins were enriched in cytoskeleton in muscle cells, regulation of actin cytoskeleton, motor proteins, and cardiac muscle contraction. These results suggest that the beef response involved candidate proteins related to both energy metabolism and muscle structural/contractile processes; however, the data do not demonstrate activation or inhibition of these pathways. The concurrent enrichment of metabolic and contractile-related pathways also provides a possible functional context for why the increase in shear force in beef could not be explained solely by the relatively modest ultrastructural differences observed by TEM.

Based on the differential protein list, subcellular localization, GO functional annotation, KEGG enrichment results, and their potential relevance to the observed texture changes, representative candidate DAPs were selected for subsequent analysis. In chicken, A0A8V1ACU4, D5LPR1, A0A8V0ZT12, and A0A8V1AL60 were selected as candidate proteins associated with cytoskeletal organization, cell adhesion, and inflammasome-related processes. In beef, A0AAA9TBS7 and P61603 were associated with mitochondrial protein folding and protein homeostasis, whereas P68138 and P60661 were associated with muscle structural and contractile functions. These proteins were selected as exploratory representatives of the major functional themes identified in the proteomic analysis rather than as validated molecular determinants of the textural changes.

#### 3.4.4. Representative DAPs for Molecular Docking Analysis

In chicken, A0A8V1ACU4, D5LPR1, A0A8V0ZT12, and A0A8V1AL60 were selected as representative DAPs. A0A8V1ACU4 (gelsolin) and D5LPR1 (fascin) were associated with actin filament organization and cytoskeletal remodeling. Gelsolin is an actin-modulating protein involved in actin filament severing and capping, whereas fascin functions as an actin-bundling protein [38,39]. A0A8V0ZT12 was related to integrin-mediated cell adhesion, and integrin α7β1 has been reported to promote muscle cell adhesion and related cellular functions [40]. A0A8V1AL60, annotated as caspase-1 in the present dataset, may be associated with inflammasome- and apoptosis-related regulation. In particular, changes in actin- and cytoskeleton-associated proteins were consistent with the enrichment of actin filament organization and may be associated with the alterations in myofibrillar organization observed by TEM and the reduced shear force of ATC. These observations are consistent with previous evidence that actin-associated proteins regulate actin filament organization [38,39], while the integrity and organization of myofibrillar and cytoskeletal structures are closely related to meat tenderness [23].

In beef, A0AAA9TBS7, P61603, P68138, and P60661 were selected as representative candidate proteins. A0AAA9TBS7 (HSP60) and P61603 (HSP10/HSPE1) are functionally related components of the mitochondrial chaperonin system involved in protein folding and protein homeostasis [41]. In contrast, P68138 (skeletal muscle α-actin) and P60661 (myosin light polypeptide 6) were associated with muscle structural organization and contraction, consistent with the enrichment ofitochondrial components, oxidative phosphorylation, the citrate cycle, sarcomere, myofibril, contractile fiber, muscle filament sliding, and actin-myosin filament sliding in the beef group. Given the close relationship between myofibrillar organization, contractile proteins, and meat tenderness, changes in these representative DAPs may be associated with the textural alterations observed in ATB following *A. tsaoko* treatment [33]. Together, the selected beef proteins reflect both mitochondrial protein-homeostasis and myofibrillar structural processes, consistent with the broader functional patterns identified by GO and KEGG analyses.

To further explore whether phenolic compounds from *A. tsaoko* could potentially interact with treatment-responsive proteins, A0A8V1ACU4 and A0A8V1AL60 from chicken and A0AAA9TBS7 and P61603 from beef were selected as representative receptor proteins for molecular docking. Candidate selection was based on differential abundance and functional relevance to the major proteomic themes rather than on a formal quantitative ranking. These proteins covered different functional features identified in the proteomic analysis, including cytoskeletal and inflammasome-related responses in chicken and mitochondrial protein-homeostasis-related responses in beef. Molecular docking was subsequently used as an exploratory analysis to characterize their potential interactions with representative phenolic compounds from *A. tsaoko*. The docking results were interpreted as complementary evidence of potential phenolic–protein interactions rather than as direct evidence that these individual interactions caused the observed changes in shear force.

### 3.5. Molecular Docking Results

#### 3.5.1. Quality Assessment of Homology Models

**Figure 5:**
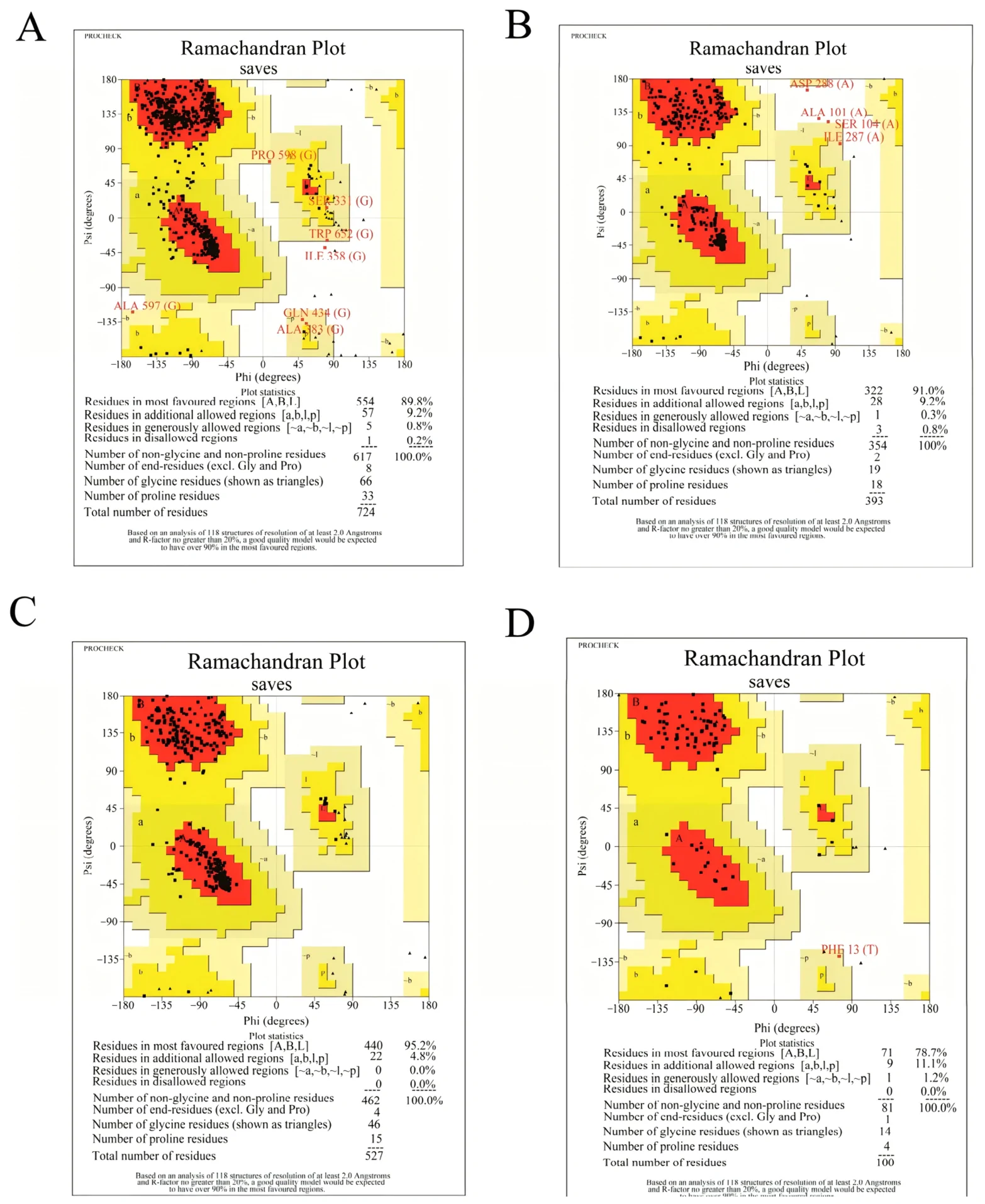
Ramachandran plots of the four protein models selected for molecular docking. (**A**) Ramachandran plot of A0A8V1ACU4 from chicken. (**B**) Ramachandran plot of A0A8V1AL60 from chicken. (**C**) Ramachandran plot of A0AAA9TBS7 from beef. (**D**) Ramachandran plot of P61603 from beef. Red, yellow, pale-yellow, and white regions represent the most favored, additionally allowed, generously allowed, and disallowed regions, respectively. Black symbols represent amino acid residues, and triangles indicate glycine residues. Residues highlighted by the PROCHECK analysis are labeled in red.

The three-dimensional structures of the four selected receptor proteins were predicted using SWISS-MODEL, and model quality was assessed by Ramachandran plot analysis using SAVES v6.0 (Figure 5). In the Ramachandran plots, residues in the most favored regions indicated highly reliable conformations, whereas residues in the additionally and generously allowed regions were considered acceptable. All models showed ≥90% of residues in favored or allowed regions, supporting their suitability for docking analysis [42].

#### 3.5.2. Analysis of Molecular Docking Energies

Molecular docking was performed to evaluate the potential interactions of caffeic acid, gallic acid, catechin, and protocatechuic acid with the selected receptor proteins from chicken and beef. All tested ligand–protein combinations showed negative predicted binding energies (Table S2), with more negative values indicating more favorable predicted ligand–receptor interactions within the docking analysis [43]. Among the four phenolic compounds, catechin showed the most favorable predicted binding energies with both chicken receptors, A0A8V1ACU4 and A0A8V1AL60, with values of −7.1 kcal/mol for each receptor. Similarly, among the four phenolic compounds tested against the beef receptors, catechin showed the most favorable predicted binding energies, with values of −9.4 kcal/mol for A0AAA9TBS7 and −6.4 kcal/mol for P61603.

In chicken, the favorable predicted interactions of catechin with A0A8V1ACU4 (gelsolin) and A0A8V1AL60 (caspase-1) suggested that catechin may potentially interact with proteins representing cytoskeletal and inflammasome-related responses, respectively. In particular, the predicted interaction with gelsolin was consistent with the proteomic enrichment of actin filament organization and provided complementary molecular information relevant to the cytoskeletal changes observed in chicken. In beef, A0AAA9TBS7 (HSP60) and P61603 (HSP10/HSPE1) are functionally associated components of the mitochondrial chaperonin system involved in protein folding and protein homeostasis [42]. Their predicted interactions with catechin therefore suggest a potential link between *A. tsaoko* phenolic compounds and proteins associated with the mitochondrial protein-homeostasis response identified in the beef proteome.

Overall, among the four phenolic compounds tested, catechin consistently exhibited the most favorable predicted binding energies with the selected receptor proteins, particularly with A0AAA9TBS7 in beef. These results provide complementary evidence for potential interactions between *A. tsaoko* phenolic compounds and treatment-responsive proteins identified by proteomic analysis.

#### 3.5.3. 3D and 2D Interaction Analysis

**Figure 6:**
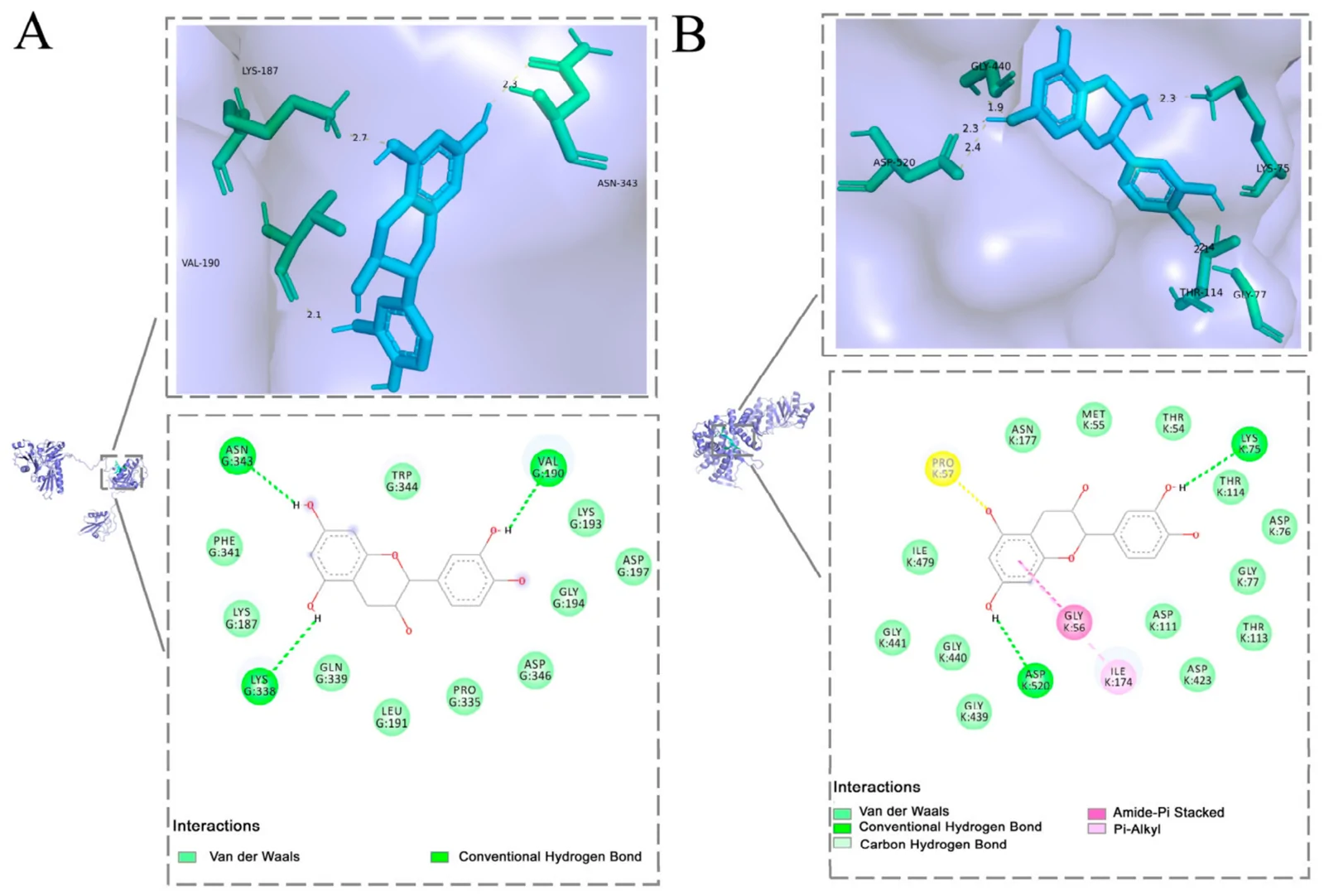
Three-dimensional (3D) binding conformations and two-dimensional (2D) interaction diagrams of representative molecular docking complexes between catechin and selected receptor proteins. (**A**) Catechin docked with A0A8V1ACU4 from chicken. (**B**) Catechin docked with A0AAA9TBS7 from beef. The 3D diagrams show the binding conformations of catechin within the corresponding receptor binding sites. In the 2D interaction diagrams, the colors and line types indicate different interaction types, as specified in the legends.

The molecular docking analysis showed differences in predicted binding energies among the four phenolic compounds and the four selected receptor proteins. Based on the predicted binding energies and the functional relevance of the receptor proteins, two representative catechin–protein complexes were selected for detailed interaction analysis (Figure 6). In chicken, catechin exhibited the same predicted binding energy (−7.1 kcal/mol) with A0A8V1ACU4 and A0A8V1AL60. A0A8V1ACU4 (gelsolin) was selected because of its functional relevance to actin filament organization and cytoskeletal regulation, which were among the functional themes emerging from the exploratory chicken proteomic analysis. In beef, the catechin–A0AAA9TBS7 complex exhibited the most favorable predicted binding energy (−9.4 kcal/mol) among all tested ligand–protein combinations. A0AAA9TBS7 (HSP60) was selected because of its association with mitochondrial protein folding and protein homeostasis, which were consistent with mitochondrial-related annotations observed in the exploratory beef proteomic analysis. The complete three-dimensional binding conformations and two-dimensional interaction diagrams for all 16 ligand–protein combinations are provided in the Supplementary Materials (Figure S2).

As shown in Figure 6A,B, catechin was located within the predicted binding pockets of A0A8V1ACU4 and A0AAA9TBS7. The 3D docking conformations showed that catechin could be accommodated in the binding cavities of the two proteins.

For the chicken group, the corresponding 2D interaction diagram showed that catechin was predicted to be stabilized within the binding pocket of A0A8V1ACU4 through multiple non-covalent interactions, mainly including conventional hydrogen bonds, van der Waals contacts, and possible hydrophobic contacts. Hydrogen-bond interactions were observed with ASN G:343, LYS G:338, and VAL G:190, which may contribute to stabilization of the predicted binding conformation. The predicted interaction between catechin and A0A8V1ACU4 provides complementary computational evidence for a potential interaction between *A. tsaoko* phenolic compounds and a cytoskeleton-associated protein.

For the beef group, the corresponding 2D interaction diagram indicated that catechin was predicted to be accommodated within the binding pocket of A0AAA9TBS7 and stabilized by multiple non-covalent interactions. Conventional hydrogen bonds were observed between catechin and LYS K:75 and ASP K:520. In addition, surrounding residues, including ASN K:177, MET K:55, THR K:54, THR K:114, ASP K:76, GLY K:77, ASP K:111, THR K:113, ASP K:423, and ILE K:479, may contribute to ligand stabilization through van der Waals contacts. Possible hydrophobic and π-related interactions involving residues such as PRO K:57, GLY K:56, and ILE K:174 may further help maintain the predicted binding conformation of catechin within the binding cavity. The predicted catechin–A0AAA9TBS7 interaction provides complementary computational evidence for a potential interaction between *A. tsaoko* phenolic compounds and a protein associated with mitochondrial protein homeostasis.

Nevertheless, molecular docking provides predictions of potential binding modes and relative binding tendencies rather than direct evidence of biological activity. The docking results should therefore be regarded as exploratory support for possible phenolic–protein interactions and interpreted together with, rather than independently from, the proteomic and ultrastructural findings. Therefore, the potential relationships among phenolic–protein interactions, proteomic changes, and meat texture require further biochemical and functional validation.

Some methodological aspects could be further refined in future studies. More detailed information on the meat samples and the batch/lot and basic composition of the *Amomum tsaoko* powder would allow more comprehensive characterization of the raw materials. Although consistent cooking procedures were applied throughout the experiment, further standardization of sample mass and cooking batches, together with direct monitoring of simmering temperature, may help improve experimental reproducibility. These considerations will be incorporated into future studies to further strengthen the robustness of the findings.

Taken together, the physicochemical, LF-NMR, ultrastructural, and exploratory proteomic results suggest a meat type-dependent response to *A. tsaoko* treatment. The contrasting shear-force responses were not accompanied by corresponding treatment-related changes in total moisture content or major water populations, whereas qualitative ultrastructural observations and proteomic analyses indicated different structural and cellular associations in chicken and beef. Molecular docking provided complementary exploratory support for potential phenolic–protein interactions, but these findings do not establish a causal mechanism.

## 4. Conclusions

Stewing with *A. tsaoko* powder exerted meat type-dependent effects on texture, decreasing shear force in chicken while increasing it in beef. These contrasting textural responses occurred without significant treatment effects on total moisture content or the relative proportions of the major T_2_ water populations, but were accompanied by qualitative differences in myofibrillar ultrastructure and distinct exploratory proteomic responses. In chicken, candidate treatment-responsive proteins were mainly associated with cytoskeletal organization, whereas in beef, the candidate proteins showed broader associations with myofibrillar organization, muscle contraction, mitochondrial function, and energy metabolism. These observations provide possible structural and protein-level context for the contrasting textural responses of chicken and beef to *A. tsaoko* treatment, but do not establish a causal relationship.

Molecular docking further showed that catechin exhibited favorable predicted interactions with the selected representative receptor proteins, particularly A0A8V1ACU4 (gelsolin) in chicken and A0AAA9TBS7 (HSP60) in beef, providing exploratory computational support for potential phenolic–protein interactions. Because the proteomic screening and enrichment analyses were based on nominal *p*-values without multiple-testing correction, and the docking results represent computational predictions, these findings should be interpreted as exploratory associations rather than validated molecular mechanisms. Further targeted biochemical and functional validation is required to confirm the roles of the identified proteins and their predicted interactions with phenolic compounds.

From a practical perspective, the present findings suggest that meat type should be considered when evaluating the potential use of *A. tsaoko* in meat processing, because its effects on texture may not be consistent across different meat systems. Future studies should investigate the dose-dependent effects of *A. tsaoko* and its major bioactive compounds using more strictly controlled meat sources and processing conditions, and further examine their interactions with muscle proteins through biochemical and functional approaches. Such studies would help further explore the biological basis of the meat type-dependent responses to *A. tsaoko* and assess its potential for targeted application in meat processing.

## Data Availability

The original contributions presented in this study are included in the article/Supplementary Materials. Further inquiries can be directed to the corresponding authors.

## Supplementary Materials

The following supporting information can be downloaded at: https://www.mdpi.com/article/10.3390/foods15203659/s1, Figure S1: Peptide length distribution in proteomic analysis; Figure S2: Three-dimensional (3D) binding conformations and two-dimensional (2D) interaction diagrams of all 16 molecular docking combinations between four representative phenolic compounds from *A. tsaoko* and four selected receptor proteins. Rows A–D correspond to A0A8V1ACU4, A0A8V1AL60, A0AAA9TBS7, and P61603, respectively. Columns 1–4 correspond to caffeic acid, gallic acid, catechin, and protocatechuic acid, respectively. Panels A3 and C3 show the representative catechin–A0A8V1ACU4 and catechin–A0AAA9TBS7 docking complexes, respectively, which are presented in enlarged form in Figure 6 of the main manuscript; Table S1: Subcellular localization annotations of differentially abundant proteins (DAPs) in chicken and beef; Table S2: Predicted binding energies of four representative phenolic compounds with the selected receptor proteins; Table S3: Template information and quality assessment parameters of receptor protein models used for molecular docking; Table S4: AutoDock Vina parameters used for blind molecular docking.

## Abbreviations

The following abbreviations are used in this manuscript:

CK	Control chicken
ATC	*Amomum tsaoko*-treated chicken
CB	Control beef
ATB	*Amomum tsaoko*-treated beef
