# Supplementary material for: Effects of Amomum tsaoko on Chicken and Beef Quality During Stewing: Insights from Microstructure, Proteomics, and Molecular Docking
Source: Foods. 2026 Oct 15;15(20):3659. doi: 10.3390/foods15203659 (PMC13704589; doi:10.3390/foods15203659)
Supplement: Supplementary file 1 [file foods-15-03659-s001.zip › foods-4565070-supplementary.pdf]

Effects of *Amomum tsaoko* on Chicken and Beef Quality during Stewing: Insights from Microstructure, Proteomics, and Molecular Docking

Siyu Zhou [1], Shikuan Zhao [1], Rui Zhou [1],Cheng Gong [2],Yuanfeng Chen [1], Huiqing Luo [1], Zezhu Du [1], Yan Shen [1], Lingrui Lu [2], Shengjie Duan [1], Jinya Dong [1], Ruijuan Yang [1], Chongye Fang [1,*] and Yunfei Ge [1,*]

[1] College of Food Science and Technology, Yunnan Agricultural University, Kunming 650201, China; zzz121300y@163.com (S.Z.); 15153216972@163.com (S.Z.); 18088435058@163.com (R.Z.); 19184056918@163.com (Y.C.); 18788107018@163.com (H.L.); pj18213743420@163.com (Z.D.); 18184494648@163.com (Y.S.); w2414510451@163.com (S.D.); dongjinyaya@163.com (J.D.); helen_4680@163.com (R.Y.)

[2] College of Agronomy and Biotechnology, Yunnan Agricultural University, Kunming 650201, China; triumph147@163.com (C.G.); 15087026316@163.com (L.L.)

* Correspondence: 2010011@ynau.edu.cn (C.F.); 18345965861@163.com (Y.G.)

Table S1. Subcellular localization annotations of differentially abundant proteins (DAPs) in chicken and beef.

Comparison	Protein	Subcellular localization
ATC vs. CK	Q90611	Extracellular space.
	Q90611	Extracellular vesicle.
	Q90611	Extracellular vesicle membrane.
	Q90611	Extracellular vesicle membrane.
	Q90611	Secreted.
	Q90611	Protoplasm.
	Q90611	Nucleus.
	Q90611	Cytoplasm.
	Q90611	Cytoplasmic granule.
	Q90611	Cytoplasmic granule lumen.
	Q90611	Cytoplasmic granule membrane.
	Q90611	Mitochondrion.
	Q90611	Cell inner membrane.
	Q90611	Cell membrane.
	Q90611	Membrane.
	Q90611	Myofibril.
	Q90611	Sarcomere.
	Q90611	Sarcomere.
	A0A8V0XC33	Protoplasm.
	A0A8V0XC33	Cytoplasm.
	A0A8V0XC33	Cytoplasmic granule.
	A0A8V0XC33	Cytoplasmic granule lumen.
	A0A8V0XC33	Cytoplasmic granule membrane.
	A0A8V0XC33	Endosome.
	A0A8V0XC33	Vacuole.
	A0A8V0XC33	Autophagosome.
	A0A8V0XC33	Centrosome.
	A0A8V0XC33	Microtubule organizing center.
	A0A8V0XC33	Cytosol.
	A0A8V0XC33	Cytoskeleton.
	A0A8V0XC33	Cell inner membrane.
	A0A8V0XC33	Cell membrane.
	A0A8V0XC33	Endosome membrane.
	A0A8V0XC33	Endomembrane system.
	A0A8V0XC33	Membrane.
	A0A8V0XC33	Vesicle.
	A0A8V0XC33	Virion.
	A0A8V0XC33	Synapse.
	A0A8V0XC33	Phagosome.
	A0A8V0XC33	Phagosome lumen.
	A0A8V1ADX0	Protoplasm.
	A0A8V1ADX0	Nucleus.
	A0A8V1ADX0	Nucleoplasm.
	A0A8V1ADX0	Nucleoplasm.
	A0A8V1ADX0	Cytoplasm.
	A0A8V1ADX0	Cytoplasmic granule.

Comparison	Protein	Subcellular localization
	A0A8V1ADX0	Cytoplasmic granule lumen.
	A0A8V1ADX0	Cytoplasmic granule membrane.
	A0A8V1ADX0	Endosome.
	A0A8V1ADX0	Microtubule organizing center.
	A0A8V1ADX0	Cytoskeleton.
	A0A8V1ADX0	Cilium.
	A0A8V1ADX0	Cilium axoneme.
	A0A8V1ADX0	Cilium basal body.
	A0A8V1ADX0	Endomembrane system.
	A0A8V1ADX0	Nuclear body.
	A0A8V1ADX0	Vesicle.
	A0A8V1ADX0	Virion.
	A0A8V1ADX0	Cell projection.
	D5LPR1	Ruffle.
	D5LPR1	Podosome.
	D5LPR1	Podosome membrane.
	D5LPR1	Protoplasm.
	D5LPR1	Cytoplasm.
	D5LPR1	Cytoplasmic granule.
	D5LPR1	Cytoplasmic granule lumen.
	D5LPR1	Cytoplasmic granule membrane.
	D5LPR1	Cytosol.
	D5LPR1	Cytoskeleton.
	D5LPR1	Cell inner membrane.
	D5LPR1	Cell membrane.
	D5LPR1	Microvillus.
	D5LPR1	Membrane.
	D5LPR1	Lamellipodium.
	D5LPR1	Filopodium.
	D5LPR1	Axon.
	D5LPR1	Dendrite.
	D5LPR1	Cell projection.
	D5LPR1	Neuron projection.
	D5LPR1	Myelin membrane.
	D5LPR1	Myelin membrane.
	E1BRV6	Protoplasm.
	E1BRV6	Centrosome.
	E1BRV6	Microtubule organizing center.
	E1BRV6	Cytoskeleton.
	E1BRV6	Cell inner membrane.
	E1BRV6	Cell membrane.
	E1BRV6	Membrane.
	E1BRV6	Dendrite.
	E1BRV6	Cell projection.
	E1BRV6	Neuron projection.
	P51641	Extracellular space.
	P51641	Extracellular vesicle.
	P51641	Extracellular vesicle membrane.
	P51641	Extracellular vesicle membrane.
	P51641	Secreted.

Comparison	Protein	Subcellular localization
	P51641	Cell inner membrane.
	P51641	Cell membrane.
	P51641	Membrane.
	P51641	Apical cell membrane.
	Q5F485	Protoplasm.
	Q5F485	Nucleus.
	Q5F485	Nucleoplasm.
	Q5F485	Nucleoplasm.
	Q5F485	Nucleolus.
	Q5F485	Cytoplasm.
	Q5F485	Cytoplasmic granule.
	Q5F485	Cytoplasmic granule lumen.
	Q5F485	Cytoplasmic granule membrane.
	Q5F485	Cytosol.
	Q5F485	Nuclear body.
	Q5F485	Nucleus speckle.
	Q5QRU7	Protoplasm.
	Q5QRU7	Nucleus.
	Q5QRU7	Nucleoplasm.
	Q5QRU7	Nucleoplasm.
	Q5QRU7	Cytoplasm.
	Q5QRU7	Cytoplasmic granule.
	Q5QRU7	Cytoplasmic granule lumen.
	Q5QRU7	Cytoplasmic granule membrane.
	Q5QRU7	Cytosol.
	Q5QRU7	Nuclear body.
	Q5QRU7	Nucleus speckle.
	Q5QRU7	Cell projection.
	Q5QRU7	Neuron projection.
	A0A1D5PG53	Protoplasm.
	A0A1D5PG53	Cytoplasm.
	A0A1D5PG53	Cytoplasmic granule.
	A0A1D5PG53	Cytoplasmic granule lumen.
	A0A1D5PG53	Cytoplasmic granule membrane.
	A0A1D5PG53	Cytosol.
	A0A3Q2TW86	Protoplasm.
	A0A3Q2TW86	Cytoplasm.
	A0A3Q2TW86	Cytoplasmic granule.
	A0A3Q2TW86	Cytoplasmic granule lumen.
	A0A3Q2TW86	Cytoplasmic granule membrane.
	A0A3Q2TW86	Cytosol.
	A0A8V0Y608	Protoplasm.
	A0A8V0Y608	Cytoplasm.
	A0A8V0Y608	Cytoplasmic granule.
	A0A8V0Y608	Cytoplasmic granule lumen.
	A0A8V0Y608	Cytoplasmic granule membrane.
	A0A8V0Y608	Endoplasmic reticulum.
	A0A8V0Y608	Endoplasmic reticulum membrane.
	A0A8V0Y608	Golgi apparatus.
	A0A8V0Y608	Endomembrane system.

Comparison	Protein	Subcellular localization
	A0A8V0Y608	Membrane.
	A0A8V0YXC4	Protoplasm.
	A0A8V0YXC4	Nucleus.
	A0A8V0YXC4	Cytoplasm.
	A0A8V0YXC4	Cytoplasmic granule.
	A0A8V0YXC4	Cytoplasmic granule lumen.
	A0A8V0YXC4	Cytoplasmic granule membrane.
	A0A8V0Z420	Extracellular space.
	A0A8V0Z420	Extracellular vesicle.
	A0A8V0Z420	Extracellular vesicle membrane.
	A0A8V0Z420	Extracellular vesicle membrane.
	A0A8V0Z420	Secreted.
	A0A8V0Z420	Protoplasm.
	A0A8V0Z420	Cytoplasm.
	A0A8V0Z420	Cytoplasmic granule.
	A0A8V0Z420	Cytoplasmic granule lumen.
	A0A8V0Z420	Cytoplasmic granule membrane.
	A0A8V0Z420	Endoplasmic reticulum.
	A0A8V0Z420	Endoplasmic reticulum membrane.
	A0A8V0Z420	Cell cortex.
	A0A8V0Z420	Endomembrane system.
	A0A8V0Z420	Membrane.
	A0A8V0Z549	Protoplasm.
	A0A8V0Z549	Cytoplasm.
	A0A8V0Z549	Cytoplasmic granule.
	A0A8V0Z549	Cytoplasmic granule lumen.
	A0A8V0Z549	Cytoplasmic granule membrane.
	A0A8V0Z549	Endosome.
	A0A8V0Z549	Vacuole.
	A0A8V0Z549	Autophagosome.
	A0A8V0Z549	Endoplasmic reticulum.
	A0A8V0Z549	Endoplasmic reticulum membrane.
	A0A8V0Z549	Golgi apparatus.
	A0A8V0Z549	Endosome membrane.
	A0A8V0Z549	Endomembrane system.
	A0A8V0Z549	Membrane.
	A0A8V0Z549	Clathrin-coated vesicle.
	A0A8V0Z549	Clathrin-coated vesicle lumen.
	A0A8V0Z549	Axon.
	A0A8V0Z549	Cytoplasmic vesicle membrane.
	A0A8V0Z549	Vesicle.
	A0A8V0Z549	Virion.
	A0A8V0Z549	Cell projection.
	A0A8V0Z549	Neuron projection.
	A0A8V0Z549	Synapse.
	A0A8V0Z549	Perinuclear region.
	A0A8V0ZE30	P-body.
	A0A8V0ZE30	Protoplasm.
	A0A8V0ZE30	Cytoplasm.
	A0A8V0ZE30	Cytoplasmic granule.

Comparison	Protein	Subcellular localization
	A0A8V0ZE30	Cytoplasmic granule lumen.
	A0A8V0ZE30	Cytoplasmic granule membrane.
	A0A8V0ZE30	Cytoplasmic ribonucleoprotein granule.
	A0A8V0ZE30	Cytoplasmic ribonucleoprotein granule.
	A0A8V0ZE30	Cytoplasmic vesicle.
	A0A8V0ZEW0	Protoplasm.
	A0A8V0ZEW0	Nucleus.
	A0A8V0ZEW0	Cytoplasm.
	A0A8V0ZEW0	Cytoplasmic granule.
	A0A8V0ZEW0	Cytoplasmic granule lumen.
	A0A8V0ZEW0	Cytoplasmic granule membrane.
	A0A8V0ZEW0	Cytosol.
	A0A8V0ZWS6	Protoplasm.
	A0A8V0ZWS6	Nucleus.
	A0A8V0ZWS6	Nucleoplasm.
	A0A8V0ZWS6	Nucleoplasm.
	A0A8V0ZWS6	Nucleolus.
	A0A8V1A184	Extracellular space.
	A0A8V1A184	Extracellular vesicle.
	A0A8V1A184	Extracellular vesicle membrane.
	A0A8V1A184	Extracellular vesicle membrane.
	A0A8V1A184	Secreted.
	A0A8V1A184	Cell inner membrane.
	A0A8V1A184	Cell membrane.
	A0A8V1A184	Membrane.
	Q5ZJ23	Lytic vacuole.
	Q5ZJ23	Protoplasm.
	Q5ZJ23	Nucleus.
	Q5ZJ23	Cytoplasm.
	Q5ZJ23	Cytoplasmic granule.
	Q5ZJ23	Cytoplasmic granule lumen.
	Q5ZJ23	Cytoplasmic granule membrane.
	Q5ZJ23	Mitochondrion.
	Q5ZJ23	Mitochondrion envelope.
	Q5ZJ23	Mitochondrion outer membrane.
	Q5ZJ23	Lysosome.
	Q5ZJ23	Vacuole.
	Q5ZJ23	Endoplasmic reticulum.
	Q5ZJ23	Cytosol.
	Q5ZJ23	Endomembrane system.
	Q5ZJ23	Membrane.
	Q5ZJ23	Mitochondrion membrane.
	Q5ZJ23	Cell envelope.
	Q5ZJ23	Cell envelope.
	Q5ZJ23	Cell projection.
	Q5ZJ23	Neuron projection.
	Q5ZJ23	Myelin membrane.
	Q5ZJ23	Myelin membrane.
	A0A1L1RM56	Protoplasm.
	A0A1L1RM56	Nucleus.

Comparison	Protein	Subcellular localization
	A0A1L1RM56	Nucleoplasm.
	A0A1L1RM56	Nucleoplasm.
	A0A1L1RM56	Cytoplasm.
	A0A1L1RM56	Cytoplasmic granule.
	A0A1L1RM56	Cytoplasmic granule lumen.
	A0A1L1RM56	Cytoplasmic granule membrane.
	A0A1L1RM56	Mitochondrion.
	A0A1L1RM56	Cytosol.
	A0A1L1RM56	Cytoskeleton.
	A0A1L1RM56	Membrane.
	A0A1L1RTK2	Protoplasm.
	A0A1L1RTK2	Nucleus.
	A0A1L1RTK2	Cytoplasm.
	A0A1L1RTK2	Cytoplasmic granule.
	A0A1L1RTK2	Cytoplasmic granule lumen.
	A0A1L1RTK2	Cytoplasmic granule membrane.
	A0A1L1RTK2	Endoplasmic reticulum.
	A0A1L1RTK2	Endoplasmic reticulum lumen.
	A0A1L1RTK2	Cytoskeleton.
	A0A1L1RTK2	Cell inner membrane.
	A0A1L1RTK2	Cell membrane.
	A0A1L1RTK2	Endomembrane system.
	A0A1L1RTK2	Membrane.
	A0A1L1RTK2	Cell envelope.
	A0A1L1RTK2	Cell envelope.
	A0A1L1RTK2	Perinuclear region.
	A0A8V0X3K2	Cell inner membrane.
	A0A8V0X3K2	Cell membrane.
	A0A8V0X3K2	Basal cell membrane.
	A0A8V0X3K2	Membrane.
	A0A8V0X3K2	Basolateral cell membrane.
	A0A8V0X3K2	Apical cell membrane.
	A0A8V0X4L7	Protoplasm.
	A0A8V0X4L7	Cytoplasm.
	A0A8V0X4L7	Cytoplasmic granule.
	A0A8V0X4L7	Cytoplasmic granule lumen.
	A0A8V0X4L7	Cytoplasmic granule membrane.
	A0A8V0X4L7	Cytosol.
	A0A8V0X4L7	Cytoskeleton.
	A0A8V0X4L7	Cell inner membrane.
	A0A8V0X4L7	Cell membrane.
	A0A8V0X4L7	Membrane.
	A0A8V0X4L7	Vesicle.
	A0A8V0X4L7	Virion.
	A0A8V0X4L7	Phagosome.
	A0A8V0X4L7	Phagosome lumen.
	A0A8V0X6I8	Extracellular space.
	A0A8V0X6I8	Extracellular vesicle.
	A0A8V0X6I8	Extracellular vesicle membrane.
	A0A8V0X6I8	Extracellular vesicle membrane.

Comparison	Protein	Subcellular localization
	A0A8V0X6I8	Secreted.
	A0A8V0X6I8	Protoplasm.
	A0A8V0X6I8	Nucleus.
	A0A8V0X6I8	Cytoplasm.
	A0A8V0X6I8	Cytoplasmic granule.
	A0A8V0X6I8	Cytoplasmic granule lumen.
	A0A8V0X6I8	Cytoplasmic granule membrane.
	A0A8V0X6I8	Mitochondrion.
	A0A8V0X6I8	Cytosol.
	A0A8V0X6I8	Cytoskeleton.
	A0A8V0X6I8	Cilium.
	A0A8V0X6I8	Cilium axoneme.
	A0A8V0X6I8	Cilium basal body.
	A0A8V0X6I8	Endomembrane system.
	A0A8V0X6I8	Membrane.
	A0A8V0X6I8	Myofibril.
	A0A8V0X6I8	Sarcomere.
	A0A8V0X6I8	Sarcomere.
	A0A8V0X6I8	Z line.
	A0A8V0X6I8	M line.
	A0A8V0X6I8	M line.
	A0A8V0X6I8	Flagellum.
	A0A8V0X6I8	Flagellum.
	A0A8V0X6I8	I band.
	A0A8V0X6I8	I band.
	A0A8V0X6I8	Vesicle.
	A0A8V0X6I8	Virion.
	A0A8V0X6I8	Cell projection.
	A0A8V0X6I8	Myelin membrane.
	A0A8V0X6I8	Myelin membrane.
	A0A8V0X6I8	Cytoplasmic vesicle lumen.
	A0A8V0X9L0	Extracellular space.
	A0A8V0X9L0	Extracellular vesicle.
	A0A8V0X9L0	Extracellular vesicle membrane.
	A0A8V0X9L0	Extracellular vesicle membrane.
	A0A8V0X9L0	Secreted.
	A0A8V0X9L0	Protoplasm.
	A0A8V0X9L0	Cytoplasm.
	A0A8V0X9L0	Cytoplasmic granule.
	A0A8V0X9L0	Cytoplasmic granule lumen.
	A0A8V0X9L0	Cytoplasmic granule membrane.
	A0A8V0X9L0	Endoplasmic reticulum.
	A0A8V0X9L0	Endoplasmic reticulum lumen.
	A0A8V0X9L0	Cytosol.
	A0A8V0X9L0	Endomembrane system.
	A0A8V0XMH2	Protoplasm.
	A0A8V0XMH2	Cytoplasm.
	A0A8V0XMH2	Cytoplasmic granule.
	A0A8V0XMH2	Cytoplasmic granule lumen.
	A0A8V0XMH2	Cytoplasmic granule membrane.

Comparison	Protein	Subcellular localization
	A0A8V0XMH2	Endosome.
	A0A8V0XMH2	Cell inner membrane.
	A0A8V0XMH2	Cell membrane.
	A0A8V0XMH2	Endomembrane system.
	A0A8V0XMH2	Membrane.
	A0A8V0XMH2	Vesicle.
	A0A8V0XMH2	Virion.
	A0A8V0Y8A2	Protoplasm.
	A0A8V0Y8A2	Cytoplasm.
	A0A8V0Y8A2	Cytoplasmic granule.
	A0A8V0Y8A2	Cytoplasmic granule lumen.
	A0A8V0Y8A2	Cytoplasmic granule membrane.
	A0A8V0Y8A2	Golgi apparatus.
	A0A8V0Y8A2	Cytosol.
	A0A8V0Y8A2	Endomembrane system.
	A0A8V0YJT6	Protoplasm.
	A0A8V0YJT6	Cytoplasm.
	A0A8V0YJT6	Cytoplasmic granule.
	A0A8V0YJT6	Cytoplasmic granule lumen.
	A0A8V0YJT6	Cytoplasmic granule membrane.
	A0A8V0YJT6	Golgi apparatus.
	A0A8V0YJT6	Endomembrane system.
	A0A8V0YKG4	Lytic vacuole.
	A0A8V0YKG4	Protoplasm.
	A0A8V0YKG4	Nucleus.
	A0A8V0YKG4	Nucleolus.
	A0A8V0YKG4	Cytoplasm.
	A0A8V0YKG4	Cytoplasmic granule.
	A0A8V0YKG4	Cytoplasmic granule lumen.
	A0A8V0YKG4	Cytoplasmic granule membrane.
	A0A8V0YKG4	Mitochondrion.
	A0A8V0YKG4	Lysosome.
	A0A8V0YKG4	Vacuole.
	A0A8V0YKG4	Cytosol.
	A0A8V0YKG4	Cell inner membrane.
	A0A8V0YKG4	Cell membrane.
	A0A8V0YKG4	Adherens junction.
	A0A8V0YKG4	Focal adhesion.
	A0A8V0YKG4	Focal adhesion.
	A0A8V0YKG4	Endomembrane system.
	A0A8V0YKG4	Membrane.
	A0A8V0YKG4	Cytoplasmic vesicle membrane.
	A0A8V0YKG4	Vesicle.
	A0A8V0YKG4	Virion.
	A0A8V0YKG4	Cell junction.
	A0A8V0YKG4	Cell junction.
	A0A8V0ZDW5	Protoplasm.
	A0A8V0ZDW5	Cytoplasm.
	A0A8V0ZDW5	Cytoplasmic granule.
	A0A8V0ZDW5	Cytoplasmic granule lumen.

Comparison	Protein	Subcellular localization
	A0A8V0ZDW5	Cytoplasmic granule membrane.
	A0A8V0ZDW5	Clathrin-coated pit.
	A0A8V0ZDW5	Endomembrane system.
	A0A8V0ZDW5	Membrane.
	A0A8V0ZDW5	Clathrin-coated vesicle.
	A0A8V0ZDW5	Clathrin-coated vesicle lumen.
	A0A8V0ZDW5	Cytoplasmic vesicle membrane.
	A0A8V0ZDW5	Clathrin-coated vesicle membrane.
	A0A8V0ZDW5	Vesicle.
	A0A8V0ZDW5	Virion.
	A0A8V0ZE98	Protoplasm.
	A0A8V0ZE98	Cytoplasm.
	A0A8V0ZE98	Cytoplasmic granule.
	A0A8V0ZE98	Cytoplasmic granule lumen.
	A0A8V0ZE98	Cytoplasmic granule membrane.
	A0A8V0ZE98	Cytosol.
	A0A8V0ZH69	Cell inner membrane.
	A0A8V0ZH69	Cell membrane.
	A0A8V0ZH69	Membrane.
	A0A8V0ZRB5	Protoplasm.
	A0A8V0ZRB5	Cytoplasm.
	A0A8V0ZRB5	Cytoplasmic granule.
	A0A8V0ZRB5	Cytoplasmic granule lumen.
	A0A8V0ZRB5	Cytoplasmic granule membrane.
	A0A8V0ZRB5	Mitochondrion.
	A0A8V0ZT12	Protoplasm.
	A0A8V0ZT12	Cytoplasm.
	A0A8V0ZT12	Cytoplasmic granule.
	A0A8V0ZT12	Cytoplasmic granule lumen.
	A0A8V0ZT12	Cytoplasmic granule membrane.
	A0A8V0ZT12	Cell inner membrane.
	A0A8V0ZT12	Cell membrane.
	A0A8V0ZT12	Adherens junction.
	A0A8V0ZT12	Focal adhesion.
	A0A8V0ZT12	Focal adhesion.
	A0A8V0ZT12	Basal cell membrane.
	A0A8V0ZT12	Membrane.
	A0A8V0ZT12	Basolateral cell membrane.
	A0A8V0ZT12	Apical cell membrane.
	A0A8V0ZT12	Synapse.
	A0A8V0ZT12	Cell junction.
	A0A8V0ZT12	Cell junction.
	A0A8V0ZU09	Protoplasm.
	A0A8V0ZU09	Nucleus.
	A0A8V0ZU09	Nucleoplasm.
	A0A8V0ZU09	Nucleoplasm.
	A0A8V0ZU09	Cytoplasm.
	A0A8V0ZU09	Cytoplasmic granule.
	A0A8V0ZU09	Cytoplasmic granule lumen.
	A0A8V0ZU09	Cytoplasmic granule membrane.

Comparison	Protein	Subcellular localization
	A0A8V0ZU09	Cytosol.
	A0A8V0ZU09	Nuclear body.
	A0A8V0ZU09	Nucleus speckle.
	A0A8V1A023	Protoplasm.
	A0A8V1A023	Cytoplasm.
	A0A8V1A023	Cytoplasmic granule.
	A0A8V1A023	Cytoplasmic granule lumen.
	A0A8V1A023	Cytoplasmic granule membrane.
	A0A8V1A023	Cytosol.
	A0A8V1A023	Clathrin-coated pit.
	A0A8V1A023	Endomembrane system.
	A0A8V1A023	Membrane.
	A0A8V1A023	Clathrin-coated vesicle.
	A0A8V1A023	Clathrin-coated vesicle lumen.
	A0A8V1A023	Cytoplasmic vesicle membrane.
	A0A8V1A023	Clathrin-coated vesicle membrane.
	A0A8V1A023	Vesicle.
	A0A8V1A023	Virion.
	A0A8V1A8S7	Extracellular space.
	A0A8V1A8S7	Extracellular vesicle.
	A0A8V1A8S7	Extracellular vesicle membrane.
	A0A8V1A8S7	Extracellular vesicle membrane.
	A0A8V1A8S7	Secreted.
	A0A8V1A8S7	Protoplasm.
	A0A8V1A8S7	Cytoplasm.
	A0A8V1A8S7	Cytoplasmic granule.
	A0A8V1A8S7	Cytoplasmic granule lumen.
	A0A8V1A8S7	Cytoplasmic granule membrane.
	A0A8V1A8S7	Myofibril.
	A0A8V1ACU4	Ruffle.
	A0A8V1ACU4	Podosome.
	A0A8V1ACU4	Podosome membrane.
	A0A8V1ACU4	Extracellular space.
	A0A8V1ACU4	Extracellular vesicle.
	A0A8V1ACU4	Extracellular vesicle membrane.
	A0A8V1ACU4	Extracellular vesicle membrane.
	A0A8V1ACU4	Secreted.
	A0A8V1ACU4	Protoplasm.
	A0A8V1ACU4	Cytoplasm.
	A0A8V1ACU4	Cytoplasmic granule.
	A0A8V1ACU4	Cytoplasmic granule lumen.
	A0A8V1ACU4	Cytoplasmic granule membrane.
	A0A8V1ACU4	Cytosol.
	A0A8V1ACU4	Cytoskeleton.
	A0A8V1ACU4	Cell inner membrane.
	A0A8V1ACU4	Cell membrane.
	A0A8V1ACU4	Microvillus.
	A0A8V1ACU4	Cell cortex.
	A0A8V1ACU4	Endomembrane system.
	A0A8V1ACU4	Membrane.

Comparison	Protein	Subcellular localization
	A0A8V1ACU4	Lamellipodium.
	A0A8V1ACU4	Filopodium.
	A0A8V1ACU4	Vesicle.
	A0A8V1ACU4	Virion.
	A0A8V1ACU4	Cell projection.
	A0A8V1ACU4	Myelin membrane.
	A0A8V1ACU4	Myelin membrane.
	A0A8V1ACU4	Phagosome.
	A0A8V1ACU4	Phagosome lumen.
	A0A8V1ACU4	Perinuclear region.
	A0A8V1ACU4	Cytoplasmic vesicle lumen.
	A0A8V1AH70	Protoplasm.
	A0A8V1AH70	Nucleus.
	A0A8V1AH70	Nucleoplasm.
	A0A8V1AH70	Nucleoplasm.
	A0A8V1AH70	Nuclear body.
	A0A8V1AJN8	Extracellular space.
	A0A8V1AJN8	Extracellular vesicle.
	A0A8V1AJN8	Extracellular vesicle membrane.
	A0A8V1AJN8	Extracellular vesicle membrane.
	A0A8V1AJN8	Secreted.
	A0A8V1AL60	Extracellular space.
	A0A8V1AL60	Extracellular vesicle.
	A0A8V1AL60	Extracellular vesicle membrane.
	A0A8V1AL60	Extracellular vesicle membrane.
	A0A8V1AL60	Secreted.
	A0A8V1AL60	Protoplasm.
	A0A8V1AL60	Nucleus.
	A0A8V1AL60	Cytoplasm.
	A0A8V1AL60	Cytoplasmic granule.
	A0A8V1AL60	Cytoplasmic granule lumen.
	A0A8V1AL60	Cytoplasmic granule membrane.
	A0A8V1AL60	Mitochondrion.
	A0A8V1AL60	Endoplasmic reticulum.
	A0A8V1AL60	Cytosol.
	A0A8V1AL60	Cell inner membrane.
	A0A8V1AL60	Cell membrane.
	A0A8V1AL60	Endomembrane system.
	A0A8V1AL60	Membrane.
	A0A8V1AL60	Cell projection.
	A0A8V1AL60	Neuron projection.
	A0A1D5P175	Cell inner membrane.
	A0A1D5P175	Cell membrane.
	A0A1D5P175	Membrane.
	A0A1D5PFA5	Protoplasm.
	A0A1D5PFA5	Cytoplasm.
	A0A1D5PFA5	Cytoplasmic granule.
	A0A1D5PFA5	Cytoplasmic granule lumen.
	A0A1D5PFA5	Cytoplasmic granule membrane.
	A0A1D5PFA5	Mitochondrion.

Comparison	Protein	Subcellular localization
	A0A1D5PFA5	Cytosol.
	A0A1D5PVS3	Protoplasm.
	A0A1D5PVS3	Cytoplasm.
	A0A1D5PVS3	Cytoplasmic granule.
	A0A1D5PVS3	Cytoplasmic granule lumen.
	A0A1D5PVS3	Cytoplasmic granule membrane.
	A0A1D5PVS3	Mitochondrion.
	A0A1D5PVS3	Mitochondrion envelope.
	A0A1D5PVS3	Mitochondrion outer membrane.
	A0A1D5PVS3	Membrane.
	A0A1D5PVS3	Mitochondrion membrane.
	A0A1D5PVS3	Cell envelope.
	A0A1D5PVS3	Cell envelope.
	A0A3Q2U421	Protoplasm.
	A0A3Q2U421	Cytoplasm.
	A0A3Q2U421	Cytoplasmic granule.
	A0A3Q2U421	Cytoplasmic granule lumen.
	A0A3Q2U421	Cytoplasmic granule membrane.
	A0A3Q2U421	Endosome.
	A0A3Q2U421	Endomembrane system.
	A0A3Q2U421	Membrane.
	A0A3Q2U421	Vesicle.
	A0A3Q2U421	Virion.
	A0A8V0XL63	Protoplasm.
	A0A8V0XL63	Nucleus.
	A0A8V0XL63	Cytoplasm.
	A0A8V0XL63	Cytoplasmic granule.
	A0A8V0XL63	Cytoplasmic granule lumen.
	A0A8V0XL63	Cytoplasmic granule membrane.
	A0A8V0XL63	Cytosol.
	A0A8V0XM35	Protoplasm.
	A0A8V0XM35	Cytoplasm.
	A0A8V0XM35	Cytoplasmic granule.
	A0A8V0XM35	Cytoplasmic granule lumen.
	A0A8V0XM35	Cytoplasmic granule membrane.
	A0A8V0XM35	Cytosol.
	A0A8V0XM35	Cell projection.
	A0A8V0XM35	Neuron projection.
	A0A8V0YHU8	P-body.
	A0A8V0YHU8	Protoplasm.
	A0A8V0YHU8	Nucleus.
	A0A8V0YHU8	Cytoplasm.
	A0A8V0YHU8	Cytoplasmic granule.
	A0A8V0YHU8	Cytoplasmic granule lumen.
	A0A8V0YHU8	Cytoplasmic granule membrane.
	A0A8V0YHU8	Cytoplasmic ribonucleoprotein granule.
	A0A8V0YHU8	Cytoplasmic ribonucleoprotein granule.
	A0A8V0YHU8	Cytoplasmic vesicle.
	A0A8V0Z335	Extracellular space.
	A0A8V0Z335	Extracellular vesicle.

Comparison	Protein	Subcellular localization
	A0A8V0Z335	Extracellular vesicle membrane.
	A0A8V0Z335	Extracellular vesicle membrane.
	A0A8V0Z335	Secreted.
	A0A8V0ZEC5	Protoplasm.
	A0A8V0ZEC5	Nucleus.
	A0A8V0ZEC5	Nucleoplasm.
	A0A8V0ZEC5	Nucleoplasm.
	A0A8V0ZEC5	Cytoplasm.
	A0A8V0ZEC5	Cytoplasmic granule.
	A0A8V0ZEC5	Cytoplasmic granule lumen.
	A0A8V0ZEC5	Cytoplasmic granule membrane.
	A0A8V0ZEC5	Cytosol.
	A0A8V0ZEC5	Synapse.
	A0A8V1A8T8	Protoplasm.
	A0A8V1A8T8	Nucleus.
	A0A8V1A8T8	Cytoplasm.
	A0A8V1A8T8	Cytoplasmic granule.
	A0A8V1A8T8	Cytoplasmic granule lumen.
	A0A8V1A8T8	Cytoplasmic granule membrane.
	A0A8V1A8T8	Myofibril.
	A0A8V1A8T8	Sarcomere.
	A0A8V1A8T8	Sarcomere.
	A0A8V1A8T8	Z line.
	A0A8V1A8T8	I band.
	A0A8V1A8T8	I band.
	A0A8V1A8V9	Protoplasm.
	A0A8V1A8V9	Cytoplasm.
	A0A8V1A8V9	Cytoplasmic granule.
	A0A8V1A8V9	Cytoplasmic granule lumen.
	A0A8V1A8V9	Cytoplasmic granule membrane.
	A0A8V1A8V9	Mitochondrion.
	A0A8V1AFW6	Protoplasm.
	A0A8V1AFW6	Nucleus.
	A0A8V1AFW6	Nucleoplasm.
	A0A8V1AFW6	Nucleoplasm.
	A0A8V1AFW6	Cytoplasm.
	A0A8V1AFW6	Cytoplasmic granule.
	A0A8V1AFW6	Cytoplasmic granule lumen.
	A0A8V1AFW6	Cytoplasmic granule membrane.
	A0A8V1AFW6	Microtubule organizing center.
	A0A8V1AFW6	Cytoskeleton.
	A0A8V1AFW6	Cilium.
	A0A8V1AFW6	Cilium axoneme.
	A0A8V1AFW6	Cilium basal body.
	A0A8V1AFW6	Cell projection.

Comparison	Protein	Subcellular localization
ATB vs. CB	A0A3Q1LYY4	Protoplasm.
	A0A3Q1LYY4	Cytoplasm.
	A0A3Q1LYY4	Cytoplasmic granule.
	A0A3Q1LYY4	Cytoplasmic granule lumen.

Comparison	Protein	Subcellular localization
	A0A3Q1LYY4	Cytoplasmic granule membrane.
	A0A3Q1LYY4	Mitochondrion.
	A0A3Q1LYY4	Mitochondrion envelope.
	A0A3Q1LYY4	Mitochondrion inner membrane.
	A0A3Q1LYY4	Peroxisome.
	A0A3Q1LYY4	Peroxisome matrix.
	A0A3Q1LYY4	Endoplasmic reticulum.
	A0A3Q1LYY4	Cytosol.
	A0A3Q1LYY4	Endomembrane system.
	A0A3Q1LYY4	Membrane.
	A0A3Q1LYY4	Mitochondrion membrane.
	A0A3Q1LYY4	Cell envelope.
	A0A3Q1LYY4	Cell envelope.
	A0A3Q1NDR1	Protoplasm.
	A0A3Q1NDR1	Cytoplasm.
	A0A3Q1NDR1	Cytoplasmic granule.
	A0A3Q1NDR1	Cytoplasmic granule lumen.
	A0A3Q1NDR1	Cytoplasmic granule membrane.
	A0A3Q1NDR1	Mitochondrion.
	A0A3Q1NDR1	Mitochondrion envelope.
	A0A3Q1NDR1	Mitochondrion inner membrane.
	A0A3Q1NDR1	Mitochondrion matrix.
	A0A3Q1NDR1	Peroxisome.
	A0A3Q1NDR1	Nucleoid.
	A0A3Q1NDR1	Membrane.
	A0A3Q1NDR1	Mitochondrion membrane.
	A0A3Q1NDR1	Cell envelope.
	A0A3Q1NDR1	Cell envelope.
	A0A3Q1NDR1	Mitochondrion nucleoid.
	A2VDK6	Ruffle.
	A2VDK6	Protoplasm.
	A2VDK6	Nucleus.
	A2VDK6	Cytoplasm.
	A2VDK6	Cytoplasmic granule.
	A2VDK6	Cytoplasmic granule lumen.
	A2VDK6	Cytoplasmic granule membrane.
	A2VDK6	Endosome.
	A2VDK6	Early endosome.
	A2VDK6	Cytosol.
	A2VDK6	Cytoskeleton.
	A2VDK6	Endomembrane system.
	A2VDK6	Lamellipodium.
	A2VDK6	Vesicle.
	A2VDK6	Virion.
	A2VDK6	Cell projection.
	A4IFH6	Protoplasm.
	A4IFH6	Cytoplasm.
	A4IFH6	Cytoplasmic granule.
	A4IFH6	Cytoplasmic granule lumen.
	A4IFH6	Cytoplasmic granule membrane.

Comparison	Protein	Subcellular localization
	A4IFH6	Endoplasmic reticulum.
	A4IFH6	Endoplasmic reticulum membrane.
	A4IFH6	Endomembrane system.
	A4IFH6	Membrane.
	A4IFH6	Sarcoplasm.
	A4IFH6	Sarcoplasmic reticulum.
	A4IFH6	Vesicle.
	A4IFH6	Virion.
	A4IFH6	Sarcoplasmic reticulum membrane.
	A4IFH6	Perinuclear region.
	A5PJS6	Protoplasm.
	A5PJS6	Nucleus.
	A5PJS6	Nucleoplasm.
	A5PJS6	Nucleoplasm.
	A5PJS6	Cytoplasm.
	A5PJS6	Cytoplasmic granule.
	A5PJS6	Cytoplasmic granule lumen.
	A5PJS6	Cytoplasmic granule membrane.
	A5PJS6	Endosome.
	A5PJS6	Early endosome.
	A5PJS6	Cytosol.
	A5PJS6	Endomembrane system.
	A5PJS6	Vesicle.
	A5PJS6	Virion.
	F1MK73	Protoplasm.
	F1MK73	Nucleus.
	F1MK73	Nucleoplasm.
	F1MK73	Nucleoplasm.
	F1MK73	Chromosome.
	F1MK73	Cytoplasm.
	F1MK73	Cytoplasmic granule.
	F1MK73	Cytoplasmic granule lumen.
	F1MK73	Cytoplasmic granule membrane.
	F1MK73	Cytosol.
	F1MK73	Membrane.
	O18778	Protoplasm.
	O18778	Cytoplasm.
	O18778	Cytoplasmic granule.
	O18778	Cytoplasmic granule lumen.
	O18778	Cytoplasmic granule membrane.
	O18778	Peroxisome.
	O18778	Peroxisome matrix.
	O18778	Cytosol.
	O46629	Protoplasm.
	O46629	Cytoplasm.
	O46629	Cytoplasmic granule.
	O46629	Cytoplasmic granule lumen.
	O46629	Cytoplasmic granule membrane.
	O46629	Mitochondrion.
	O46629	Mitochondrion envelope.

Comparison	Protein	Subcellular localization
	O46629	Mitochondrion outer membrane.
	O46629	Mitochondrion inner membrane.
	O46629	Mitochondrion matrix.
	O46629	Endoplasmic reticulum.
	O46629	Nucleoid.
	O46629	Endomembrane system.
	O46629	Membrane.
	O46629	Mitochondrion membrane.
	O46629	Cell envelope.
	O46629	Cell envelope.
	O46629	Mitochondrion nucleoid.
	P01096	Protoplasm.
	P01096	Cytoplasm.
	P01096	Cytoplasmic granule.
	P01096	Cytoplasmic granule lumen.
	P01096	Cytoplasmic granule membrane.
	P01096	Mitochondrion.
	P01096	Mitochondrion envelope.
	P01096	Mitochondrion inner membrane.
	P01096	Cell surface.
	P01096	Membrane.
	P01096	Mitochondrion membrane.
	P01096	Cell envelope.
	P01096	Cell envelope.
	P02253	Protoplasm.
	P02253	Nucleus.
	P02253	Chromosome.
	P02638	Ruffle.
	P02638	Extracellular space.
	P02638	Extracellular vesicle.
	P02638	Extracellular vesicle membrane.
	P02638	Extracellular vesicle membrane.
	P02638	Secreted.
	P02638	Protoplasm.
	P02638	Nucleus.
	P02638	Cytoplasm.
	P02638	Cytoplasmic granule.
	P02638	Cytoplasmic granule lumen.
	P02638	Cytoplasmic granule membrane.
	P02638	Cell projection.
	P02638	Perinuclear region.
	P13753	Cell inner membrane.
	P13753	Cell membrane.
	P13753	Membrane.
	P15690	Protoplasm.
	P15690	Cytoplasm.
	P15690	Cytoplasmic granule.
	P15690	Cytoplasmic granule lumen.
	P15690	Cytoplasmic granule membrane.
	P15690	Mitochondrion.

Comparison	Protein	Subcellular localization
	P15690	Mitochondrion envelope.
	P15690	Mitochondrion inner membrane.
	P15690	Mitochondrion intermembrane space.
	P15690	Mitochondrion matrix.
	P15690	Membrane.
	P15690	Mitochondrion membrane.
	P15690	Cell envelope.
	P15690	Cell envelope.
	P15690	Myelin membrane.
	P15690	Myelin membrane.
	P20004	Protoplasm.
	P20004	Cytoplasm.
	P20004	Cytoplasmic granule.
	P20004	Cytoplasmic granule lumen.
	P20004	Cytoplasmic granule membrane.
	P20004	Mitochondrion.
	P20004	Mitochondrion matrix.
	P20004	Myelin membrane.
	P20004	Myelin membrane.
	P25708	Protoplasm.
	P25708	Cytoplasm.
	P25708	Cytoplasmic granule.
	P25708	Cytoplasmic granule lumen.
	P25708	Cytoplasmic granule membrane.
	P25708	Mitochondrion.
	P25708	Mitochondrion envelope.
	P25708	Mitochondrion inner membrane.
	P25708	Cytosol.
	P25708	Membrane.
	P25708	Mitochondrion membrane.
	P25708	Cell envelope.
	P25708	Cell envelope.
	P53712	Acrosome.
	P53712	Ruffle.
	P53712	Extracellular space.
	P53712	Extracellular vesicle.
	P53712	Extracellular vesicle membrane.
	P53712	Extracellular vesicle membrane.
	P53712	Secreted.
	P53712	Basement membrane.
	P53712	Protoplasm.
	P53712	Cytoplasm.
	P53712	Cytoplasmic granule.
	P53712	Cytoplasmic granule lumen.
	P53712	Cytoplasmic granule membrane.
	P53712	Cell inner membrane.
	P53712	Cell membrane.
	P53712	Adherens junction.
	P53712	Focal adhesion.
	P53712	Focal adhesion.

Comparison	Protein	Subcellular localization
	P53712	Basal cell membrane.
	P53712	Cell surface.
	P53712	Endomembrane system.
	P53712	Membrane.
	P53712	Basolateral cell membrane.
	P53712	Lateral cell membrane.
	P53712	Myofibril.
	P53712	Sarcomere.
	P53712	Sarcomere.
	P53712	Hemidesmosome.
	P53712	Hemidesmosome.
	P53712	Filopodium.
	P53712	Dendrite.
	P53712	M line.
	P53712	M line.
	P53712	A band.
	P53712	Vesicle.
	P53712	Virion.
	P53712	Ruffle membrane.
	P53712	Sarcolemma.
	P53712	Presynaptic cell membrane.
	P53712	Cell projection.
	P53712	Neuron projection.
	P53712	Dendritic spine.
	P53712	Myelin membrane.
	P53712	Myelin membrane.
	P53712	Membrane raft.
	P53712	Synapse.
	P53712	Postsynaptic cell membrane.
	P53712	Cell junction.
	P53712	Cell junction.
	P53712	Synaptic cell membrane.
	P53712	Presynapse.
	P53712	Postsynapse.
	P62326	Protoplasm.
	P62326	Nucleus.
	P62326	Cytoplasm.
	P62326	Cytoplasmic granule.
	P62326	Cytoplasmic granule lumen.
	P62326	Cytoplasmic granule membrane.
	P62326	Cytosol.
	P62326	Cytoskeleton.
	P62326	Endomembrane system.
	P62326	Vesicle.
	P62326	Virion.
	P62326	Cytoplasmic vesicle lumen.
	P63026	Lytic vacuole.
	P63026	Protoplasm.
	P63026	Cytoplasm.
	P63026	Cytoplasmic granule.

Comparison	Protein	Subcellular localization
	P63026	Cytoplasmic granule lumen.
	P63026	Cytoplasmic granule membrane.
	P63026	Lysosome.
	P63026	Lysosome membrane.
	P63026	Vacuole.
	P63026	Vacuole membrane.
	P63026	Golgi apparatus.
	P63026	trans-Golgi network.
	P63026	Cytosol.
	P63026	Cell inner membrane.
	P63026	Cell membrane.
	P63026	Caveola.
	P63026	Synaptic vesicle.
	P63026	Endomembrane system.
	P63026	Membrane.
	P63026	Secretory vesicle.
	P63026	Secretory vesicle lumen.
	P63026	Clathrin-coated vesicle.
	P63026	Clathrin-coated vesicle lumen.
	P63026	Axon.
	P63026	Secretory vesicle membrane.
	P63026	Cytoplasmic vesicle membrane.
	P63026	Clathrin-coated vesicle membrane.
	P63026	Synaptic vesicle membrane.
	P63026	Synaptosome.
	P63026	Vesicle.
	P63026	Virion.
	P63026	Zymogen granule.
	P63026	Zymogen granule lumen.
	P63026	Zymogen granule membrane.
	P63026	Zymogen granule membrane.
	P63026	Cell projection.
	P63026	Neuron projection.
	P63026	Membrane raft.
	P63026	Synapse.
	P63026	Perinuclear region.
	P63026	Presynapse.
	Q04467	Protoplasm.
	Q04467	Cytoplasm.
	Q04467	Cytoplasmic granule.
	Q04467	Cytoplasmic granule lumen.
	Q04467	Cytoplasmic granule membrane.
	Q04467	Mitochondrion.
	Q04467	Mitochondrion envelope.
	Q04467	Mitochondrion inner membrane.
	Q04467	Mitochondrion matrix.
	Q04467	Peroxisome.
	Q04467	Cytosol.
	Q04467	Membrane.
	Q04467	Mitochondrion membrane.

Comparison	Protein	Subcellular localization
	Q04467	Cell envelope.
	Q04467	Cell envelope.
	Q0III9	Protoplasm.
	Q0III9	Cytoplasm.
	Q0III9	Cytoplasmic granule.
	Q0III9	Cytoplasmic granule lumen.
	Q0III9	Cytoplasmic granule membrane.
	Q0III9	Cytosol.
	Q0III9	Cytoskeleton.
	Q0III9	Adherens junction.
	Q0III9	Focal adhesion.
	Q0III9	Focal adhesion.
	Q0III9	Myofibril.
	Q0III9	Sarcomere.
	Q0III9	Sarcomere.
	Q0III9	Z line.
	Q0III9	Pseudopodium.
	Q0III9	I band.
	Q0III9	I band.
	Q0III9	Cell projection.
	Q0III9	Cell junction.
	Q0III9	Cell junction.
	Q28055	Protoplasm.
	Q28055	Nucleus.
	Q28055	Nucleoplasm.
	Q28055	Nucleoplasm.
	Q28055	Cytoplasm.
	Q28055	Cytoplasmic granule.
	Q28055	Cytoplasmic granule lumen.
	Q28055	Cytoplasmic granule membrane.
	Q29423	Protoplasm.
	Q29423	Nucleus.
	Q29423	Cytoplasm.
	Q29423	Cytoplasmic granule.
	Q29423	Cytoplasmic granule lumen.
	Q29423	Cytoplasmic granule membrane.
	Q29423	Golgi apparatus.
	Q29423	Cytosol.
	Q29423	Cell inner membrane.
	Q29423	Cell membrane.
	Q29423	Microvillus.
	Q29423	Cell surface.
	Q29423	Endomembrane system.
	Q29423	Membrane.
	Q29423	Basolateral cell membrane.
	Q29423	Apical cell membrane.
	Q29423	Lamellipodium.
	Q29423	Cytoplasmic vesicle membrane.
	Q29423	Lamellipodium membrane.
	Q29423	Vesicle.

Comparison	Protein	Subcellular localization
	Q29423	Virion.
	Q29423	Cell projection.
	Q2TBN3	Protoplasm.
	Q2TBN3	Nucleus.
	Q2TBN3	Nucleoplasm.
	Q2TBN3	Nucleoplasm.
	Q2TBN3	Cytoplasm.
	Q2TBN3	Cytoplasmic granule.
	Q2TBN3	Cytoplasmic granule lumen.
	Q2TBN3	Cytoplasmic granule membrane.
	Q2TBN3	Centrosome.
	Q2TBN3	Centriole.
	Q2TBN3	Microtubule organizing center.
	Q2TBN3	Cytosol.
	Q2TBN3	Cytoskeleton.
	Q2TBN3	Cilium.
	Q2TBN3	Cilium axoneme.
	Q2TBN3	Cilium basal body.
	Q2TBN3	Cell projection.
	Q2TBN3	Neuron projection.
	Q3MHW6	Protoplasm.
	Q3MHW6	Centrosome.
	Q3MHW6	Microtubule organizing center.
	Q3MHW6	Cytoskeleton.
	Q3MHW6	Cell inner membrane.
	Q3MHW6	Cell membrane.
	Q3MHW6	Membrane.
	Q3MHZ4	Podosome.
	Q3MHZ4	Podosome membrane.
	Q3MHZ4	Protoplasm.
	Q3MHZ4	Nucleus.
	Q3MHZ4	Cytoplasm.
	Q3MHZ4	Cytoplasmic granule.
	Q3MHZ4	Cytoplasmic granule lumen.
	Q3MHZ4	Cytoplasmic granule membrane.
	Q3MHZ4	Cytoskeleton.
	Q3MHZ4	Adherens junction.
	Q3MHZ4	Focal adhesion.
	Q3MHZ4	Focal adhesion.
	Q3MHZ4	Nucleus matrix.
	Q3MHZ4	Myofibril.
	Q3MHZ4	Sarcomere.
	Q3MHZ4	Sarcomere.
	Q3MHZ4	M line.
	Q3MHZ4	M line.
	Q3MHZ4	A band.
	Q3MHZ4	Cell junction.
	Q3MHZ4	Cell junction.
	Q3T0R7	Protoplasm.
	Q3T0R7	Cytoplasm.

Comparison	Protein	Subcellular localization
	Q3T0R7	Cytoplasmic granule.
	Q3T0R7	Cytoplasmic granule lumen.
	Q3T0R7	Cytoplasmic granule membrane.
	Q3T0R7	Mitochondrion.
	Q3T0R7	Mitochondrion matrix.
	Q6UC88	Cell inner membrane.
	Q6UC88	Cell membrane.
	Q6UC88	Membrane.
	A0A3Q1LJI4	Extracellular space.
	A0A3Q1LJI4	Extracellular vesicle.
	A0A3Q1LJI4	Extracellular vesicle membrane.
	A0A3Q1LJI4	Extracellular vesicle membrane.
	A0A3Q1LJI4	Secreted.
	A0A3Q1LJI4	Protoplasm.
	A0A3Q1LJI4	Nucleus.
	A0A3Q1LJI4	Nucleoplasm.
	A0A3Q1LJI4	Nucleoplasm.
	A0A3Q1LJI4	Cytoplasm.
	A0A3Q1LJI4	Cytoplasmic granule.
	A0A3Q1LJI4	Cytoplasmic granule lumen.
	A0A3Q1LJI4	Cytoplasmic granule membrane.
	A0A3Q1LJI4	Centrosome.
	A0A3Q1LJI4	Microtubule organizing center.
	A0A3Q1LJI4	Spindle.
	A0A3Q1LJI4	Cytosol.
	A0A3Q1LJI4	Cytoskeleton.
	A0A3Q1LJI4	Axon.
	A0A3Q1LJI4	Dendrite.
	A0A3Q1LJI4	Cell projection.
	A0A3Q1LJI4	Neuron projection.
	A0A3Q1LJI4	Synapse.
	A0A3Q1NK91	Protoplasm.
	A0A3Q1NK91	Cytoplasm.
	A0A3Q1NK91	Cytoplasmic granule.
	A0A3Q1NK91	Cytoplasmic granule lumen.
	A0A3Q1NK91	Cytoplasmic granule membrane.
	A0A3Q1NK91	Peroxisome.
	A0A3Q1NK91	Peroxisome matrix.
	A6QLR3	Protoplasm.
	A6QLR3	Nucleus.
	A6QLR3	Cytoplasm.
	A6QLR3	Cytoplasmic granule.
	A6QLR3	Cytoplasmic granule lumen.
	A6QLR3	Cytoplasmic granule membrane.
	A6QLR3	Cytosol.
	A7MAZ2	Preautophagosomal structure.
	A7MAZ2	Protoplasm.
	A7MAZ2	Cytoplasm.
	A7MAZ2	Cytoplasmic granule.
	A7MAZ2	Cytoplasmic granule lumen.

Comparison	Protein	Subcellular localization
	A7MAZ2	Cytoplasmic granule membrane.
	A7MAZ2	Endosome.
	A7MAZ2	Synaptic vesicle.
	A7MAZ2	Endomembrane system.
	A7MAZ2	Membrane.
	A7MAZ2	Secretory vesicle.
	A7MAZ2	Secretory vesicle lumen.
	A7MAZ2	Secretory vesicle membrane.
	A7MAZ2	Cytoplasmic vesicle membrane.
	A7MAZ2	Synaptic vesicle membrane.
	A7MAZ2	Synaptosome.
	A7MAZ2	Vesicle.
	A7MAZ2	Virion.
	A7MAZ2	Membrane raft.
	A7MAZ2	Synapse.
	A7MAZ2	Phagosome.
	A7MAZ2	Phagosome lumen.
	A7MAZ2	Recycling endosome.
	A7MAZ2	Presynapse.
	A7MAZ2	Postsynapse.
	A7MAZ2	Postsynaptic recycling endosome.
	A7MAZ2	Postsynaptic endosome.
	A7MB35	Protoplasm.
	A7MB35	Nucleus.
	A7MB35	Nucleolus.
	A7MB35	Cytoplasm.
	A7MB35	Cytoplasmic granule.
	A7MB35	Cytoplasmic granule lumen.
	A7MB35	Cytoplasmic granule membrane.
	A7MB35	Mitochondrion.
	A7MB35	Mitochondrion matrix.
	A7MB35	Myelin membrane.
	A7MB35	Myelin membrane.
	F1MVX2	Cell inner membrane.
	F1MVX2	Cell membrane.
	F1MVX2	Membrane.
	P00428	Protoplasm.
	P00428	Cytoplasm.
	P00428	Cytoplasmic granule.
	P00428	Cytoplasmic granule lumen.
	P00428	Cytoplasmic granule membrane.
	P00428	Mitochondrion.
	P00428	Mitochondrion envelope.
	P00428	Mitochondrion inner membrane.
	P00428	Membrane.
	P00428	Mitochondrion membrane.
	P00428	Cell envelope.
	P00428	Cell envelope.
	P05632	Protoplasm.
	P05632	Cytoplasm.

Comparison	Protein	Subcellular localization
	P05632	Cytoplasmic granule.
	P05632	Cytoplasmic granule lumen.
	P05632	Cytoplasmic granule membrane.
	P05632	Mitochondrion.
	P05632	Mitochondrion envelope.
	P05632	Mitochondrion inner membrane.
	P05632	Mitochondrion matrix.
	P05632	Membrane.
	P05632	Mitochondrion membrane.
	P05632	Cell envelope.
	P05632	Cell envelope.
	P11966	Protoplasm.
	P11966	Nucleus.
	P11966	Nucleoplasm.
	P11966	Nucleoplasm.
	P11966	Cytoplasm.
	P11966	Cytoplasmic granule.
	P11966	Cytoplasmic granule lumen.
	P11966	Cytoplasmic granule membrane.
	P11966	Mitochondrion.
	P11966	Mitochondrion matrix.
	P34942	Protoplasm.
	P34942	Cytoplasm.
	P34942	Cytoplasmic granule.
	P34942	Cytoplasmic granule lumen.
	P34942	Cytoplasmic granule membrane.
	P34942	Mitochondrion.
	P34942	Mitochondrion envelope.
	P34942	Mitochondrion inner membrane.
	P34942	Membrane.
	P34942	Mitochondrion membrane.
	P34942	Cell envelope.
	P34942	Cell envelope.
	P34942	Myelin membrane.
	P34942	Myelin membrane.
	P34943	Protoplasm.
	P34943	Nucleus.
	P34943	Nucleoplasm.
	P34943	Nucleoplasm.
	P34943	Cytoplasm.
	P34943	Cytoplasmic granule.
	P34943	Cytoplasmic granule lumen.
	P34943	Cytoplasmic granule membrane.
	P34943	Mitochondrion.
	P34943	Mitochondrion envelope.
	P34943	Mitochondrion inner membrane.
	P34943	Mitochondrion matrix.
	P34943	Membrane.
	P34943	Mitochondrion membrane.
	P34943	Cell envelope.

Comparison	Protein	Subcellular localization
	P34943	Cell envelope.
	P60661	Protoplasm.
	P60661	Cytoplasm.
	P60661	Cytoplasmic granule.
	P60661	Cytoplasmic granule lumen.
	P60661	Cytoplasmic granule membrane.
	P60661	Cytosol.
	P60661	Cytoskeleton.
	P82908	Protoplasm.
	P82908	Cytoplasm.
	P82908	Cytoplasmic granule.
	P82908	Cytoplasmic granule lumen.
	P82908	Cytoplasmic granule membrane.
	P82908	Mitochondrion.
	P82908	Mitochondrion envelope.
	P82908	Mitochondrion inner membrane.
	P82908	Mitochondrion matrix.
	P82908	Membrane.
	P82908	Mitochondrion membrane.
	P82908	Cell envelope.
	P82908	Cell envelope.
	Q05752	Protoplasm.
	Q05752	Cytoplasm.
	Q05752	Cytoplasmic granule.
	Q05752	Cytoplasmic granule lumen.
	Q05752	Cytoplasmic granule membrane.
	Q05752	Mitochondrion.
	Q05752	Mitochondrion envelope.
	Q05752	Mitochondrion inner membrane.
	Q05752	Mitochondrion matrix.
	Q05752	Membrane.
	Q05752	Mitochondrion membrane.
	Q05752	Cell envelope.
	Q05752	Cell envelope.
	Q148N0	Protoplasm.
	Q148N0	Nucleus.
	Q148N0	Cytoplasm.
	Q148N0	Cytoplasmic granule.
	Q148N0	Cytoplasmic granule lumen.
	Q148N0	Cytoplasmic granule membrane.
	Q148N0	Mitochondrion.
	Q148N0	Mitochondrion envelope.
	Q148N0	Mitochondrion matrix.
	Q148N0	Membrane.
	Q148N0	Mitochondrion membrane.
	Q148N0	Cell envelope.
	Q148N0	Cell envelope.
	Q1LZF8	Protoplasm.
	Q1LZF8	Nucleus.
	Q1LZF8	Cytoplasm.

Comparison	Protein	Subcellular localization
	Q1LZF8	Cytoplasmic granule.
	Q1LZF8	Cytoplasmic granule lumen.
	Q1LZF8	Cytoplasmic granule membrane.
	Q1LZF8	Endoplasmic reticulum.
	Q1LZF8	Golgi apparatus.
	Q1LZF8	Endomembrane system.
	Q28062	Lytic vacuole.
	Q28062	Extracellular space.
	Q28062	Extracellular vesicle.
	Q28062	Extracellular vesicle membrane.
	Q28062	Extracellular vesicle membrane.
	Q28062	Secreted.
	Q28062	Protoplasm.
	Q28062	Cytoplasm.
	Q28062	Cytoplasmic granule.
	Q28062	Cytoplasmic granule lumen.
	Q28062	Cytoplasmic granule membrane.
	Q28062	Lysosome.
	Q28062	Vacuole.
	Q28062	Vacuole lumen.
	Q28062	Golgi apparatus.
	Q28062	Golgi apparatus lumen.
	Q28062	Endomembrane system.
	Q28062	Membrane.
	Q28062	Dendrite.
	Q28062	Cell projection.
	Q28062	Neuron projection.
	Q28062	Lysosome lumen.
	Q28062	Synapse.
	Q29RI0	Protoplasm.
	Q29RI0	Cytoplasm.
	Q29RI0	Cytoplasmic granule.
	Q29RI0	Cytoplasmic granule lumen.
	Q29RI0	Cytoplasmic granule membrane.
	Q29RI0	Mitochondrion.
	Q29RK1	Protoplasm.
	Q29RK1	Cytoplasm.
	Q29RK1	Cytoplasmic granule.
	Q29RK1	Cytoplasmic granule lumen.
	Q29RK1	Cytoplasmic granule membrane.
	Q29RK1	Mitochondrion.
	Q29RK1	Mitochondrion matrix.
	Q29RZ0	Protoplasm.
	Q29RZ0	Cytoplasm.
	Q29RZ0	Cytoplasmic granule.
	Q29RZ0	Cytoplasmic granule lumen.
	Q29RZ0	Cytoplasmic granule membrane.
	Q29RZ0	Mitochondrion.
	Q29RZ0	Mitochondrion matrix.
	Q29RZ0	Peroxisome.

Comparison	Protein	Subcellular localization
	Q3MHL9	Protoplasm.
	Q3MHL9	Nucleus.
	Q3MHL9	Nucleoplasm.
	Q3MHL9	Nucleoplasm.
	Q3MHL9	Cytoplasm.
	Q3MHL9	Cytoplasmic granule.
	Q3MHL9	Cytoplasmic granule lumen.
	Q3MHL9	Cytoplasmic granule membrane.
	Q3MHL9	Cytosol.
	Q3ZBU7	Protoplasm.
	Q3ZBU7	Cytoplasm.
	Q3ZBU7	Cytoplasmic granule.
	Q3ZBU7	Cytoplasmic granule lumen.
	Q3ZBU7	Cytoplasmic granule membrane.
	Q3ZBU7	Cytosol.
	Q3ZBU7	Cytoskeleton.
	Q3ZBU7	Cilium.
	Q3ZBU7	Cilium axoneme.
	Q3ZBU7	Cilium basal body.
	Q3ZBU7	Axon.
	Q3ZBU7	Cell projection.
	Q3ZBU7	Neuron projection.
	Q3ZBU7	Myelin membrane.
	Q3ZBU7	Myelin membrane.
	Q58DR8	Protoplasm.
	Q58DR8	Cytoplasm.
	Q58DR8	Cytoplasmic granule.
	Q58DR8	Cytoplasmic granule lumen.
	Q58DR8	Cytoplasmic granule membrane.
	Q58DR8	Mitochondrion.
	Q58DR8	Mitochondrion matrix.
	Q58DR8	Cytosol.
	Q58DR8	Cell inner membrane.
	Q58DR8	Cell membrane.
	Q58DR8	Membrane.
	Q5EA61	Extracellular space.
	Q5EA61	Extracellular vesicle.
	Q5EA61	Extracellular vesicle membrane.
	Q5EA61	Extracellular vesicle membrane.
	Q5EA61	Secreted.
	Q5EA61	Protoplasm.
	Q5EA61	Nucleus.
	Q5EA61	Cytoplasm.
	Q5EA61	Cytoplasmic granule.
	Q5EA61	Cytoplasmic granule lumen.
	Q5EA61	Cytoplasmic granule membrane.
	Q5EA61	Mitochondrion.
	Q5EA61	Cytosol.
	Q5EA61	Dendrite.
	Q5EA61	Cell projection.

Comparison	Protein	Subcellular localization
	Q5EA61	Neuron projection.
	Q5EA61	Myelin membrane.
	Q5EA61	Myelin membrane.
	Q95323	Extracellular space.
	Q95323	Extracellular vesicle.
	Q95323	Extracellular vesicle membrane.
	Q95323	Extracellular vesicle membrane.
	Q95323	Secreted.
	Q95323	Protoplasm.
	Q95323	Cytoplasm.
	Q95323	Cytoplasmic granule.
	Q95323	Cytoplasmic granule lumen.
	Q95323	Cytoplasmic granule membrane.
	Q95323	Endoplasmic reticulum.
	Q95323	Rough endoplasmic reticulum.
	Q95323	Endoplasmic reticulum-Golgi intermediate compartment.
	Q95323	Endoplasmic reticulum-Golgi intermediate compartment lumen.
	Q95323	Golgi apparatus.
	Q95323	trans-Golgi network.
	Q95323	Cell inner membrane.
	Q95323	Cell membrane.
	Q95323	Cell surface.
	Q95323	Endomembrane system.
	Q95323	Membrane.
	Q95323	Apical cell membrane.
	Q95323	Sarcoplasm.
	Q95323	Sarcoplasmic reticulum.
	Q95323	Secretory vesicle.
	Q95323	Secretory vesicle lumen.
	Q95323	Secretory vesicle membrane.
	Q95323	Cytoplasmic vesicle membrane.
	Q95323	Vesicle.
	Q95323	Virion.
	Q95323	Sarcolemma.
	Q95323	Cell projection.
	Q95323	Perinuclear region.
	Q9BE40	Protoplasm.
	Q9BE40	Cytoplasm.
	Q9BE40	Cytoplasmic granule.
	Q9BE40	Cytoplasmic granule lumen.
	Q9BE40	Cytoplasmic granule membrane.
	Q9BE40	Cytoskeleton.
	Q9BE40	Myofibril.
	Q9BE40	Sarcomere.
	Q9BE40	Sarcomere.
	Q9BE40	A band.
	Q9BE40	Cytoplasmic ribonucleoprotein granule.
	Q9BE40	Cytoplasmic ribonucleoprotein granule.
	Q9BE40	Cytoplasmic vesicle.

Comparison	Protein	Subcellular localization
	A0A3Q1LWN6	Stress fiber.
	A0A3Q1LWN6	Protoplasm.
	A0A3Q1LWN6	Cytoplasm.
	A0A3Q1LWN6	Cytoplasmic granule.
	A0A3Q1LWN6	Cytoplasmic granule lumen.
	A0A3Q1LWN6	Cytoplasmic granule membrane.
	A0A3Q1LWN6	Cytosol.
	A0A3Q1LWN6	Cytoskeleton.
	A0A3Q1LWN6	Cell inner membrane.
	A0A3Q1LWN6	Cell membrane.
	A0A3Q1LWN6	Cell cortex.
	A0A3Q1LWN6	Membrane.
	A0A3Q1LWN6	Myofibril.
	A0A3Q1LWN6	Sarcomere.
	A0A3Q1LWN6	Sarcomere.
	A0A3Q1LWN6	Z line.
	A0A3Q1LWN6	I band.
	A0A3Q1LWN6	I band.
	A0A3Q1LWN6	Cleavage furrow.
	A0A3Q1LWN6	Coated pit.
	A0AAA9TBS7	Extracellular space.
	A0AAA9TBS7	Extracellular vesicle.
	A0AAA9TBS7	Extracellular vesicle membrane.
	A0AAA9TBS7	Extracellular vesicle membrane.
	A0AAA9TBS7	Secreted.
	A0AAA9TBS7	Protoplasm.
	A0AAA9TBS7	Cytoplasm.
	A0AAA9TBS7	Cytoplasmic granule.
	A0AAA9TBS7	Cytoplasmic granule lumen.
	A0AAA9TBS7	Cytoplasmic granule membrane.
	A0AAA9TBS7	Mitochondrion.
	A0AAA9TBS7	Mitochondrion envelope.
	A0AAA9TBS7	Mitochondrion inner membrane.
	A0AAA9TBS7	Mitochondrion matrix.
	A0AAA9TBS7	Endosome.
	A0AAA9TBS7	Early endosome.
	A0AAA9TBS7	Peroxisome.
	A0AAA9TBS7	Peroxisome matrix.
	A0AAA9TBS7	Endoplasmic reticulum.
	A0AAA9TBS7	Rough endoplasmic reticulum.
	A0AAA9TBS7	Golgi apparatus.
	A0AAA9TBS7	Cytosol.
	A0AAA9TBS7	Cell inner membrane.
	A0AAA9TBS7	Cell membrane.
	A0AAA9TBS7	Clathrin-coated pit.
	A0AAA9TBS7	Cell surface.
	A0AAA9TBS7	Endomembrane system.
	A0AAA9TBS7	Membrane.
	A0AAA9TBS7	Mitochondrion membrane.
	A0AAA9TBS7	Cell envelope.

Comparison	Protein	Subcellular localization
	A0AAA9TBS7	Cell envelope.
	A0AAA9TBS7	Vesicle.
	A0AAA9TBS7	Virion.
	A0AAA9TBS7	Zymogen granule.
	A0AAA9TBS7	Zymogen granule lumen.
	A0AAA9TBS7	Myelin membrane.
	A0AAA9TBS7	Myelin membrane.
	A0AAA9TBS7	Membrane raft.
	A0JN52	Protoplasm.
	A0JN52	Nucleus.
	A0JN52	Nucleoplasm.
	A0JN52	Nucleoplasm.
	A0JN52	Nucleolus.
	A4FUZ6	Protoplasm.
	A4FUZ6	Cytoplasm.
	A4FUZ6	Cytoplasmic granule.
	A4FUZ6	Cytoplasmic granule lumen.
	A4FUZ6	Cytoplasmic granule membrane.
	A4FUZ6	Mitochondrion.
	A4FUZ6	Peroxisome.
	A4IFB1	Protoplasm.
	A4IFB1	Nucleus.
	A4IFB1	Nucleoplasm.
	A4IFB1	Nucleoplasm.
	A4IFB1	Cytoplasm.
	A4IFB1	Cytoplasmic granule.
	A4IFB1	Cytoplasmic granule lumen.
	A4IFB1	Cytoplasmic granule membrane.
	A5PJV8	Protoplasm.
	A5PJV8	Nucleus.
	A5PJV8	Nucleoplasm.
	A5PJV8	Nucleoplasm.
	A5PJV8	Cytoplasm.
	A5PJV8	Cytoplasmic granule.
	A5PJV8	Cytoplasmic granule lumen.
	A5PJV8	Cytoplasmic granule membrane.
	A5PJV8	Centrosome.
	A5PJV8	Microtubule organizing center.
	A5PJV8	Spindle.
	A5PJV8	Cytosol.
	A5PJV8	Cytoskeleton.
	A6QPA6	Protoplasm.
	A6QPA6	Cytoplasm.
	A6QPA6	Cytoplasmic granule.
	A6QPA6	Cytoplasmic granule lumen.
	A6QPA6	Cytoplasmic granule membrane.
	A6QPA6	Cytosol.
	A6QPA6	Cytoskeleton.
	A6QPA6	Myofibril.
	A6QPA6	Sarcomere.

Comparison	Protein	Subcellular localization
	A6QPA6	Sarcomere.
	A7YWE4	Protoplasm.
	A7YWE4	Cytoplasm.
	A7YWE4	Cytoplasmic granule.
	A7YWE4	Cytoplasmic granule lumen.
	A7YWE4	Cytoplasmic granule membrane.
	A7YWE4	Mitochondrion.
	A7YWE4	Mitochondrion matrix.
	A7YWE4	Cell inner membrane.
	A7YWE4	Cell membrane.
	A7YWE4	Membrane.
	A8E4L1	Protoplasm.
	A8E4L1	Cytoplasm.
	A8E4L1	Cytoplasmic granule.
	A8E4L1	Cytoplasmic granule lumen.
	A8E4L1	Cytoplasmic granule membrane.
	A8E4L1	Mitochondrion.
	A8E4L1	Mitochondrion envelope.
	A8E4L1	Mitochondrion intermembrane space.
	A8E4L1	Cytosol.
	A8E4L1	Cell envelope.
	A8E4L1	Cell envelope.
	F1MDB2	Protoplasm.
	F1MDB2	Cytoplasm.
	F1MDB2	Cytoplasmic granule.
	F1MDB2	Cytoplasmic granule lumen.
	F1MDB2	Cytoplasmic granule membrane.
	F1MDB2	Mitochondrion.
	F1MDB2	Mitochondrion envelope.
	F1MDB2	Mitochondrion inner membrane.
	F1MDB2	Membrane.
	F1MDB2	Mitochondrion membrane.
	F1MDB2	Cell envelope.
	F1MDB2	Cell envelope.
	F1MGC0	Protoplasm.
	F1MGC0	Cytoplasm.
	F1MGC0	Cytoplasmic granule.
	F1MGC0	Cytoplasmic granule lumen.
	F1MGC0	Cytoplasmic granule membrane.
	F1MGC0	Mitochondrion.
	F1MGC0	Mitochondrion matrix.
	F1MGC0	Myelin membrane.
	F1MGC0	Myelin membrane.
	F1MLI8	Ruffle.
	F1MLI8	Extracellular space.
	F1MLI8	Extracellular vesicle.
	F1MLI8	Extracellular vesicle membrane.
	F1MLI8	Extracellular vesicle membrane.
	F1MLI8	Secreted.
	F1MLI8	Protoplasm.

Comparison	Protein	Subcellular localization
	F1MLI8	Nucleus.
	F1MLI8	Nucleolus.
	F1MLI8	Cytoplasm.
	F1MLI8	Cytoplasmic granule.
	F1MLI8	Cytoplasmic granule lumen.
	F1MLI8	Cytoplasmic granule membrane.
	F1MLI8	Microtubule organizing center.
	F1MLI8	Cytoskeleton.
	F1MLI8	Cell inner membrane.
	F1MLI8	Cell membrane.
	F1MLI8	Microvillus.
	F1MLI8	Membrane.
	F1MLI8	Apical cell membrane.
	F1MLI8	Lamellipodium.
	F1MLI8	Filopodium.
	F1MLI8	Microvillus membrane.
	F1MLI8	Vesicle.
	F1MLI8	Virion.
	F1MLI8	Cell projection.
	F1MRC2	Protoplasm.
	F1MRC2	Cytoplasm.
	F1MRC2	Cytoplasmic granule.
	F1MRC2	Cytoplasmic granule lumen.
	F1MRC2	Cytoplasmic granule membrane.
	F1MRC2	Cytosol.
	F1MRC2	Cytoskeleton.
	F1MRC2	Myofibril.
	F1MRC2	Sarcomere.
	F1MRC2	Sarcomere.
	F6RD87	Protoplasm.
	F6RD87	Nucleus.
	F6RD87	Cytoplasm.
	F6RD87	Cytoplasmic granule.
	F6RD87	Cytoplasmic granule lumen.
	F6RD87	Cytoplasmic granule membrane.
	F6RD87	Axon.
	F6RD87	Cell projection.
	F6RD87	Neuron projection.
	F6RD87	Synapse.
	F6RD87	Presynaptic active zone.
	F6RD87	Presynapse.
	P02316	Protoplasm.
	P02316	Nucleus.
	P02316	Nucleoplasm.
	P02316	Nucleoplasm.
	P02316	Chromosome.
	P02316	Cytoplasm.
	P02316	Cytoplasmic granule.
	P02316	Cytoplasmic granule lumen.
	P02316	Cytoplasmic granule membrane.

Comparison	Protein	Subcellular localization
	P23934	Protoplasm.
	P23934	Cytoplasm.
	P23934	Cytoplasmic granule.
	P23934	Cytoplasmic granule lumen.
	P23934	Cytoplasmic granule membrane.
	P23934	Mitochondrion.
	P23934	Mitochondrion envelope.
	P23934	Mitochondrion inner membrane.
	P23934	Membrane.
	P23934	Mitochondrion membrane.
	P23934	Cell envelope.
	P23934	Cell envelope.
	P25712	Protoplasm.
	P25712	Nucleus.
	P25712	Nucleoplasm.
	P25712	Nucleoplasm.
	P25712	Cytoplasm.
	P25712	Cytoplasmic granule.
	P25712	Cytoplasmic granule lumen.
	P25712	Cytoplasmic granule membrane.
	P25712	Mitochondrion.
	P25712	Mitochondrion envelope.
	P25712	Mitochondrion inner membrane.
	P25712	Membrane.
	P25712	Mitochondrion membrane.
	P25712	Cell envelope.
	P25712	Cell envelope.
	P61603	Protoplasm.
	P61603	Cytoplasm.
	P61603	Cytoplasmic granule.
	P61603	Cytoplasmic granule lumen.
	P61603	Cytoplasmic granule membrane.
	P61603	Mitochondrion.
	P61603	Mitochondrion matrix.
	P63217	Protoplasm.
	P63217	Cytoplasm.
	P63217	Cytoplasmic granule.
	P63217	Cytoplasmic granule lumen.
	P63217	Cytoplasmic granule membrane.
	P63217	Cell inner membrane.
	P63217	Cell membrane.
	P63217	Membrane.
	Q148G8	Protoplasm.
	Q148G8	Cytoplasm.
	Q148G8	Cytoplasmic granule.
	Q148G8	Cytoplasmic granule lumen.
	Q148G8	Cytoplasmic granule membrane.
	Q148G8	Mitochondrion.
	Q148G8	Mitochondrion envelope.
	Q148G8	Mitochondrion inner membrane.

Comparison	Protein	Subcellular localization
	Q148G8	Membrane.
	Q148G8	Mitochondrion membrane.
	Q148G8	Cell envelope.
	Q148G8	Cell envelope.
	Q2HJ38	Protoplasm.
	Q2HJ38	Cytoskeleton.
	Q2HJ73	Protoplasm.
	Q2HJ73	Cytoplasm.
	Q2HJ73	Cytoplasmic granule.
	Q2HJ73	Cytoplasmic granule lumen.
	Q2HJ73	Cytoplasmic granule membrane.
	Q2HJ73	Mitochondrion.
	Q2HJ73	Mitochondrion matrix.
	Q3MHE8	Protoplasm.
	Q3MHE8	Cytoplasm.
	Q3MHE8	Cytoplasmic granule.
	Q3MHE8	Cytoplasmic granule lumen.
	Q3MHE8	Cytoplasmic granule membrane.
	Q3MHE8	Endoplasmic reticulum.
	Q3MHE8	Endoplasmic reticulum membrane.
	Q3MHE8	Endomembrane system.
	Q3MHE8	Membrane.
	Q3SZ65	Protoplasm.
	Q3SZ65	Nucleus.
	Q3SZ65	Nucleolus.
	Q3SZ65	Cytoplasm.
	Q3SZ65	Cytoplasmic granule.
	Q3SZ65	Cytoplasmic granule lumen.
	Q3SZ65	Cytoplasmic granule membrane.
	Q3SZ65	Cytosol.
	Q3SZ65	Cytoplasmic ribonucleoprotein granule.
	Q3SZ65	Cytoplasmic ribonucleoprotein granule.
	Q3SZ65	Cytoplasmic vesicle.
	Q3SZ65	Perinuclear region.
	Q3T0T5	Protoplasm.
	Q3T0T5	Nucleus.
	Q3T0T5	Nucleus envelope.
	Q3T0T5	Nucleolus.
	Q3T0T5	Endomembrane system.
	Q3T0T5	Membrane.
	Q3T0T5	Nucleus membrane.
	Q3T0T5	Cell envelope.
	Q3T0T5	Cell envelope.
	Q3ZC41	Extracellular space.
	Q3ZC41	Extracellular vesicle.
	Q3ZC41	Extracellular vesicle membrane.
	Q3ZC41	Extracellular vesicle membrane.
	Q3ZC41	Secreted.
	Q3ZC41	Protoplasm.
	Q3ZC41	Cytoplasm.

Comparison	Protein	Subcellular localization
	Q3ZC41	Cytoplasmic granule.
	Q3ZC41	Cytoplasmic granule lumen.
	Q3ZC41	Cytoplasmic granule membrane.
	Q3ZC41	Peroxisome.
	Q3ZC41	Peroxisome matrix.
	Q3ZC41	Cytosol.
	Q3ZC41	Endomembrane system.
	Q3ZC41	Vesicle.
	Q3ZC41	Virion.
	Q3ZC41	Cytoplasmic vesicle lumen.
	Q3ZCH5	Extracellular space.
	Q3ZCH5	Extracellular vesicle.
	Q3ZCH5	Extracellular vesicle membrane.
	Q3ZCH5	Extracellular vesicle membrane.
	Q3ZCH5	Secreted.
	Q3ZCH5	Cell inner membrane.
	Q3ZCH5	Cell membrane.
	Q3ZCH5	Membrane.
	Q5E9V3	Protoplasm.
	Q5E9V3	Cytoplasm.
	Q5E9V3	Cytoplasmic granule.
	Q5E9V3	Cytoplasmic granule lumen.
	Q5E9V3	Cytoplasmic granule membrane.
	Q5E9V3	Cytoskeleton.
	Q5E9V3	Myofibril.
	Q5E9V3	Sarcomere.
	Q5E9V3	Sarcomere.
	Q5E9V3	Z line.
	Q5E9V3	I band.
	Q5E9V3	I band.
	Q95108	Protoplasm.
	Q95108	Nucleus.
	Q95108	Nucleolus.
	Q95108	Cytoplasm.
	Q95108	Cytoplasmic granule.
	Q95108	Cytoplasmic granule lumen.
	Q95108	Cytoplasmic granule membrane.
	Q95108	Mitochondrion.
	Q95108	Mitochondrion matrix.
	Q95108	Dendrite.
	Q95108	Cell projection.
	Q95108	Neuron projection.
	Q9TTC5	Extracellular space.
	Q9TTC5	Extracellular vesicle.
	Q9TTC5	Extracellular vesicle membrane.
	Q9TTC5	Extracellular vesicle membrane.
	Q9TTC5	Secreted.
	Q9TTC5	Cell inner membrane.
	Q9TTC5	Cell membrane.
	Q9TTC5	Membrane.

Comparison	Protein	Subcellular localization
	A0A140T869	Protoplasm.
	A0A140T869	Nucleus.
	A0A140T869	Nucleoplasm.
	A0A140T869	Nucleoplasm.
	A0A140T869	Cytoplasm.
	A0A140T869	Cytoplasmic granule.
	A0A140T869	Cytoplasmic granule lumen.
	A0A140T869	Cytoplasmic granule membrane.
	A0A3Q1LIX4	Protoplasm.
	A0A3Q1LIX4	Nucleus.
	A0A3Q1LIX4	Nucleoplasm.
	A0A3Q1LIX4	Nucleoplasm.
	A0A3Q1LIX4	Cytoplasm.
	A0A3Q1LIX4	Cytoplasmic granule.
	A0A3Q1LIX4	Cytoplasmic granule lumen.
	A0A3Q1LIX4	Cytoplasmic granule membrane.
	A0A3Q1LIX4	Mitochondrion.
	A0A3Q1LIX4	Mitochondrion matrix.
	A0A3Q1LK90	Protoplasm.
	A0A3Q1LK90	Cytoplasm.
	A0A3Q1LK90	Cytoplasmic granule.
	A0A3Q1LK90	Cytoplasmic granule lumen.
	A0A3Q1LK90	Cytoplasmic granule membrane.
	A0A3Q1LK90	Cytosol.
	A0A3Q1LN46	Protoplasm.
	A0A3Q1LN46	Nucleus.
	A0A3Q1LN46	Nucleoplasm.
	A0A3Q1LN46	Nucleoplasm.
	A0A3Q1LN46	Cytoplasm.
	A0A3Q1LN46	Cytoplasmic granule.
	A0A3Q1LN46	Cytoplasmic granule lumen.
	A0A3Q1LN46	Cytoplasmic granule membrane.
	A0A3Q1LN46	Cytosol.
	A0A3Q1LN46	Cell inner membrane.
	A0A3Q1LN46	Cell membrane.
	A0A3Q1LN46	Adherens junction.
	A0A3Q1LN46	Cell cortex.
	A0A3Q1LN46	Membrane.
	A0A3Q1LN46	Nuclear body.
	A0A3Q1LN46	Nucleus speckle.
	A0A3Q1LN46	Cell junction.
	A0A3Q1LN46	Cell junction.
	A0A3Q1LP42	Protoplasm.
	A0A3Q1LP42	Nucleus.
	A0A3Q1LP42	Nucleoplasm.
	A0A3Q1LP42	Nucleoplasm.
	A0A3Q1LP42	Cytoplasm.
	A0A3Q1LP42	Cytoplasmic granule.
	A0A3Q1LP42	Cytoplasmic granule lumen.
	A0A3Q1LP42	Cytoplasmic granule membrane.

Comparison	Protein	Subcellular localization
	A0A3Q1LP42	Cytoskeleton.
	A0A3Q1LPQ6	Stress fiber.
	A0A3Q1LPQ6	Ruffle.
	A0A3Q1LPQ6	Protoplasm.
	A0A3Q1LPQ6	Cytoplasm.
	A0A3Q1LPQ6	Cytoplasmic granule.
	A0A3Q1LPQ6	Cytoplasmic granule lumen.
	A0A3Q1LPQ6	Cytoplasmic granule membrane.
	A0A3Q1LPQ6	Cytosol.
	A0A3Q1LPQ6	Cytoskeleton.
	A0A3Q1LPQ6	Cell inner membrane.
	A0A3Q1LPQ6	Cell membrane.
	A0A3Q1LPQ6	Membrane.
	A0A3Q1LPQ6	Myofibril.
	A0A3Q1LPQ6	Sarcomere.
	A0A3Q1LPQ6	Sarcomere.
	A0A3Q1LPQ6	Bleb.
	A0A3Q1LPQ6	Ruffle membrane.
	A0A3Q1LPQ6	Cell projection.
	A0A3Q1LQJ9	Stress fiber.
	A0A3Q1LQJ9	Protoplasm.
	A0A3Q1LQJ9	Nucleus.
	A0A3Q1LQJ9	Cytoplasm.
	A0A3Q1LQJ9	Cytoplasmic granule.
	A0A3Q1LQJ9	Cytoplasmic granule lumen.
	A0A3Q1LQJ9	Cytoplasmic granule membrane.
	A0A3Q1LQJ9	Cytosol.
	A0A3Q1LQJ9	Cytoskeleton.
	A0A3Q1LQJ9	Adherens junction.
	A0A3Q1LQJ9	Focal adhesion.
	A0A3Q1LQJ9	Focal adhesion.
	A0A3Q1LQJ9	Vesicle.
	A0A3Q1LQJ9	Virion.
	A0A3Q1LQJ9	Phagosome.
	A0A3Q1LQJ9	Phagosome lumen.
	A0A3Q1LQJ9	Cell junction.
	A0A3Q1LQJ9	Cell junction.
	A0A3Q1LRQ5	Protoplasm.
	A0A3Q1LRQ5	Nucleus.
	A0A3Q1LRQ5	Cytoplasm.
	A0A3Q1LRQ5	Cytoplasmic granule.
	A0A3Q1LRQ5	Cytoplasmic granule lumen.
	A0A3Q1LRQ5	Cytoplasmic granule membrane.
	A0A3Q1LRQ5	Endosome.
	A0A3Q1LRQ5	Early endosome.
	A0A3Q1LRQ5	Late endosome.
	A0A3Q1LRQ5	Multivesicular body.
	A0A3Q1LRQ5	Multivesicular body.
	A0A3Q1LRQ5	Multivesicular body lumen.
	A0A3Q1LRQ5	Endoplasmic reticulum.

Comparison	Protein	Subcellular localization
	A0A3Q1LRQ5	Centrosome.
	A0A3Q1LRQ5	Microtubule organizing center.
	A0A3Q1LRQ5	Cytoskeleton.
	A0A3Q1LRQ5	Endomembrane system.
	A0A3Q1LRQ5	COPII-coated vesicle.
	A0A3Q1LRQ5	COPII-coated vesicle lumen.
	A0A3Q1LRQ5	Vesicle.
	A0A3Q1LRQ5	Virion.
	A0A3Q1LVX4	Protoplasm.
	A0A3Q1LVX4	Cytoplasm.
	A0A3Q1LVX4	Cytoplasmic granule.
	A0A3Q1LVX4	Cytoplasmic granule lumen.
	A0A3Q1LVX4	Cytoplasmic granule membrane.
	A0A3Q1LVX4	Mitochondrion.
	A0A3Q1LVX4	Mitochondrion matrix.
	A0A3Q1LVX4	Cytosol.
	A0A3Q1LW57	Acrosome.
	A0A3Q1LW57	Protoplasm.
	A0A3Q1LW57	Cytoplasm.
	A0A3Q1LW57	Cytoplasmic granule.
	A0A3Q1LW57	Cytoplasmic granule lumen.
	A0A3Q1LW57	Cytoplasmic granule membrane.
	A0A3Q1LW57	Golgi apparatus.
	A0A3Q1LW57	Cytosol.
	A0A3Q1LW57	Endomembrane system.
	A0A3Q1LW57	Membrane.
	A0A3Q1LW57	Vesicle.
	A0A3Q1LW57	Virion.
	A0A3Q1LZ74	Protoplasm.
	A0A3Q1LZ74	Cytoplasm.
	A0A3Q1LZ74	Cytoplasmic granule.
	A0A3Q1LZ74	Cytoplasmic granule lumen.
	A0A3Q1LZ74	Cytoplasmic granule membrane.
	A0A3Q1LZ74	Cytoskeleton.
	A0A3Q1LZ74	Myofibril.
	A0A3Q1LZ74	Sarcomere.
	A0A3Q1LZ74	Sarcomere.
	A0A3Q1M057	Extracellular space.
	A0A3Q1M057	Extracellular vesicle.
	A0A3Q1M057	Extracellular vesicle membrane.
	A0A3Q1M057	Extracellular vesicle membrane.
	A0A3Q1M057	Secreted.
	A0A3Q1M057	Protoplasm.
	A0A3Q1M057	Nucleus.
	A0A3Q1M057	Chromosome.
	A0A3Q1M057	Nucleolus.
	A0A3Q1M057	Cytoplasm.
	A0A3Q1M057	Cytoplasmic granule.
	A0A3Q1M057	Cytoplasmic granule lumen.
	A0A3Q1M057	Cytoplasmic granule membrane.

Comparison	Protein	Subcellular localization
	A0A3Q1M057	Golgi apparatus.
	A0A3Q1M057	trans-Golgi network.
	A0A3Q1M057	Cytosol.
	A0A3Q1M057	Cytoskeleton.
	A0A3Q1M057	Cell inner membrane.
	A0A3Q1M057	Cell membrane.
	A0A3Q1M057	Cell cortex.
	A0A3Q1M057	Endomembrane system.
	A0A3Q1M057	Membrane.
	A0A3Q1M057	Myofibril.
	A0A3Q1M057	Sarcomere.
	A0A3Q1M057	Sarcomere.
	A0A3Q1M057	Z line.
	A0A3Q1M057	Axon.
	A0A3Q1M057	Dendrite.
	A0A3Q1M057	Growth cone.
	A0A3Q1M057	I band.
	A0A3Q1M057	I band.
	A0A3Q1M057	Cell projection.
	A0A3Q1M057	Neuron projection.
	A0A3Q1M057	Perinuclear region.
	A0A3Q1M177	Protoplasm.
	A0A3Q1M177	Nucleus.
	A0A3Q1M4X8	Extracellular space.
	A0A3Q1M4X8	Extracellular vesicle.
	A0A3Q1M4X8	Extracellular vesicle membrane.
	A0A3Q1M4X8	Extracellular vesicle membrane.
	A0A3Q1M4X8	Secreted.
	A0A3Q1M4X8	Protoplasm.
	A0A3Q1M4X8	Cytoplasm.
	A0A3Q1M4X8	Cytoplasmic granule.
	A0A3Q1M4X8	Cytoplasmic granule lumen.
	A0A3Q1M4X8	Cytoplasmic granule membrane.
	A0A3Q1M4X8	Cell inner membrane.
	A0A3Q1M4X8	Cell membrane.
	A0A3Q1M4X8	Adherens junction.
	A0A3Q1M4X8	Cell surface.
	A0A3Q1M4X8	Membrane.
	A0A3Q1M4X8	Cell junction.
	A0A3Q1M4X8	Cell junction.
	A0A3Q1MUK3	Protoplasm.
	A0A3Q1MUK3	Nucleus.
	A0A3Q1MUK3	Nucleoplasm.
	A0A3Q1MUK3	Nucleoplasm.
	A0A3Q1MUK3	Cytoplasm.
	A0A3Q1MUK3	Cytoplasmic granule.
	A0A3Q1MUK3	Cytoplasmic granule lumen.
	A0A3Q1MUK3	Cytoplasmic granule membrane.
	A0A3Q1MUK3	Cytoskeleton.
	A0A3Q1MUK3	Cell inner membrane.

Comparison	Protein	Subcellular localization
	A0A3Q1MUK3	Cell membrane.
	A0A3Q1MUK3	Postsynaptic density.
	A0A3Q1MUK3	Membrane.
	A0A3Q1MUK3	Myofibril.
	A0A3Q1MUK3	Sarcomere.
	A0A3Q1MUK3	Sarcomere.
	A0A3Q1MUK3	Z line.
	A0A3Q1MUK3	Dendrite.
	A0A3Q1MUK3	I band.
	A0A3Q1MUK3	I band.
	A0A3Q1MUK3	Cell projection.
	A0A3Q1MUK3	Neuron projection.
	A0A3Q1MUK3	Synapse.
	A0A3Q1MUK3	Postsynapse.
	A0A3Q1NBQ9	Cell inner membrane.
	A0A3Q1NBQ9	Cell membrane.
	A0A3Q1NBQ9	Membrane.
	A0A3Q1NDE1	Protoplasm.
	A0A3Q1NDE1	Nucleus.
	A0A3Q1NDE1	Nucleoplasm.
	A0A3Q1NDE1	Nucleoplasm.
	A0A3Q1NDE1	Chromosome.
	A0A3Q1NDE1	Cytoplasm.
	A0A3Q1NDE1	Cytoplasmic granule.
	A0A3Q1NDE1	Cytoplasmic granule lumen.
	A0A3Q1NDE1	Cytoplasmic granule membrane.
	A0A3Q1NDE1	Cytosol.
	A0A3Q1NDE1	Cytoskeleton.
	A0A3Q1NDE1	Cell inner membrane.
	A0A3Q1NDE1	Cell membrane.
	A0A3Q1NDE1	Cell cortex.
	A0A3Q1NDE1	Postsynaptic density.
	A0A3Q1NDE1	Membrane.
	A0A3Q1NDE1	Synapse.
	A0A3Q1NDE1	Postsynapse.
	A0A3Q1NKC6	Protoplasm.
	A0A3Q1NKC6	Nucleus.
	A0A3Q1NKC6	Nucleoplasm.
	A0A3Q1NKC6	Nucleoplasm.
	A0A3Q1NKC6	Cytoplasm.
	A0A3Q1NKC6	Cytoplasmic granule.
	A0A3Q1NKC6	Cytoplasmic granule lumen.
	A0A3Q1NKC6	Cytoplasmic granule membrane.
	A0A3Q1NKC6	Mitochondrion.
	A0A3Q1NKC6	Cytosol.
	A0AAA9RV65	Protoplasm.
	A0AAA9RV65	Cytoplasm.
	A0AAA9RV65	Cytoplasmic granule.
	A0AAA9RV65	Cytoplasmic granule lumen.
	A0AAA9RV65	Cytoplasmic granule membrane.

Comparison	Protein	Subcellular localization
	A0AAA9RV65	Mitochondrion.
	A0AAA9RV65	Mitochondrion matrix.
	A0AAA9RVV7	Protoplasm.
	A0AAA9RVV7	Cytoplasm.
	A0AAA9RVV7	Cytoplasmic granule.
	A0AAA9RVV7	Cytoplasmic granule lumen.
	A0AAA9RVV7	Cytoplasmic granule membrane.
	A0AAA9RVV7	Cell inner membrane.
	A0AAA9RVV7	Cell membrane.
	A0AAA9RVV7	Endomembrane system.
	A0AAA9RVV7	Membrane.
	A0AAA9RVV7	Cytoplasmic vesicle membrane.
	A0AAA9RVV7	Vesicle.
	A0AAA9RVV7	Virion.
	A0AAA9RW43	Golgi apparatus membrane.
	A0AAA9RW43	Golgi outpost.
	A0AAA9RW43	Protoplasm.
	A0AAA9RW43	Nucleus.
	A0AAA9RW43	Nucleoplasm.
	A0AAA9RW43	Nucleoplasm.
	A0AAA9RW43	Cytoplasm.
	A0AAA9RW43	Cytoplasmic granule.
	A0AAA9RW43	Cytoplasmic granule lumen.
	A0AAA9RW43	Cytoplasmic granule membrane.
	A0AAA9RW43	Endosome.
	A0AAA9RW43	Early endosome.
	A0AAA9RW43	Golgi apparatus.
	A0AAA9RW43	trans-Golgi network.
	A0AAA9RW43	Cytosol.
	A0AAA9RW43	Endosome membrane.
	A0AAA9RW43	Endomembrane system.
	A0AAA9RW43	Membrane.
	A0AAA9RW43	Clathrin-coated vesicle.
	A0AAA9RW43	Clathrin-coated vesicle lumen.
	A0AAA9RW43	Early endosome membrane.
	A0AAA9RW43	Vesicle.
	A0AAA9RW43	Virion.
	A0AAA9RW43	trans-Golgi network membrane.
	A0AAA9RW43	Perinuclear region.
	A0AAA9RX90	Centromere.
	A0AAA9RX90	Spindle pole.
	A0AAA9RX90	Protoplasm.
	A0AAA9RX90	Nucleus.
	A0AAA9RX90	Nucleus envelope.
	A0AAA9RX90	Chromosome.
	A0AAA9RX90	Cytoplasm.
	A0AAA9RX90	Cytoplasmic granule.
	A0AAA9RX90	Cytoplasmic granule lumen.
	A0AAA9RX90	Cytoplasmic granule membrane.
	A0AAA9RX90	Centrosome.

Comparison	Protein	Subcellular localization
	A0AAA9RX90	Microtubule organizing center.
	A0AAA9RX90	Spindle.
	A0AAA9RX90	Cytosol.
	A0AAA9RX90	Cytoskeleton.
	A0AAA9RX90	Cell inner membrane.
	A0AAA9RX90	Cell membrane.
	A0AAA9RX90	Endomembrane system.
	A0AAA9RX90	Membrane.
	A0AAA9RX90	Axon.
	A0AAA9RX90	Dendrite.
	A0AAA9RX90	Cell envelope.
	A0AAA9RX90	Cell envelope.
	A0AAA9RX90	Cleavage furrow.
	A0AAA9RX90	Coated pit.
	A0AAA9RX90	Cell projection.
	A0AAA9RX90	Neuron projection.
	A0AAA9RX90	Synapse.
	A0AAA9RZ74	Protoplasm.
	A0AAA9RZ74	Nucleus.
	A0AAA9RZ74	Nucleoplasm.
	A0AAA9RZ74	Nucleoplasm.
	A0AAA9RZ74	Cytoplasm.
	A0AAA9RZ74	Cytoplasmic granule.
	A0AAA9RZ74	Cytoplasmic granule lumen.
	A0AAA9RZ74	Cytoplasmic granule membrane.
	A0AAA9RZ74	Nuclear body.
	A0AAA9S316	Protoplasm.
	A0AAA9S316	Cytoplasm.
	A0AAA9S316	Cytoplasmic granule.
	A0AAA9S316	Cytoplasmic granule lumen.
	A0AAA9S316	Cytoplasmic granule membrane.
	A0AAA9S316	Cytosol.
	A0AAA9S316	Cytoskeleton.
	A0AAA9S316	Myofibril.
	A0AAA9S316	Sarcomere.
	A0AAA9S316	Sarcomere.
	A0AAA9S316	Z line.
	A0AAA9S316	M line.
	A0AAA9S316	M line.
	A0AAA9S316	A band.
	A0AAA9S316	I band.
	A0AAA9S316	I band.
	A0AAA9S7I0	Protoplasm.
	A0AAA9S7I0	Cytoplasm.
	A0AAA9S7I0	Cytoplasmic granule.
	A0AAA9S7I0	Cytoplasmic granule lumen.
	A0AAA9S7I0	Cytoplasmic granule membrane.
	A0AAA9S7I0	Mitochondrion.
	A0AAA9S7I0	Mitochondrion matrix.
	A0AAA9SDI5	Protoplasm.

Comparison	Protein	Subcellular localization
	A0AAA9SDI5	Nucleus.
	A0AAA9SDI5	Nucleoplasm.
	A0AAA9SDI5	Nucleoplasm.
	A0AAA9SDI5	Cytoplasm.
	A0AAA9SDI5	Cytoplasmic granule.
	A0AAA9SDI5	Cytoplasmic granule lumen.
	A0AAA9SDI5	Cytoplasmic granule membrane.
	A0AAA9SDI5	Mitochondrion.
	A0AAA9SDI5	Mitochondrion matrix.
	A0AAA9SEM2	Protoplasm.
	A0AAA9SEM2	Cytoplasm.
	A0AAA9SEM2	Cytoplasmic granule.
	A0AAA9SEM2	Cytoplasmic granule lumen.
	A0AAA9SEM2	Cytoplasmic granule membrane.
	A0AAA9SEM2	Cytoskeleton.
	A0AAA9SEM2	Myofibril.
	A0AAA9SJK4	Protoplasm.
	A0AAA9SJK4	Cytoplasm.
	A0AAA9SJK4	Cytoplasmic granule.
	A0AAA9SJK4	Cytoplasmic granule lumen.
	A0AAA9SJK4	Cytoplasmic granule membrane.
	A0AAA9SJK4	Mitochondrion.
	A0AAA9SJK4	Mitochondrion matrix.
	A0AAA9SJK4	Cytosol.
	A0AAA9SLM4	Phagocytic cup.
	A0AAA9SLM4	Protoplasm.
	A0AAA9SLM4	Cytoplasm.
	A0AAA9SLM4	Cytoplasmic granule.
	A0AAA9SLM4	Cytoplasmic granule lumen.
	A0AAA9SLM4	Cytoplasmic granule membrane.
	A0AAA9SLM4	Cell inner membrane.
	A0AAA9SLM4	Cell membrane.
	A0AAA9SLM4	Adherens junction.
	A0AAA9SLM4	Focal adhesion.
	A0AAA9SLM4	Focal adhesion.
	A0AAA9SLM4	Basal cell membrane.
	A0AAA9SLM4	Cell surface.
	A0AAA9SLM4	Membrane.
	A0AAA9SLM4	Basolateral cell membrane.
	A0AAA9SLM4	Apical cell membrane.
	A0AAA9SLM4	Lateral cell membrane.
	A0AAA9SLM4	Sarcolemma.
	A0AAA9SLM4	Synapse.
	A0AAA9SLM4	Cell junction.
	A0AAA9SLM4	Cell junction.
	A0AAA9SX25	Protoplasm.
	A0AAA9SX25	Cytoplasm.
	A0AAA9SX25	Cytoplasmic granule.
	A0AAA9SX25	Cytoplasmic granule lumen.
	A0AAA9SX25	Cytoplasmic granule membrane.

Comparison	Protein	Subcellular localization
	A0AAA9SX25	Cytosol.
	A0AAA9SZL7	Protoplasm.
	A0AAA9SZL7	Cytoplasm.
	A0AAA9SZL7	Cytoplasmic granule.
	A0AAA9SZL7	Cytoplasmic granule lumen.
	A0AAA9SZL7	Cytoplasmic granule membrane.
	A0AAA9SZL7	Cytoskeleton.
	A0AAA9SZL7	Myofibril.
	A0AAA9SZL7	Sarcomere.
	A0AAA9SZL7	Sarcomere.
	A0AAA9SZL7	Z line.
	A0AAA9SZL7	M line.
	A0AAA9SZL7	M line.
	A0AAA9SZL7	A band.
	A0AAA9SZL7	I band.
	A0AAA9SZL7	I band.
	A0AAA9T2U2	Extracellular space.
	A0AAA9T2U2	Extracellular vesicle.
	A0AAA9T2U2	Extracellular vesicle membrane.
	A0AAA9T2U2	Extracellular vesicle membrane.
	A0AAA9T2U2	Secreted.
	A0AAA9T2U2	Protoplasm.
	A0AAA9T2U2	Nucleus.
	A0AAA9T2U2	Chromosome.
	A0AAA9T2U2	Cytoplasm.
	A0AAA9T2U2	Cytoplasmic granule.
	A0AAA9T2U2	Cytoplasmic granule lumen.
	A0AAA9T2U2	Cytoplasmic granule membrane.
	A0AAA9T2U2	Cytosol.
	A0AAA9T2U2	Cytoskeleton.
	A0AAA9T2U2	Myofibril.
	A0AAA9T2U2	Sarcomere.
	A0AAA9T2U2	Sarcomere.
	A0AAA9T2U2	Z line.
	A0AAA9T2U2	M line.
	A0AAA9T2U2	M line.
	A0AAA9T2U2	A band.
	A0AAA9T2U2	I band.
	A0AAA9T2U2	I band.
	A0AAA9TZ37	Extracellular space.
	A0AAA9TZ37	Extracellular vesicle.
	A0AAA9TZ37	Extracellular vesicle membrane.
	A0AAA9TZ37	Extracellular vesicle membrane.
	A0AAA9TZ37	Secreted.
	A0AAA9TZ37	Protoplasm.
	A0AAA9TZ37	Cytoplasm.
	A0AAA9TZ37	Cytoplasmic granule.
	A0AAA9TZ37	Cytoplasmic granule lumen.
	A0AAA9TZ37	Cytoplasmic granule membrane.
	A0AAA9TZ37	Endomembrane system.

Comparison	Protein	Subcellular localization
	A0AAA9TZ37	Vesicle.
	A0AAA9TZ37	Virion.
	A0AAA9TZ37	Cytoplasmic vesicle lumen.
	A6H7E3	Protoplasm.
	A7MBI3	Protoplasm.
	A7MBI3	Cytoplasm.
	A7MBI3	Cytoplasmic granule.
	A7MBI3	Cytoplasmic granule lumen.
	A7MBI3	Cytoplasmic granule membrane.
	A7MBI3	Mitochondrion.
	A7MBI3	Mitochondrion envelope.
	A7MBI3	Mitochondrion inner membrane.
	A7MBI3	Membrane.
	A7MBI3	Mitochondrion membrane.
	A7MBI3	Cell envelope.
	A7MBI3	Cell envelope.
	E1B9I1	Protoplasm.
	E1B9I1	Nucleus.
	E1B9I1	Nucleoplasm.
	E1B9I1	Nucleoplasm.
	E1B9I1	Cytoplasm.
	E1B9I1	Cytoplasmic granule.
	E1B9I1	Cytoplasmic granule lumen.
	E1B9I1	Cytoplasmic granule membrane.
	E1B9I1	Mitochondrion.
	E1B9I1	Mitochondrion envelope.
	E1B9I1	Mitochondrion inner membrane.
	E1B9I1	Membrane.
	E1B9I1	Mitochondrion membrane.
	E1B9I1	Cell envelope.
	E1B9I1	Cell envelope.
	E1BHQ9	Extracellular space.
	E1BHQ9	Extracellular vesicle.
	E1BHQ9	Extracellular vesicle membrane.
	E1BHQ9	Extracellular vesicle membrane.
	E1BHQ9	Secreted.
	E1BHQ9	Protoplasm.
	E1BHQ9	Nucleus.
	E1BHQ9	Cell inner membrane.
	E1BHQ9	Cell membrane.
	E1BHQ9	Cell surface.
	E1BHQ9	Membrane.
	E1BNV1	Protoplasm.
	E1BNV1	Cytoplasm.
	E1BNV1	Cytoplasmic granule.
	E1BNV1	Cytoplasmic granule lumen.
	E1BNV1	Cytoplasmic granule membrane.
	E1BNV1	Cytosol.
	E1BNV1	Cytoskeleton.
	E1BNV1	Myofibril.

Comparison	Protein	Subcellular localization
	E1BNV1	Sarcomere.
	E1BNV1	Sarcomere.
	E1BNV1	Z line.
	E1BNV1	M line.
	E1BNV1	M line.
	E1BNV1	A band.
	E1BNV1	I band.
	E1BNV1	I band.
	F1MJ28	Protoplasm.
	F1MJ28	Cytoplasm.
	F1MJ28	Cytoplasmic granule.
	F1MJ28	Cytoplasmic granule lumen.
	F1MJ28	Cytoplasmic granule membrane.
	F1MJ28	Endoplasmic reticulum.
	F1MJ28	Cytosol.
	F1MJ28	Endomembrane system.
	F1MJ28	Sarcoplasm.
	F1MJ28	Sarcoplasmic reticulum.
	F1MJ28	Myofibril.
	F1MJ28	Sarcomere.
	F1MJ28	Sarcomere.
	F1MJ28	Z line.
	F1MJ28	I band.
	F1MJ28	I band.
	F1N197	Protoplasm.
	F1N197	Nucleus.
	F1N197	Nucleoplasm.
	F1N197	Nucleoplasm.
	F1N197	Cytoplasm.
	F1N197	Cytoplasmic granule.
	F1N197	Cytoplasmic granule lumen.
	F1N197	Cytoplasmic granule membrane.
	F1N197	Cytosol.
	F1N5Q7	Protoplasm.
	F1N5Q7	Nucleus.
	F1N5Q7	Nucleoplasm.
	F1N5Q7	Nucleoplasm.
	F1N5Q7	Nuclear body.
	F1N5Q7	Nucleus speckle.
	Q148J8	Protoplasm.
	Q148J8	Cytoplasm.
	Q148J8	Cytoplasmic granule.
	Q148J8	Cytoplasmic granule lumen.
	Q148J8	Cytoplasmic granule membrane.
	Q148J8	Mitochondrion.
	Q148J8	Mitochondrion matrix.
	Q148J8	Myelin membrane.
	Q148J8	Myelin membrane.
	Q1LZF9	Stress fiber.
	Q1LZF9	Extracellular space.

Comparison	Protein	Subcellular localization
	Q1LZF9	Extracellular vesicle.
	Q1LZF9	Extracellular vesicle membrane.
	Q1LZF9	Extracellular vesicle membrane.
	Q1LZF9	Secreted.
	Q1LZF9	Protoplasm.
	Q1LZF9	Cytoplasm.
	Q1LZF9	Cytoplasmic granule.
	Q1LZF9	Cytoplasmic granule lumen.
	Q1LZF9	Cytoplasmic granule membrane.
	Q1LZF9	Spindle.
	Q1LZF9	Cytosol.
	Q1LZF9	Cytoskeleton.
	Q1LZF9	Cell inner membrane.
	Q1LZF9	Cell membrane.
	Q1LZF9	Adherens junction.
	Q1LZF9	Cell cortex.
	Q1LZF9	Membrane.
	Q1LZF9	Apical cell membrane.
	Q1LZF9	Myofibril.
	Q1LZF9	Sarcomere.
	Q1LZF9	Sarcomere.
	Q1LZF9	Z line.
	Q1LZF9	Midbody.
	Q1LZF9	I band.
	Q1LZF9	I band.
	Q1LZF9	Cleavage furrow.
	Q1LZF9	Coated pit.
	Q1LZF9	Cell junction.
	Q1LZF9	Cell junction.
	Q2M2S5	Protoplasm.
	Q2M2S5	Cytoplasm.
	Q2M2S5	Cytoplasmic granule.
	Q2M2S5	Cytoplasmic granule lumen.
	Q2M2S5	Cytoplasmic granule membrane.
	Q2M2S5	Mitochondrion.
	Q2M2S5	Mitochondrion envelope.
	Q2M2S5	Mitochondrion intermembrane space.
	Q2M2S5	Host.
	Q2M2S5	Host.
	Q2M2S5	Host cytoplasm.
	Q2M2S5	Cell envelope.
	Q2M2S5	Cell envelope.
	Q2M2S5	Host microsome.
	Q2M2S5	Host microsome membrane.
	Q2M2S5	Host mitochondrion.
	Q2M2S5	Host cell.
	Q2M2S5	Host mitochondrion envelope.
	Q2M2S5	Target cell.
	Q2M2S5	Target cell cytoplasm.
	Q2M2S5	Target cell membrane.

Comparison	Protein	Subcellular localization
	Q2M2S5	Target membrane.
	Q2M2S5	Tegument.
	Q2M2S5	Tegument membrane.
	Q32PH2	Protoplasm.
	Q32PH2	Cytoplasm.
	Q32PH2	Cytoplasmic granule.
	Q32PH2	Cytoplasmic granule lumen.
	Q32PH2	Cytoplasmic granule membrane.
	Q32PH2	Mitochondrion.
	Q3SZ87	Protoplasm.
	Q3SZ87	Cytoplasm.
	Q3SZ87	Cytoplasmic granule.
	Q3SZ87	Cytoplasmic granule lumen.
	Q3SZ87	Cytoplasmic granule membrane.
	Q3SZ87	Endoplasmic reticulum.
	Q3SZ87	Endoplasmic reticulum membrane.
	Q3SZ87	Endomembrane system.
	Q3SZ87	Membrane.
	Q3SZ93	Protoplasm.
	Q3SZ93	Cytoplasm.
	Q3SZ93	Cytoplasmic granule.
	Q3SZ93	Cytoplasmic granule lumen.
	Q3SZ93	Cytoplasmic granule membrane.
	Q3SZ93	Mitochondrion.
	Q3SZ93	Mitochondrion envelope.
	Q3SZ93	Mitochondrion inner membrane.
	Q3SZ93	Membrane.
	Q3SZ93	Mitochondrion membrane.
	Q3SZ93	Cell envelope.
	Q3SZ93	Cell envelope.
	Q3ZBD4	Protoplasm.
	Q3ZBD4	Nucleus.
	Q3ZBD4	Cytoplasm.
	Q3ZBD4	Cytoplasmic granule.
	Q3ZBD4	Cytoplasmic granule lumen.
	Q3ZBD4	Cytoplasmic granule membrane.
	Q3ZBD4	Adherens junction.
	Q3ZBD4	Myofibril.
	Q3ZBD4	Sarcomere.
	Q3ZBD4	Sarcomere.
	Q3ZBD4	M line.
	Q3ZBD4	M line.
	Q3ZBD4	A band.
	Q3ZBD4	Cell junction.
	Q3ZBD4	Cell junction.
	Q3ZBW7	Protoplasm.
	Q3ZBW7	Cytoplasm.
	Q3ZBW7	Cytoplasmic granule.
	Q3ZBW7	Cytoplasmic granule lumen.
	Q3ZBW7	Cytoplasmic granule membrane.

Comparison	Protein	Subcellular localization
	Q3ZBW7	Mitochondrion.
	Q56K04	Protoplasm.
	Q56K04	Cytoplasm.
	Q56K04	Cytoplasmic granule.
	Q56K04	Cytoplasmic granule lumen.
	Q56K04	Cytoplasmic granule membrane.
	Q8MJ50	Protoplasm.
	Q8MJ50	Cytoplasm.
	Q8MJ50	Cytoplasmic granule.
	Q8MJ50	Cytoplasmic granule lumen.
	Q8MJ50	Cytoplasmic granule membrane.
	Q8MJ50	Endomembrane system.
	Q8MJ50	Vesicle.
	Q8MJ50	Virion.
	Q8MJ50	Cytoplasmic vesicle lumen.
	A0A3Q1LUU1	Protoplasm.
	A0A3Q1LUU1	Nucleus.
	A0A3Q1LUU1	Nucleoplasm.
	A0A3Q1LUU1	Nucleoplasm.
	A0A3Q1LUU1	Cytoplasm.
	A0A3Q1LUU1	Cytoplasmic granule.
	A0A3Q1LUU1	Cytoplasmic granule lumen.
	A0A3Q1LUU1	Cytoplasmic granule membrane.
	A0A3Q1LUU1	Cytosol.
	A0A3Q1LWN4	Protoplasm.
	A0A3Q1LWN4	Nucleus.
	A0A3Q1LWN4	Nucleoplasm.
	A0A3Q1LWN4	Nucleoplasm.
	A0A3Q1LWN4	Cytoplasm.
	A0A3Q1LWN4	Cytoplasmic granule.
	A0A3Q1LWN4	Cytoplasmic granule lumen.
	A0A3Q1LWN4	Cytoplasmic granule membrane.
	A0A3Q1LWN4	Mitochondrion.
	A0A3Q1LWN4	Mitochondrion envelope.
	A0A3Q1LWN4	Mitochondrion inner membrane.
	A0A3Q1LWN4	Membrane.
	A0A3Q1LWN4	Mitochondrion membrane.
	A0A3Q1LWN4	Cell envelope.
	A0A3Q1LWN4	Cell envelope.
	A0A3Q1M390	Protoplasm.
	A0A3Q1M390	Nucleus.
	A0A3Q1M390	Cytoplasm.
	A0A3Q1M390	Cytoplasmic granule.
	A0A3Q1M390	Cytoplasmic granule lumen.
	A0A3Q1M390	Cytoplasmic granule membrane.
	A0A3Q1M390	Myofibril.
	A0A3Q1M390	Sarcomere.
	A0A3Q1M390	Sarcomere.
	A0A3Q1M390	Z line.
	A0A3Q1M390	I band.

Comparison	Protein	Subcellular localization
	A0A3Q1M390	I band.
	A0A3Q1MHP5	Protoplasm.
	A0A3Q1MHP5	Nucleus.
	A0A3Q1MHP5	Cytoplasm.
	A0A3Q1MHP5	Cytoplasmic granule.
	A0A3Q1MHP5	Cytoplasmic granule lumen.
	A0A3Q1MHP5	Cytoplasmic granule membrane.
	A0A3Q1MHP5	Cytosol.
	A0A3Q1MHP5	Cytoskeleton.
	A0A3Q1MHP5	Cell inner membrane.
	A0A3Q1MHP5	Cell membrane.
	A0A3Q1MHP5	Membrane.
	A0A3Q1MJY6	Protoplasm.
	A0A3Q1MJY6	Nucleus.
	A0A3Q1MJY6	Nucleolus.
	A0A3Q1MJY6	Cytoskeleton.
	A0A3Q1MJY6	Cell inner membrane.
	A0A3Q1MJY6	Cell membrane.
	A0A3Q1MJY6	Membrane.
	A0AAA9RYV6	Extracellular space.
	A0AAA9RYV6	Extracellular vesicle.
	A0AAA9RYV6	Extracellular vesicle membrane.
	A0AAA9RYV6	Extracellular vesicle membrane.
	A0AAA9RYV6	Secreted.
	A0AAA9S8T7	Protoplasm.
	A0AAA9S8T7	Nucleus.
	A0AAA9S8T7	Nucleoplasm.
	A0AAA9S8T7	Nucleoplasm.
	A0AAA9S8T7	Nucleolus.
	A0AAA9S8T7	Cytoplasm.
	A0AAA9S8T7	Cytoplasmic granule.
	A0AAA9S8T7	Cytoplasmic granule lumen.
	A0AAA9S8T7	Cytoplasmic granule membrane.
	A0AAA9S8T7	Cytosol.
	A0AAA9S8T7	Cell inner membrane.
	A0AAA9S8T7	Cell membrane.
	A0AAA9S8T7	Membrane.
	A0AAA9S8T7	Nuclear body.
	A0AAA9S8T7	Nucleus speckle.
	A0AAA9SAU9	P-body.
	A0AAA9SAU9	Protoplasm.
	A0AAA9SAU9	Nucleus.
	A0AAA9SAU9	Cytoplasm.
	A0AAA9SAU9	Cytoplasmic granule.
	A0AAA9SAU9	Cytoplasmic granule lumen.
	A0AAA9SAU9	Cytoplasmic granule membrane.
	A0AAA9SAU9	Cytoplasmic ribonucleoprotein granule.
	A0AAA9SAU9	Cytoplasmic ribonucleoprotein granule.
	A0AAA9SAU9	Cytoplasmic vesicle.
	A0AAA9SGV5	Protoplasm.

Comparison	Protein	Subcellular localization
	A0AAA9SGV5	Cytoplasm.
	A0AAA9SGV5	Cytoplasmic granule.
	A0AAA9SGV5	Cytoplasmic granule lumen.
	A0AAA9SGV5	Cytoplasmic granule membrane.
	A0AAA9SGV5	Endoplasmic reticulum.
	A0AAA9SGV5	Endomembrane system.
	A0AAA9SGV5	Perinuclear region.
	A0AAA9SKF6	Protoplasm.
	A0AAA9SKF6	Cytoplasm.
	A0AAA9SKF6	Cytoplasmic granule.
	A0AAA9SKF6	Cytoplasmic granule lumen.
	A0AAA9SKF6	Cytoplasmic granule membrane.
	A0AAA9SKF6	Cytosol.
	A0AAA9SL41	Protoplasm.
	A0AAA9SL41	Cytoplasm.
	A0AAA9SL41	Cytoplasmic granule.
	A0AAA9SL41	Cytoplasmic granule lumen.
	A0AAA9SL41	Cytoplasmic granule membrane.
	A0AAA9SL41	Cytoskeleton.
	A0AAA9SL41	Myofibril.
	A0AAA9SL41	Sarcomere.
	A0AAA9SL41	Sarcomere.
	A0AAA9SL41	Z line.
	A0AAA9SL41	M line.
	A0AAA9SL41	M line.
	A0AAA9SL41	A band.
	A0AAA9SL41	I band.
	A0AAA9SL41	I band.
	A0AAA9SRA8	Stress fiber.
	A0AAA9SRA8	Protoplasm.
	A0AAA9SRA8	Cytoplasm.
	A0AAA9SRA8	Cytoplasmic granule.
	A0AAA9SRA8	Cytoplasmic granule lumen.
	A0AAA9SRA8	Cytoplasmic granule membrane.
	A0AAA9SRA8	Cytosol.
	A0AAA9SRA8	Cytoskeleton.
	A0AAA9SRA8	Cell inner membrane.
	A0AAA9SRA8	Cell membrane.
	A0AAA9SRA8	Adherens junction.
	A0AAA9SRA8	Focal adhesion.
	A0AAA9SRA8	Focal adhesion.
	A0AAA9SRA8	Membrane.
	A0AAA9SRA8	Lamellipodium.
	A0AAA9SRA8	Filopodium.
	A0AAA9SRA8	Axon.
	A0AAA9SRA8	Dendrite.
	A0AAA9SRA8	Filopodium tip.
	A0AAA9SRA8	Cell projection.
	A0AAA9SRA8	Neuron projection.
	A0AAA9SRA8	Synapse.

Comparison	Protein	Subcellular localization
	A0AAA9SRA8	Cell junction.
	A0AAA9SRA8	Cell junction.
	A0AAA9SRA8	Presynapse.
	A0AAA9TA29	Protoplasm.
	A0AAA9TA29	Cytoplasm.
	A0AAA9TA29	Cytoplasmic granule.
	A0AAA9TA29	Cytoplasmic granule lumen.
	A0AAA9TA29	Cytoplasmic granule membrane.
	A0AAA9TA29	Cytosol.
	A0AAA9TA29	Cytoplasmic ribonucleoprotein granule.
	A0AAA9TA29	Cytoplasmic ribonucleoprotein granule.
	A0AAA9TA29	Cytoplasmic vesicle.
	A0AAA9TU40	Protoplasm.
	A0AAA9TU40	Nucleus.
	A0AAA9TU40	Nucleoplasm.
	A0AAA9TU40	Nucleoplasm.
	A0AAA9TU40	Cytoplasm.
	A0AAA9TU40	Cytoplasmic granule.
	A0AAA9TU40	Cytoplasmic granule lumen.
	A0AAA9TU40	Cytoplasmic granule membrane.
	A0AAA9TU40	Cytosol.
	A0AAF6ZGM3	Stress fiber.
	A0AAF6ZGM3	Podosome.
	A0AAF6ZGM3	Podosome membrane.
	A0AAF6ZGM3	Protoplasm.
	A0AAF6ZGM3	Cytoplasm.
	A0AAF6ZGM3	Cytoplasmic granule.
	A0AAF6ZGM3	Cytoplasmic granule lumen.
	A0AAF6ZGM3	Cytoplasmic granule membrane.
	A0AAF6ZGM3	Cytosol.
	A0AAF6ZGM3	Cytoskeleton.
	A0AAF6ZGM3	Cell cortex.
	A0AAF6ZGM3	Myofibril.
	A0AAF6ZGM3	Sarcomere.
	A0AAF6ZGM3	Sarcomere.
	A0AAF6ZGM3	Axon.
	A0AAF6ZGM3	Growth cone.
	A0AAF6ZGM3	Cell projection.
	A0AAF6ZGM3	Neuron projection.
	A4IF72	Protoplasm.
	A4IF72	Nucleus.
	A4IF72	Cytoplasm.
	A4IF72	Cytoplasmic granule.
	A4IF72	Cytoplasmic granule lumen.
	A4IF72	Cytoplasmic granule membrane.
	A4IF72	Mitochondrion.
	A4IF72	Mitochondrion envelope.
	A4IF72	Mitochondrion intermembrane space.
	A4IF72	Cell envelope.
	A4IF72	Cell envelope.

Comparison	Protein	Subcellular localization
	E1BEM3	Protoplasm.
	E1BEM3	Cytoplasm.
	E1BEM3	Cytoplasmic granule.
	E1BEM3	Cytoplasmic granule lumen.
	E1BEM3	Cytoplasmic granule membrane.
	E1BEM3	Cytosol.
	E1BEM3	Cell inner membrane.
	E1BEM3	Cell membrane.
	E1BEM3	Membrane.
	E1BF23	Protoplasm.
	E1BF23	Cytoplasm.
	E1BF23	Cytoplasmic granule.
	E1BF23	Cytoplasmic granule lumen.
	E1BF23	Cytoplasmic granule membrane.
	E1BF23	Cytoskeleton.
	E1BF23	Myofibril.
	E1BF23	Sarcomere.
	E1BF23	Sarcomere.
	E1BF23	Z line.
	E1BF23	M line.
	E1BF23	M line.
	E1BF23	A band.
	E1BF23	I band.
	E1BF23	I band.
	F1MW03	Protoplasm.
	F1MW03	Cytoplasm.
	F1MW03	Cytoplasmic granule.
	F1MW03	Cytoplasmic granule lumen.
	F1MW03	Cytoplasmic granule membrane.
	F1MW03	Cytosol.
	F1MW03	Cytoplasmic ribonucleoprotein granule.
	F1MW03	Cytoplasmic ribonucleoprotein granule.
	F1MW03	Cytoplasmic vesicle.
	F6QVF3	Protoplasm.
	F6QVF3	Nucleus.
	F6QVF3	Nucleoplasm.
	F6QVF3	Nucleoplasm.
	F6QVF3	Cytoplasm.
	F6QVF3	Cytoplasmic granule.
	F6QVF3	Cytoplasmic granule lumen.
	F6QVF3	Cytoplasmic granule membrane.
	F6QVF3	Mitochondrion.
	F6QVF3	Mitochondrion envelope.
	F6QVF3	Mitochondrion intermembrane space.
	F6QVF3	Cell envelope.
	F6QVF3	Cell envelope.

Table S2. Predicted binding energies of four representative phenolic compounds with the selected receptor proteins.

Compound	A0A8V1ACU4 (kcal/mol)	A0A8V1AL60 (kcal/mol)	A0AAA9TBS7 (kcal/mol)	P61603 (kcal/mol)
Caffeic acid	-6.0	-5.9	-6.4	-5.0
Gallic acid	-6.2	-5.8	-6.9	-4.8
Catechin	-7.1	-7.1	-9.4	-6.4
Protocatechuic acid	-6.0	-5.3	-6.2	-4.8

Table S3. Template information and quality assessment parameters of receptor protein models used for molecular docking.

Receptor protein	Template	Sequence identity (%)	Model coverage (%)	GMQE	QMEANDisCo	Most favoured regions (%)	Allowed regions (%)	Disallowed regions (%)
A0A8V1ACU4	8VIZ, chain G	80.77	99.73	0.79	0.74	89.8	99.8	0.2
A0A8V1AL60	AlphaFold DB A0A669PCV6, chain A	86.77	100.00	0.80	NA	91.0	99.2	0.8
A0AAA9TBS7	9ES0, chain F	98.32	95.20	0.88	0.87	95.2	100.0	0.0
P61603	9ES4, chain J	100.00	100.00	0.86	0.81	87.7	100.0	0.0

Note: GMQE, Global Model Quality Estimate; QMEANDisCo, QMEAN distance constraints applied on model quality estimation; NA, not available/not reported by SWISS-MODEL. "Allowed regions" represents the combined percentage of residues located in the most favoured, additional allowed, and generously allowed regions of the Ramachandran plot. Ramachandran plot statistics were obtained using PROCHECK implemented in SAVES v6.0.

Table S4. AutoDock Vina parameters used for blind molecular docking.

Receptor protein	Grid center (x, y, z)	Grid dimensions (x × y × z, Å)	Exhaustiveness	Independent search runs	Maximum poses
A0A8V1ACU4	233.8167, 219.2782, 193.0390	101.8019 × 124.2384 × 101.2914	8	8	9
A0A8V1AL60	5.8090, −4.8123, −0.8591	73.5482 × 89.2539 × 59.3061	8	8	9
A0AAA9TBS7	188.9764, 204.3626, 127.8289	71.6544 × 55.5285 × 85.8383	8	8	9
P61603	154.6732, 167.7466, 282.5348	44.2097 × 32.2578 × 44.4766	8	8	9

Note: Blind docking was performed with the search space encompassing the entire receptor structure. Grid dimensions are expressed in Å along the x-, y-, and z-axes, respectively. The exhaustiveness parameter was set to 8. Up to nine binding poses were retained per docking calculation.

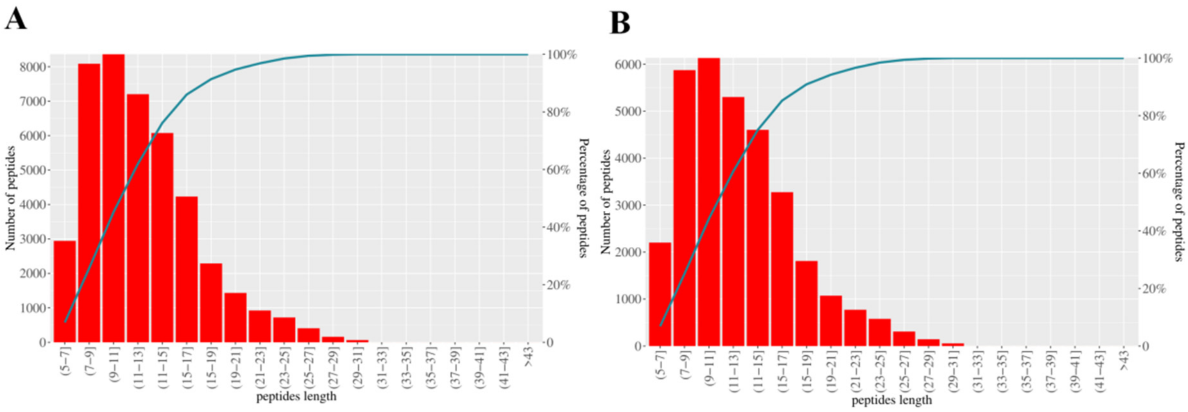

Fig. S1. Peptide length distribution in proteomic analysis.(A) Peptide length distribution in the chicken group. (B) Peptide length distribution in the beef group. Red bars indicate the number of peptides at each peptide length, and the blue line indicates the cumulative percentage of peptides.

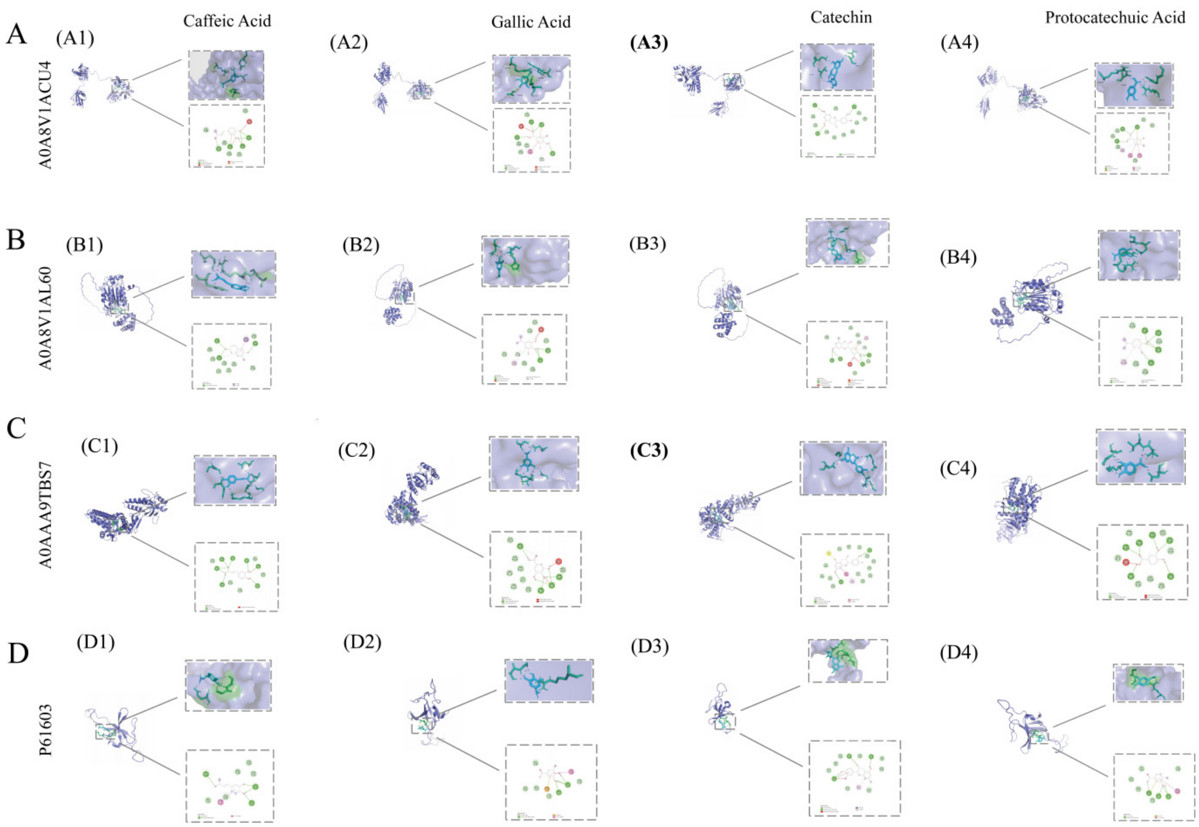

Fig. S2. Three-dimensional (3D) binding conformations and two-dimensional (2D) interaction diagrams of all 16 molecular docking combinations between four representative phenolic compounds from A. tsaoko and four selected receptor proteins. Rows A–D correspond to A0A8V1ACU4, A0A8V1AL60, A0AAA9TBS7, and P61603, respectively. Columns 1–4 correspond to caffeic acid, gallic acid, catechin, and protocatechuic acid, respectively. Panels A3 and C3 show the representative catechin–A0A8V1ACU4 and catechin–A0AAA9TBS7 docking complexes, respectively, which are presented in enlarged form in Fig. 6 of the main manuscript.
